# Dielectric and Elastic Characterization of Nonlinear Heterogeneous Materials

**DOI:** 10.3390/ma2041417

**Published:** 2009-09-30

**Authors:** Stefano Giordano

**Affiliations:** 1 Department of Physics, University of Cagliari, Cittadella Universitaria, I-09042 Monserrato, Italy; E-Mails: stefano.giordano@dsf.unica.it; stefgiord14@libero.it; Tel.: +39-070-675-4847; Fax: +39-070-510171; 2 Sardinian Laboratory for Computational Materials Science (SLACS, INFM-CNR), Cittadella Universitaria Monserrato, SP Monserrato-Sestu Km 0.700, I-09042 Monserrato, Italy

**Keywords:** nonlinear constitutive equations, composites (nanosystems embedded in a larger structure), mixture theory and order parameters

## Abstract

This review paper deals with the dielectric and elastic characterization of composite materials constituted by dispersions of nonlinear inclusions embedded in a linear matrix. The dielectric theory deals with pseudo-oriented particles shaped as ellipsoids of revolution: it means that we are dealing with mixtures of inclusions of arbitrary aspect ratio and arbitrary non-random orientational distributions. The analysis ranges from parallel spheroidal inclusions to completely random oriented inclusions. Each ellipsoidal inclusion is made of an isotropic dielectric material described by means of the so-called Kerr nonlinear relation. On the other hand, the nonlinear elastic characterization takes into consideration a dispersion of nonlinear (spherical or cylindrical) inhomogeneities. Both phases are considered isotropic (actually it means polycrystalline or amorphous solids). Under the simplifying hypotheses of small deformation for the material body and of small volume fraction of the embedded phase, we describe a theory for obtaining the linear and nonlinear elastic properties (bulk and shear moduli and Landau coefficients) of the overall material.

## 1. Introduction

The central problem of considerable technological importance is to evaluate the effective physical properties (dielectric or elastic) governing the behavior of a composite material on the macroscopic scale, taking into account the actual microscale material features [1,2]. At present, it is well known that it does not exist a universal mixing formula giving the effective properties of the heterogeneous materials (permittivity or elastic moduli) as some sort of average of the properties of the constituents. In fact, the details of the morphology or micro-geometry play a central role in determining the overall properties, particularly when the crystalline grains have highly anisotropic or nonlinear behavior or when there is a large difference in the properties of the constituent materials. The primary aim in the study of materials is to understand and classify the relationship between the internal micro-structure and the physical properties. Such a relationship may be used for designing and improving materials or, conversely, for interpreting experimental data in terms of micro-structural features. A great number of theoretical investigations have been developed in order to describe the behavior of composite materials when a specific microstructure is considered. On the other hand, a different class of theories does not assume a given microstructure, searching for general results of broad applicability. The most important properties are the classical Hashin-Shtrikman variational bounds [3,4], which provide an upper and lower bound for composite materials properties, and the expansions of Brown [5] and Torquato [6,7] which take into account the spatial correlation function of the constituents. Moreover, in [8], a functional unifying approach has been applied to better understand the intrinsic mathematical properties of a general mixing formula.

Dispersions or suspensions of inhomogeneities in a matrix are examples of widely studied heterogeneous materials: these media have been extensively analyzed both from the electrical and the elastic point of view. One of the first attempts to characterize dielectric dispersions of spheres was developed by Maxwell [9,10], who found out a famous formula valid for very diluted suspensions. The first papers dealing with mixtures of ellipsoids were written by Fricke [11,12] dealing with the electrical characterization of inhomogeneous biological tissues containing spheroidal particles: he found some explicit relationships that were simply an extension of the Maxwell formula to the case with ellipsoidal inclusions. In current literature, Maxwell relation for spheres and Fricke expressions for ellipsoids are the so-called Maxwell–Garnett Effective Medium Theories (MG-EMT) [13,14]: both theories hold on under the hypothesis of very low concentration of the dispersed component. A better model has been provided by the differential scheme [15,16]. In this case the results maintain the validity also for less diluted suspensions [17].

Some other types of microstructures have been taken into consideration. For example, the problem of the mixture characterization has been exactly solved in the case of linear and nonlinear random mixtures, that is, materials for which the various components are isotropic, linear and mixed together as an ensemble of particles having random shapes and positions (in this case there is not a material having the role of a matrix and all the media have the same importance in defining the overall properties) [18,19,20,21,22].

Recent progresses in this field concern dielectrically linear and nonlinear spheroidal inhomogeneities with geometric factors probabilistically distributed [23]. The size-dependent Bruggeman theory, which considers the effective particle dimension for non dilute dispersions, has been introduced as well [24]. A wide survey of mixture theory applications to metamaterials can be found in [25]. Finally, the dielectric (focusing or defocusing) Kerr nonlinearity [26] has been utilized to explore the importance of the particle shape [27].

On the other hand, dealing with the elastic characterization of dispersions, a similar line of research has been developed [28,29]. A famous result exists for a material composed by a very dilute concentration of linear spherical inhomogeneities dispersed in a linear solid matrix [30]. To adapt this theory to the case of any finite volume fraction, the differential method is also applied to the elastic theories for spherical [31], cylindrical [32] and ellipsoidal particles [33]. Recent works focus on microstructures that can be characterized as continuous matrices containing inhomogeneities of diverse shapes, properties and orientations [34,35]. The evaluation of the effective elastic properties of a body containing a given distribution of cracks belongs to the field of homogenization techniques as well [36]. Recent investigations consider the effects of the orientational statistical distribution of cracks in a given material [37,38].

The aim of the present review paper is to describe the dielectric and elastic characterization of composite materials constituted by dispersions of linear or nonlinear inclusions embedded in a linear matrix. The complete dielectric theory deals with pseudo-oriented particles shaped as ellipsoids of revolution: it means that we are dealing with mixtures of inclusions of arbitrary aspect ratio and arbitrary non-random orientational distributions. Moreover, the nonlinear elastic characterization takes into consideration a dispersion of nonlinear (spherical or cylindrical) inhomogeneities. Under the simplifying hypotheses of small deformation for the material body and of small volume fraction of the embedded particles, we describe a theory for obtaining the linear and nonlinear elastic properties of the overall material.

The paper is structured as follows: in Section 2 we present a brief outline of the most important results describing the dielectric homogenization techniques for linear and nonlinear dispersions. In particular, in Section 2.1. we describe the methodologies for linear and nonlinear two-phases materials (i.e., linear or nonlinear homogeneous inclusions embedded in a linear homogeneous matrix). Moreover, in Section 2.2. we describe the methods applied to investigate the three-phases materials (i.e., coated or core-shell inclusions embedded in a given matrix). We have unified all the specific results in a single framework, which is suitable for both two- and three- dimensional systems. These results are well-known in scientific literature and they are introduced here for sake of completeness. Therefore, all the relevant references have been accurately quoted in order to facilitate the interested reader.

In Section 3 we describe a microstructure constituted by pseudo-oriented ellipsoids, which is important since mimics several real materials and exhibits an overall behavior depending on the combination of two different aspects: the degree of ordering of the system (i.e., the degree of orientational distribution of the ellipsoidal particles inside the medium) and the aspect ratio (controlling the shape of the inclusion ranging from oblate to prolate spheroids) of the ellipsoids embedded in the matrix. In the following Section 4, Section 5, Section 6 and Section 7 the corresponding nonlinear electric homogenization is introduced and developed. The final equations obtained at the end of the procedure are original achievements of the present work. However, several intermediate results can be found in earlier literature and will be discussed in detail in order to provide a complete review. More precisely, in Section 4 we discuss the results concerning the electrical behavior of a single nonlinear ellipsoid embedded in a linear matrix: the general theory has been presented for an arbitrary nonlinearity and the application has been performed for the so-called Kerr nonlinear constitutive equation. In Section 5 the electric field induced inside the ellipsoidal particle has been averaged over all the possible orientations of the particle itself. This calculation is original and it has been developed for a Kerr nonlinearity. Then, in Section 6 we consider a dilute suspension of randomly oriented ellipsoidal inclusions and we obtain the dielectric linear and nonlinear characterization in terms of the degree of order of the system and the aspect ratio of the particles. Finally, in Section 7 we show an example of application of the previous theory and we discuss the behavior of the effective permittivities and of the nonlinear susceptibilities.

In Section 8 we introduce the homogenization for nonlinear elastic composite materials. In particular we describe the microstructures analyzed in the following sections and we introduce the applied methodologies. This second part of the paper represents a detailed review of recent results in the field of the nonlinear homogenization (the relevant references will be properly quoted). In Section 9 we discuss the nonlinear constitutive equations adopted to model the embedded particles (for the nonlinear elastic homogenization scheme we consider for simplicity spherical or cylindrical inhomogeneities). In Section 10 we introduce the nonlinear generalization of the Eshelby theory, which allows us to determine the elastic fields induced inside an inhomogeneity. Finally, in Section 11 and Section 12 we develop the procedures for dealing, respectively, with dispersions of spheres and cylinders.

## 2. Introductory Remarks on Linear and Nonlinear Dielectric Homogenization

In order to introduce the standard homogenization techniques for linear and nonlinear heterogeneous structures, we consider a two-phases material (spheres or cylinders embedded in a given matrix) and a three-phases material (coated spheres or cylinders embedded in a given matrix). In both cases we analyze the electric behavior of the single particle and of a given assembly of inclusion.

### 2.1. Two-phases materials

We describe the linear electric behavior of a given spherical (d=3) or cylindrical (d=2) particle with permittivity $\epsilon_{2}$ and radius *R* embedded in a matrix with permittivity $\epsilon_{1}$. For the spherical object we adopt a system of spherical coordinates (ρ,θ,ϕ) (x=ρsinθcosϕ,y=ρsinθsinϕ,z=ρcosθ) and for the cylindrical particle we use a system of cylindrical coordinates (ρ,θ,y) (x=ρsinθ,y=y,z=ρcosθ). We suppose to apply a remote electric field $E^{\infty }$ along the *z*-axis in both cases. The electric potentials inside and outside the inclusion, $\phi_{i}$ and $\phi_{e}$, are given by [14,15]

(1) $$\begin{array}{ccc} \phi_{i} & = & -E^{\infty }\left[\frac{d\epsilon_{1}}{\epsilon_{2}+\left(d-1\right)\epsilon_{1}}\right]\rho \cos\theta \end{array}$$

(2) $$\begin{array}{ccc} \phi_{e} & = & -E^{\infty }\left[1-\frac{\epsilon_{2}-\epsilon_{1}}{\epsilon_{2}+\left(d-1\right)\epsilon_{1}}{\left(\frac{R}{\rho }\right)}^{d}\right]\rho \cos\theta \end{array}$$

Therefore, the electric field induced inside a particle is

(3) $$E_{i}=\left[\frac{d\epsilon_{1}}{\epsilon_{2}+\left(d-1\right)\epsilon_{1}}\right]E^{\infty }$$

**Figure 1:**
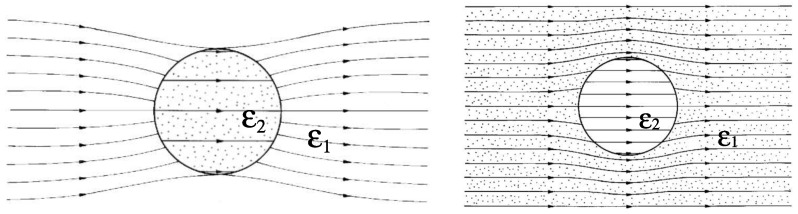
Field lines of the electric field for an inclusion with $\epsilon_{2}>\epsilon_{1}$ and for another one with $\epsilon_{2}<\epsilon_{1}$.

This internal field is called Lorentz field and it is always uniform: it is possible to prove that $E_{i}>E^{\infty }$ if $\epsilon_{2}<\epsilon_{1}$ and $E_{i}<E^{\infty }$ if $\epsilon_{2}>\epsilon_{1}$. In Figure 1 one can observe the lines of the electric field for an inclusion with $\epsilon_{2}>\epsilon_{1}$ and for the case $\epsilon_{2}<\epsilon_{1}$. In both cases the uniformity of the internal field and the dipolar character of the external one are evident.

We consider now a dispersion of particles in a given region. We assume that the volume fraction *c* of inclusions is very low (c≪1): it means that Equation (3) is valid for each particle and that the average value of the electric field over the entire structure can be obtained as $\langle E\rangle =cE_{i}+\left(1-c\right)E^{\infty }$. In similar way it is possible to calculate the average value of the displacement vector as $\langle D\rangle =c\left(\epsilon_{2}-\epsilon_{1}\right)E_{i}+\epsilon_{1}\langle E\rangle$. We define the concept of effective permittivity $\epsilon_{eff}$ through the relation $\langle D\rangle =\epsilon_{eff}\langle E\rangle$. The above expressions allow us to obtain the following important result (Maxwell formula) [9]

(4) $$\begin{array}{c} \epsilon_{eff}=\epsilon_{1}\frac{c\epsilon_{2}d+\left(1-c\right)\left[\epsilon_{2}+\left(d-1\right)\epsilon_{1}\right]}{c\epsilon_{1}d+\left(1-c\right)\left[\epsilon_{2}+\left(d-1\right)\epsilon_{1}\right]} \end{array}$$

This is the main result concerning the linear homogenization of an assembly of spherical (d=3) or cylindrical (d=2) particles.

We consider now the nonlinear behavior of a single inclusion. This methodology has been developed in [39] and it has been utilized both for a single particle and an assembly of nonlinear spheres. The benefit of this approach is that of considering an arbitrary nonlinearity describing the electric behavior of the inhomogeneities embedded in the matrix. The nonlinear constitutive relation for the particle can be written as $D_{i}={\tilde{\epsilon }}_{2}\left(E_{i}\right)E_{i}$ where ${\tilde{\epsilon }}_{2}\left(E_{i}\right)$ is the field-dependent permittivity. The important Equation (3) is still valid but now it is an implicit equation giving the actual internal field ${\tilde{E}}_{i}$

(5) $$\begin{array}{c} {\tilde{E}}_{i}=\left[\frac{d\epsilon_{1}}{{\tilde{\epsilon }}_{2}\left({\tilde{E}}_{i}\right)+\left(d-1\right)\epsilon_{1}}\right]E^{\infty } \end{array}$$

Therefore, the balance equations for a dilute dispersion of nonlinear particles are the following

(6) $$\begin{array}{c} \left\{\begin{array}{ccc} {\tilde{E}}_{i} & = & \frac{d\epsilon_{1}}{{\tilde{\epsilon }}_{2}\left({\tilde{E}}_{i}\right)+\left(d-1\right)\epsilon_{1}}E^{\infty }\\ \langle E\rangle & = & c{\tilde{E}}_{i}+\left(1-c\right)E^{\infty }\\ \langle D\rangle & = & c\left(\epsilon_{2}-\epsilon_{1}\right){\tilde{E}}_{i}+\epsilon_{1}\langle E\rangle \end{array}\right. \end{array}$$

From the first two expressions in Equation (6) we can eliminate the remote field $E^{\infty }$, by obtaining an explicit relationship between the internal field ${\tilde{E}}_{i}$ and the average value 〈E〉

(7) $$\begin{array}{c} {\tilde{E}}_{i}=\frac{d\epsilon_{1}}{{\tilde{\epsilon }}_{2}\left({\tilde{E}}_{i}\right)+\left(d-1\right)\epsilon_{1}}\frac{\langle E\rangle -c{\tilde{E}}_{i}}{1-c} \end{array}$$

Now, it is necessary to solve Equation (7) with respect to ${\tilde{E}}_{i}$ for different values of the average electric field 〈E〉 (nonlinear microdosimetry). This point can be accomplished with different numerical methods depending on the complexity of the nonlinearity ${\tilde{\epsilon }}_{2}\left({\tilde{E}}_{i}\right)$. If we substitute Equation (7) in the last expression given in Equation (6) we obtain the nonlinear effective constitutive equation

(8) $$\begin{array}{c} \langle D\rangle =\epsilon_{1}\left[1+cd\frac{\langle E\rangle -{\tilde{E}}_{i}}{\left(1-c\right)\langle E\rangle }\right]\langle E\rangle \end{array}$$

Therefore, the effective nonlinear dielectric constant (depending on 〈E〉) is given by

(9) $$\begin{array}{c} \epsilon_{eff}\left(\langle E\rangle \right)=\epsilon_{1}\left[1+cd\frac{\langle E\rangle -{\tilde{E}}_{i}\left(\langle E\rangle \right)}{\left(1-c\right)\langle E\rangle }\right] \end{array}$$

The explicit form of ${\tilde{E}}_{i}$ in terms of 〈E〉 (which can be obtained numerically or analytically from Equation (7)) must be substituted in Equation (9) in order to obtain the nonlinear effective behavior. This method can be applied to the case of a Kerr nonlinearity for the particles [27]

(10) $$\begin{array}{ccc} {\tilde{\epsilon }}_{2}\left({\dot{E}}_{i}\right) & = & {\dot{\epsilon }}_{2}+\dot{\alpha }{\left|{\dot{E}}_{i}\right|}^{2} \end{array}$$

where the dots indicate the phasors of the corresponding quantities (we assume a sinusoidal permanent regime). In this case both $\epsilon_{2}$ and *α* can be considered complex numbers. We may substitute Equation (10) in Equation (7), obtaining

(11) $$\begin{array}{c} \dot{\alpha }X{\left|X\right|}^{2}+\frac{\left(1-c\right)\left({\dot{\epsilon }}_{2}-\epsilon_{1}\right)+\epsilon_{1}d}{1-c}X-\frac{\epsilon_{1}d}{1-c}\langle \dot{E}\rangle =0 \end{array}$$

where we have defined ${\dot{E}}_{i}=X$ (the main unknown). This equation assumes the simple form

(12) $$\begin{array}{c} AX+{BX|X|}^{2}=Y \end{array}$$

where $A=\left({\dot{\epsilon }}_{2}-\epsilon_{1}\right)\left(1-c\right)+d\epsilon_{1}$, $B=\left(1-c\right)\dot{\alpha }$ and $Y=d\epsilon_{1}\langle \dot{E}\rangle$. The solution can be approximately obtained through the following expansion truncated after the third odd term

(13) $$\begin{array}{c} X=a_{1}Y+a_{2}{Y|Y|}^{2}+a_{3}Y{\left|Y\right|}^{4} \end{array}$$

By using Equation (13) in Equation (12) we obtain the values for the coefficients

(14) $$\begin{array}{c} a_{1}=\frac{1}{A}\;\;\;\;\;\;\;\;a_{2}=-\frac{b}{A^{2}{\left|A\right|}^{2}}\;\;\;\;\;\;\;\;a_{3}=\frac{2B^{2}}{A^{3}{\left|A\right|}^{4}}+\frac{{\left|B\right|}^{2}}{{A|A|}^{6}} \end{array}$$

Finally, adopting the value of ${\dot{E}}_{i}=X$ in Equation (9) we obtain the nonlinear homogenization

(15) $$\begin{array}{c} \varepsilon_{eff}={\dot{\epsilon }}_{eff}+{\dot{\alpha }}_{eff}|\langle \dot{E}\rangle {|}^{2}+{\dot{\beta }}_{eff}{\left|\langle \dot{E}\rangle \right|}^{4} \end{array}$$

where [27]

(16) $$\begin{array}{ccc} {\dot{\epsilon }}_{eff} & = & \epsilon_{1}\frac{c{\dot{\epsilon }}_{2}d+\left(1-c\right)\left[{\dot{\epsilon }}_{2}+\left(d-1\right)\epsilon_{1}\right]}{c\epsilon_{1}d+\left(1-c\right)\left[{\dot{\epsilon }}_{2}+\left(d-1\right)\epsilon_{1}\right]}\\ {\dot{\alpha }}_{eff} & = & \frac{cd^{4}\dot{\alpha }}{{\left(\frac{A}{\epsilon_{1}}\right)}^{2}{\left|\frac{A}{\epsilon_{1}}\right|}^{2}}\\ {\dot{\beta }}_{eff} & = & -2\frac{c\left(1-c\right)d^{6}{\dot{\alpha }}^{2}}{\epsilon_{1}{\left(\frac{A}{\epsilon_{1}}\right)}^{3}{\left|\frac{A}{\epsilon_{1}}\right|}^{4}}-\frac{c\left(1-c\right)d^{6}{\left|\dot{\alpha }\right|}^{2}}{\frac{|\epsilon_{1}{|}^{2}}{\epsilon_{1}}\frac{A}{\epsilon_{1}}{\left|\frac{A}{\epsilon_{1}}\right|}^{6}} \end{array}$$

Here we have used the definition $A=\left({\dot{\epsilon }}_{2}-\epsilon_{1}\right)\left(1-c\right)+d\epsilon_{1}$. The first expression in Equation (16) represents the Maxwell formula describing the linear behavior of the composite system. The second relation in Equation (16) represents the Kerr nonlinear coefficient of the dispersion: we observe that it is directly proportional to the volume fraction *c* and to the Kerr parameter $\dot{\alpha }$ of the inclusions. When c→1 the value of ${\dot{\epsilon }}_{eff}$ approaches ${\dot{\epsilon }}_{2}$ and ${\dot{\alpha }}_{eff}$ approaches $\dot{\alpha }$, as expected. Finally, the coefficient ${\dot{\beta }}_{eff}$ represents the fourth nonlinear term of the heterogeneous structure. We note that it is proportional to $c\left(1-c\right)$: therefore, it is zero for c=0 (no inclusions in the system) and for c=1 (the inclusions fill the whole space). Anyway, we remember that all the results are valid for dilute dispersions (c≪1). Similar results and some generalizations can be found in [40,41,42].

### 2.2. Three-phases materials

We consider a spherical (or cylindrical) structure constituted by a matrix with permittivity $\epsilon_{1}$ where a coated particle is embedded. The particle is composed by a core with radius *a* and permittivity $\epsilon_{3}$ and a shell contained between the radii *a* and *b* with permittivity $\epsilon_{2}$. On such a system an electric field $E^{\infty }=E^{\infty }e_{z}$ is remotely applied. The electric potentials in the three regions can be calculated as [39,43]

(17) $$\begin{array}{c} \begin{array}{ccc} \phi_{1}= & -E^{\infty }\rho \cos\theta +A\frac{1}{\rho^{d-1}}\cos\theta \; & \text{if}\;\rho >b\\ \phi_{2}= & B\rho \cos\theta +C\frac{1}{\rho^{d-1}}\cos\theta \; & \text{if}\;a<\rho <b\\ \phi_{3}= & D\rho \cos\theta \; & \text{if}\;\rho <a \end{array} \end{array}$$

As before, these expression are valid both in cylindrical symmetry (d=2) and in spherical symmetry (d=3). The coefficients A,B,C,D can be obtained by imposing the continuity of the electric potential and of the normal component of the electric displacement. These conditions allows us to obtain the system of equations

(18) $$\begin{array}{c} \left\{\begin{array}{cc} C & =\left(D-B\right)a^{d}\\ \left(\epsilon_{2}B-\epsilon_{3}D\right)a^{d} & =\left(d-1\right)\epsilon_{2}C\\ A-C & =\left(B+E^{\infty }\right)b^{d}\\ \left(d-1\right)\left(C\epsilon_{2}-A\epsilon_{1}\right) & =\left(B\epsilon_{2}+E^{\infty }\epsilon_{1}\right)b^{d} \end{array}\right. \end{array}$$

The exact solutions have been eventually obtained [39]

(19) $$\begin{array}{ccc} A & = & \frac{\left(\epsilon_{1}-\epsilon_{2}\right)\left[\left(d-1\right)\epsilon_{2}+\epsilon_{3}\right]{\left(\frac{b}{a}\right)}^{d}-\left(\epsilon_{3}-\epsilon_{2}\right)\left[\left(d-1\right)\epsilon_{2}+\epsilon_{1}\right]}{\left(d-1\right)\left(\epsilon_{1}-\epsilon_{2}\right)\left(\epsilon_{3}-\epsilon_{2}\right)-\left[\left(d-1\right)\epsilon_{1}+\epsilon_{2}\right]\left[\left(d-1\right)\epsilon_{2}+\epsilon_{3}\right]{\left(\frac{b}{a}\right)}^{d}}E^{\infty }b^{d}\\ B & = & \frac{d\epsilon_{1}\left[\left(d-1\right)\epsilon_{2}+\epsilon_{3}\right]{\left(\frac{b}{a}\right)}^{d}}{\left(d-1\right)\left(\epsilon_{1}-\epsilon_{2}\right)\left(\epsilon_{3}-\epsilon_{2}\right)-\left[\left(d-1\right)\epsilon_{1}+\epsilon_{2}\right]\left[\left(d-1\right)\epsilon_{2}+\epsilon_{3}\right]{\left(\frac{b}{a}\right)}^{d}}E^{\infty }\\ C & = & \frac{-d\epsilon_{1}\left(\epsilon_{3}-\epsilon_{2}\right)}{\left(d-1\right)\left(\epsilon_{1}-\epsilon_{2}\right)\left(\epsilon_{3}-\epsilon_{2}\right)-\left[\left(d-1\right)\epsilon_{1}+\epsilon_{2}\right]\left[\left(d-1\right)\epsilon_{2}+\epsilon_{3}\right]{\left(\frac{b}{a}\right)}^{d}}E^{\infty }b^{d}\\ D & = & \frac{d^{2}\epsilon_{1}\epsilon_{2}{\left(\frac{b}{a}\right)}^{d}}{\left(d-1\right)\left(\epsilon_{1}-\epsilon_{2}\right)\left(\epsilon_{3}-\epsilon_{2}\right)-\left[\left(d-1\right)\epsilon_{1}+\epsilon_{2}\right]\left[\left(d-1\right)\epsilon_{2}+\epsilon_{3}\right]{\left(\frac{b}{a}\right)}^{d}}E^{\infty } \end{array}$$

To begin with the homogenizing schemes, we search for a homogeneous dielectric material (with permittivity $\tilde{\epsilon }$) in the region 0<ρ<b, which is equivalent to the structure above described. It means that the substitution of the regions with $\epsilon_{2}$ and $\epsilon_{3}$ with a single homogeneous particle with permittivity $\tilde{\epsilon }$ does not alter the values of the electric potential and of the electric field in the external region ρ>b. In the original heterogeneous structure the external electric potential is given by $\phi_{1}$ reported in Equation (17). In the equivalent case with a single uniform inhomogeneity this electric potential is described by the same expression where $A=A{|}_{\epsilon_{2}=\tilde{\epsilon },\;\epsilon_{3}=\tilde{\epsilon }}$ since the theory is still correct for $\epsilon_{2}=\epsilon_{3}=\tilde{\epsilon }$. By equating the values $A\left(\epsilon_{1},\epsilon_{2},\epsilon_{3}\right)=A\left(\epsilon_{1},\tilde{\epsilon },\tilde{\epsilon }\right)$, we obtain an equation for the unknown $\tilde{\epsilon }$. The solution is

(20) $$\begin{array}{c} \tilde{\epsilon }=\epsilon_{2}\frac{\left(d-1\right)\left(\epsilon_{2}-\epsilon_{3}\right)-{\left(\frac{b}{a}\right)}^{d}\left[\left(d-1\right)\epsilon_{2}+\epsilon_{3}\right]}{\left(\epsilon_{3}-\epsilon_{2}\right)-{\left(\frac{b}{a}\right)}^{d}\left[\left(d-1\right)\epsilon_{2}+\epsilon_{3}\right]} \end{array}$$

We remark that this value of the effective permittivity does not depend on the permittivity of the matrix. In fact, it is a characteristic quantity of the heterogeneous structure (constituted by the core and the shell) embedded in the homogeneous material with permittivity $\epsilon_{1}$. Moreover, it depends on the ratio between the radius of the core and the radius of the shell. Therefore, we can define the volume fraction of the core of permittivity $\epsilon_{3}$ in the shell of permittivity $\epsilon_{2}$ as $\tilde{c}={\left(\frac{a}{b}\right)}^{d}$. Therefore, Equation (20) assumes the very simple form

(21) $$\begin{array}{c} \tilde{\epsilon }=\epsilon_{2}\frac{\tilde{c}\epsilon_{3}d+\left(1-\tilde{c}\right)\left[\epsilon_{3}+\left(d-1\right)\epsilon_{2}\right]}{\tilde{c}\epsilon_{2}d+\left(1-\tilde{c}\right)\left[\epsilon_{3}+\left(d-1\right)\epsilon_{2}\right]} \end{array}$$

This is an exact result. It is interesting to note that Equation (21) is formally identical to the Maxwell formula, which describes the effective permittivity of a dilute dispersion of inclusions of permittivity $\epsilon_{3}$ in a hosting material of permittivity $\epsilon_{2}$. Nevertheless, the Maxwell formula is an approximate solution which is valid only for low values of the volume fraction $\tilde{c}$ of the spheres embedded in the matrix.

We have obtained a uniform electric field in the core (with intensity -D) and a more complicated spatial behavior in the shell. For the following development it is useful to calculate the average value of the electric field in the shell [39]. More precisely, we search for the average value of $E_{2}=-\vec{\nabla }\phi_{2}$ over the region a<ρ<b

(22) $$\begin{array}{c} \langle E_{2}\rangle =\frac{1}{\Omega }\int_{\Omega }-\vec{\nabla }\phi_{2}dv \end{array}$$

where Ω is the region corresponding to the shell (or coating). When d=2, we solved the double integral

(23) $$\begin{array}{c} \langle E_{2}\rangle =\frac{1}{\pi \left(b^{2}-a^{2}\right)}\int \int_{a^{2}\leq x^{2}+z^{2}\leq b^{2}}\left(-\frac{\partial \phi_{2}}{\partial x},-\frac{\partial \phi_{2}}{\partial z}\right)dxdz=\left(0,-B\right) \end{array}$$

Moreover, for d=3 we obtained

(24) $$\begin{array}{c} \langle E_{2}\rangle =\frac{1}{\frac{4}{3}\pi \left(b^{3}-a^{3}\right)}\int \int \int_{a^{2}\leq x^{2}+y^{2}+z^{2}\leq b^{2}}\left(-\frac{\partial \phi_{2}}{\partial x},-\frac{\partial \phi_{2}}{\partial y},-\frac{\partial \phi_{2}}{\partial z}\right)dxdydz=\left(0,0,-B\right) \end{array}$$

It means that, in each case, the relation $\langle E_{2}\rangle =-Be_{z}$ is always fulfilled.

Now, we consider a dispersion of coated particles in the linear electric regime. We utilize the property which homogenizes each particle, in order to apply the Maxwell formula in a second step. We define $c=\frac{V_{d}}{V}=\frac{V_{d}}{V_{d}+V_{f}}\ll 1$ where *V* is the total volume of the material, $V_{d}$ is the total volume of the effective inclusions with permittivity $\tilde{\epsilon }$ and $V_{f}$ is the volume of the external region with permittivity $\epsilon_{1}$. At the end of this procedure we obtain

(25) $$\begin{array}{c} \epsilon_{eff}=\epsilon_{1}\frac{c\tilde{\epsilon }d+\left(1-c\right)\left[\tilde{\epsilon }+\left(d-1\right)\epsilon_{1}\right]}{c\epsilon_{1}d+\left(1-c\right)\left[\tilde{\epsilon }+\left(d-1\right)\epsilon_{1}\right]} \end{array}$$

where $\tilde{\epsilon }$ is given in Equation (21). The optical particles of core-shell particle composites has been also analyzed for the confocal ellipsoids configuration: interested readers can consult [44,45] for more information.

This procedure can be generalized to the case with a nonlinear core in the particles [39,46,47]. This model has been applied to investigate the optical bistability of suspensions of nonlinear coated nanoparticles [48,49] and the orientation of core-shell inclusions in an electric field [50]. We suppose that each core can be described by a Kerr nonlinearity $\tilde{\epsilon_{3}}=\epsilon_{3}+\chi {\left|E_{3}\right|}^{2}$, where $E_{3}$ is the electric field for ρ<a (for simplicity we omit the dots indicating the phasors). From the above discussion we obtain $E_{3}=-D$ where *D* is given in Equation (19). In nonlinear regime, the coefficient *D* is not constant since it depends on $\tilde{\epsilon_{3}}$ and, therefore, on the core field $E_{3}$. Explicitly

(26) $$\begin{array}{c} E_{3}=\frac{d^{2}\epsilon_{1}\epsilon_{2}E^{\infty }}{-\left(d-1\right)\left(\epsilon_{1}-\epsilon_{2}\right)\left({\tilde{\epsilon }}_{3}-\epsilon_{2}\right){\left(\frac{a}{b}\right)}^{d}+\left[\left(d-1\right)\epsilon_{1}+\epsilon_{2}\right]\left[\left(d-1\right)\epsilon_{2}+{\tilde{\epsilon }}_{3}\right]} \end{array}$$

By recalling the definition $\tilde{c}={\left(\frac{a}{b}\right)}^{d}$ we can write the denominator of the previous relation as

$$\begin{array}{c} \epsilon_{2}\left(d-1\right)\left\{\left[\left(d-1\right)\epsilon_{1}+\epsilon_{2}\right]+\tilde{c}\left(\epsilon_{1}-\epsilon_{2}\right)\right\}+\left\{\left(d-1\right)\left(\epsilon_{2}-\epsilon_{1}\right)\tilde{c}+\left[\left(d-1\right)\epsilon_{1}+\epsilon_{2}\right]\right\}{\tilde{\epsilon }}_{3}=\alpha +\beta {\tilde{\epsilon }}_{3} \end{array}$$

where we have defined the constants *α* and *β* as follows

(27) $$\begin{array}{ccc} \alpha & = & \epsilon_{2}\left(d-1\right)\left\{\left[\left(d-1\right)\epsilon_{1}+\epsilon_{2}\right]+\tilde{c}\left(\epsilon_{1}-\epsilon_{2}\right)\right\} \end{array}$$

(28) $$\begin{array}{ccc} \beta & = & \left\{\left(d-1\right)\left(\epsilon_{2}-\epsilon_{1}\right)\tilde{c}+\left[\left(d-1\right)\epsilon_{1}+\epsilon_{2}\right]\right\} \end{array}$$

Now, Equation (26) can be written in the simple form

(29) $$\begin{array}{c} E_{3}=\frac{d^{2}\epsilon_{1}\epsilon_{2}}{\alpha +\beta {\tilde{\epsilon }}_{3}}E^{\infty }=\frac{d^{2}\epsilon_{1}\epsilon_{2}}{\alpha +\beta \left(\epsilon_{3}+\chi {\left|E_{3}\right|}^{2}\right)}E^{\infty } \end{array}$$

At this point it is useful to obtain the average value of the electric field inside the composite material. We develop our calculations along the *z* direction. The value of $\langle E_{z}\rangle$ can be obtained as follows

(30) $$\begin{array}{ccc} \langle E_{z}\rangle & = & \left(1-c\right)E^{\infty }+c\left(1-\tilde{c}\right)\langle E_{2}\rangle +c\tilde{c}\langle E_{3}\rangle \\ & = & \left(1-c\right)E^{\infty }-c\left(1-\tilde{c}\right){B|}_{\tilde{\epsilon_{3}}}+c\tilde{c}E_{3} \end{array}$$

where we have used the property $\langle E_{2}\rangle =-B$ above discussed. Here, *B* is not constant since it depends on ${\tilde{\epsilon }}_{3}$. We have (note that *D* and *B* have the same denominator)

(31) $$\begin{array}{c} -B=\frac{d\epsilon_{1}\left[\left(d-1\right)\epsilon_{2}+{\tilde{\epsilon }}_{3}\right]}{\alpha +\beta {\tilde{\epsilon }}_{3}}E^{\infty } \end{array}$$

From Equation (29) we can evaluate ${\tilde{\epsilon }}_{3}$ in terms of *α* and *β*

(32) $$\begin{array}{c} \alpha +\beta {\tilde{\epsilon }}_{3}=\frac{d^{2}\epsilon_{1}\epsilon_{2}}{E_{3}}E^{\infty }\Rightarrow {\tilde{\epsilon }}_{3}=\frac{1}{\beta }\left(\frac{d^{2}\epsilon_{1}\epsilon_{2}}{E_{3}}E^{\infty }-\alpha \right) \end{array}$$

Therefore, the coefficient -B assumes the simpler form

(33) $$\begin{array}{ccc} -B & = & \frac{d\epsilon_{1}\left[\left(d-1\right)\epsilon_{2}+\frac{1}{\beta }\left(\frac{d^{2}\epsilon_{1}\epsilon_{2}}{E_{3}}E^{\infty }-\alpha \right)\right]}{\frac{d^{2}\epsilon_{1}\epsilon_{2}}{E_{3}}E^{\infty }}E^{\infty }\\ & = & \frac{d\epsilon_{1}}{d^{2}\epsilon_{1}\epsilon_{2}}\left[\left(d-1\right)\epsilon_{2}+\frac{1}{\beta }\left(\frac{d^{2}\epsilon_{1}\epsilon_{2}}{E_{3}}E^{\infty }-\alpha \right)\right]E_{3}\\ & = & \frac{1}{d\epsilon_{2}}\left\{\left[\left(d-1\right)\epsilon_{2}-\frac{\alpha }{\beta }\right]E_{3}+\frac{d^{2}\epsilon_{1}\epsilon_{2}}{\beta }E^{\infty }\right\} \end{array}$$

Now, we can obtain $E^{\infty }$ in terms of $E_{3}$ from Equation (29)

(34) $$\begin{array}{c} E^{\infty }=\frac{\alpha +\beta \left(\epsilon_{3}+\chi {\left|E_{3}\right|}^{2}\right)}{d^{2}\epsilon_{1}\epsilon_{2}} \end{array}$$

Substituting the previous expression in Equation (33) we obtain the average value of the electric field in the coating shell

(35) $$\begin{array}{ccc} -B & = & \frac{1}{d\epsilon_{2}}\left[\left(d-1\right)\epsilon_{2}+\epsilon_{3}+\chi {\left|E_{3}\right|}^{2}\right]E_{3} \end{array}$$

The average value of the electric field over the entire structure is then obtained from Equation (30)

(36) $$\begin{array}{ccc} \langle E_{z}\rangle & = & \left(1-c\right)\frac{\alpha +\beta \left(\epsilon_{3}+\chi {\left|E_{3}\right|}^{2}\right)}{d^{2}\epsilon_{1}\epsilon_{2}}E_{3}\\ & + & c\left(1-\tilde{c}\right)\frac{1}{d\epsilon_{2}}\left[\left(d-1\right)\epsilon_{2}+\epsilon_{3}+\chi {\left|E_{3}\right|}^{2}\right]E_{3}+c\tilde{c}E_{3}\\ & = & \left\{\left(1-c\right)\frac{\alpha +\beta \epsilon_{3}}{d^{2}\epsilon_{1}\epsilon_{2}}+c\left(1-\tilde{c}\right)\frac{\left(d-1\right)\epsilon_{2}+\epsilon_{3}}{d\epsilon_{2}}+c\tilde{c}\right\}E_{3}\\ & + & \left\{\left(1-c\right)\frac{\beta }{d^{2}\epsilon_{1}\epsilon_{2}}+c\left(1-\tilde{c}\right)\frac{1}{d\epsilon_{2}}\right\}\chi {\left|E_{3}\right|}^{2}E_{3}\\ & = & \tilde{a}E_{3}+\tilde{b}{\left|E_{3}\right|}^{2}E_{3} \end{array}$$

where we have defined

(37) $$\begin{array}{ccc} \tilde{a} & = & \left\{\left(1-c\right)\frac{\alpha +\beta \epsilon_{3}}{d^{2}\epsilon_{1}\epsilon_{2}}+c\left(1-\tilde{c}\right)\frac{\left(d-1\right)\epsilon_{2}+\epsilon_{3}}{d\epsilon_{2}}+c\tilde{c}\right\}\\ \tilde{b} & = & \left\{\left(1-c\right)\frac{\beta }{d^{2}\epsilon_{1}\epsilon_{2}}+c\left(1-\tilde{c}\right)\frac{1}{d\epsilon_{2}}\right\}\chi \end{array}$$

Now, Equation (36) is in the form $\langle E_{z}\rangle =\tilde{a}E_{3}+\tilde{b}{\left|E_{3}\right|}^{2}E_{3}$, which is similar to Equation (12). It can be simply solved with respect to $E_{3}$ by considering the first two terms (of order one and three)

(38) $$\begin{array}{c} E_{3}=\frac{\langle E_{z}\rangle }{\tilde{a}}-\frac{\tilde{b}\langle E_{z}\rangle {\left|\langle E_{z}\rangle \right|}^{2}}{{\tilde{a}}^{2}{\left|\tilde{a}\right|}^{2}} \end{array}$$

We are searching for the effective dielectric constant and, therefore, for the relationship between the average electric field and the average electric displacement. Then, it is important to evaluate the average electric displacement (in direction *z*)

(39) $$\begin{array}{ccc} \langle D_{z}\rangle & = & \frac{1}{V}\int_{V}D_{z}\;dV\\ & = & \frac{1}{V}\int_{V_{1}}D_{1}\;dV+\frac{1}{V}\int_{V_{2}}D_{2}\;dV+\frac{1}{V}\int_{V_{3}}D_{3}\;dV\\ & & +\frac{\epsilon_{1}}{V}\int_{V_{2}}E_{2}\;dV-\frac{\epsilon_{1}}{V}\int_{V_{2}}E_{2}\;dV+\frac{\epsilon_{1}}{V}\int_{V_{3}}E_{3}\;dV-\frac{\epsilon_{1}}{V}\int_{V_{3}}E_{3}\;dV\\ & = & \epsilon_{1}\langle E_{z}\rangle +c\left(1-\tilde{c}\right)\left(\epsilon_{2}-\epsilon_{1}\right)\langle E_{2}\rangle +c\tilde{c}\langle \left({\tilde{\epsilon }}_{3}-\epsilon_{1}\right)E_{3}\rangle \\ & = & \epsilon_{1}\langle E_{z}\rangle +c\left(1-\tilde{c}\right)\left(\epsilon_{2}-\epsilon_{1}\right)\left({-B|}_{{\tilde{\epsilon }}_{3}}\right)+c\tilde{c}\left(\epsilon_{3}+\chi {\left|E_{3}\right|}^{2}-\epsilon_{1}\right)E_{3}\\ & = & \epsilon_{1}\langle E_{z}\rangle +c\left(1-\tilde{c}\right)\left(\epsilon_{2}-\epsilon_{1}\right)\frac{1}{d\epsilon_{2}}\left[\left(d-1\right)\epsilon_{2}+\epsilon_{3}+\chi {\left|E_{3}\right|}^{2}\right]E_{3}\\ & & +c\tilde{c}\left(\epsilon_{3}+\chi {\left|E_{3}\right|}^{2}-\epsilon_{1}\right)E_{3} \end{array}$$

where $D_{1}$, $D_{2}$ and $D_{3}$ are the displacement vectors in the three phases. We use Equation (38), giving the value of $E_{3}$, in order to approximate the quantity $|E_{3}{|}^{2}$. We obtain $|E_{3}{|}^{2}≅\frac{|\langle E_{z}\rangle {|}^{2}}{|\tilde{a}{|}^{2}}$. Similarly, we also obtain $E_{3}{\left|E_{3}\right|}^{2}≅\frac{\langle E_{z}\rangle }{\tilde{a}}\cdot \frac{|\langle E_{z}\rangle {|}^{2}}{|\tilde{a}{|}^{2}}$. Therefore, the average displacement field is given by

(40) $$\begin{array}{c} \langle D_{z}\rangle =\epsilon_{eff}\langle E_{z}\rangle +\chi_{eff}\langle E_{z}\rangle {\left|\langle E_{z}\rangle \right|}^{2} \end{array}$$

where we have defined the effective quantities

(41) $$\begin{array}{ccc} \epsilon_{eff} & = & \epsilon_{1}+c\left(1-\tilde{c}\right)\left(\epsilon_{2}-\epsilon_{1}\right)\frac{1}{d\epsilon_{2}}\left[\left(d-1\right)\epsilon_{2}+\epsilon_{3}\right]\frac{1}{\tilde{a}}+c\tilde{c}\left(\epsilon_{3}-\epsilon_{1}\right)\frac{1}{\tilde{a}} \end{array}$$

(42) $$\begin{array}{ccc} \chi_{eff} & = & c\left(1-\tilde{c}\right)\left(\epsilon_{2}-\epsilon_{1}\right)\frac{1}{d\epsilon_{2}}\left[\left(d-1\right)\epsilon_{2}+\epsilon_{3}\right]\frac{-\tilde{b}}{{\tilde{a}}^{2}{\left|\tilde{a}\right|}^{2}}\\ & & +c\left(1-\tilde{c}\right)\left(\epsilon_{2}-\epsilon_{1}\right)\frac{1}{d\epsilon_{2}}\frac{\chi }{\tilde{a}{\left|\tilde{a}\right|}^{2}}+c\tilde{c}\frac{\chi }{\tilde{a}{\left|\tilde{a}\right|}^{2}}-c\tilde{c}\left(\epsilon_{3}-\epsilon_{1}\right)\frac{\tilde{b}}{{\tilde{a}}^{2}{\left|\tilde{a}\right|}^{2}} \end{array}$$

The results given in Equations (41) and (42) represent the complete nonlinear characterization of a dilute dispersion of coated particles with nonlinear core. It is not difficult to verify that the result for $\epsilon_{eff}$ is coincident with Equation (25), as expected [39]. Moreover, the second relation for $\chi_{eff}$ represents a complementary result, describing the nonlinear behavior of the heterogeneous structure.

In this introductory section we have summed up the most important methodologies and results regarding simple geometries of heterogeneous structures. In the following we will use similar techniques to analyze more complicated microstructures.

## 3. Nonlinear Electric Homogenization for Pseudo-Oriented Ellipsoids

**Figure 2:**
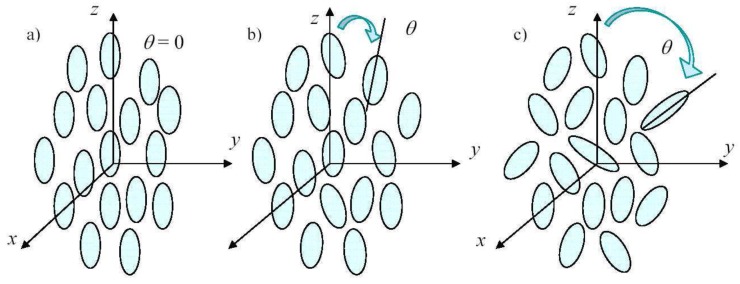
Structure of a dispersion of pseudo-oriented ellipsoids. One can find some orientational distributions ranging from order to disorder. The two-phase material is described by the electric response of each phase, by the state of order and by the volume fraction of the inclusions.

In recent material science development, considerable attention has been devoted to electromagnetically nonlinear composite structures due to their applications, for instance, to integrated optical devices (such as optical switching and signal processing devices) [51,52,53]. More specifically, intrinsic optical bistability has been extensively studied theoretically as well as experimentally with the help of mixture theory [54,55]. In all of these cases, a linear medium containing spherical or spheroidal inclusions has been considered. Important results concerning a dispersion of dielectrically nonlinear and graded parallel cylinders have been achieved by Wei *et al.* [56]. Our aim is to extend previous works and the techniques discussed in the previous section and to explore the importance of the orientational distribution and of the inclusion shape in this context. To do this, we consider a dispersion of dielectrically nonlinear spheroidal particles (ellipsoids of revolution), pseudo randomly oriented in a (dielectrically) linear matrix and we then develop a mathematical procedure to perform the needed averages of the electric quantities over all possible orientations of the inclusions. This analysis leads to the nonlinear anisotropic constitutive equation connecting the macroscopic electric displacement to the macroscopic electric field. A particular attention is devoted to the analysis of the effects of the orientational distribution of the particles inside the composite material. The limiting cases of the present theory are represented by all the particles aligned with a given direction (perfect order) and all the particles randomly oriented (complete disorder). We take into account all the intermediate configurations between order and disorder with the aim to characterize a material with particles partially aligned. In Figure 2 one can find some orientational distributions between the upon described limiting cases.

To define the geometry, we consider a given orthonormal reference frame and we take as preferential direction of alignment the *z*-axis. Each particle embedded in the matrix is not completely random oriented. The orientation is described by the following statistical rule: the principal axis of each particle forms with the *z*-axis an angle *ϑ*, which follows a given probability density $f_{\Theta }\left(\vartheta \right)$ symmetrically distributed in [0, *π*]. The symmetry of the density can be written as $f_{\Theta }\left(\vartheta \right)=f_{\Theta }\left(\pi -\vartheta \right)$. We assume that the orientation of each particle is statistically independent from the orientation of other particles. If $f_{\Theta }\left(\vartheta \right)=\left(1/2\right)\left(\delta \left(\vartheta \right)+\delta \left(\vartheta -\pi \right)\right)$ (where *δ* is the Dirac delta function) we have all the particles with *ϑ* = 0 (or ϑ=π, which corresponds to the same orientation) and, therefore, they are all oriented along the *z*-axis. If $f_{\Theta }\left(\vartheta \right)=\sin\left(\vartheta \right)/2$ all the particles are uniformly random oriented in the space over all the possible orientations. Any other symmetric statistical distribution $f_{\Theta }\left(\vartheta \right)$ defines a transversely isotropic (uniaxial) material with principal axis aligned with the *z*-axis. For example, if $f_{\Theta }\left(\vartheta \right)=\delta \left(\vartheta -\pi /2\right)$, all particles have the principal axis orthogonal to the *z*-axis. In the following sections we develop a complete analysis of the combined effects of the shape (aspect ratio or eccentricity) of the particles and their state of order/disorder. This analysis allows us to evaluate the overall electric properties of the heterogeneous material. In particular, from the point of view of the shape of the particles, the so-called depolarization factor *L* is the parameter that intervenes to characterize the medium. We verified that the state of order acts on the overall linear and nonlinear dielectric properties by means of two parameters that are defined as follows: $C_{2}=\langle \cos^{2}\vartheta \rangle$ and $C_{4}=\langle \cos^{4}\vartheta \rangle$. They correspond to the average values of $\cos^{2}\vartheta$ and $\cos^{4}\vartheta$, computed by means of the density probability $f_{\Theta }\left(\vartheta \right)$. The results may be applied to describe the physical behavior of heterogeneous materials starting from the knowledge of the physical properties of each medium composing the mixture as well as of the structural properties of the mixture itself, i.e., shape of the inclusions and state of order of the orientations (*L*, $C_{2}$ and $C_{4}$). It is worth pointing out, as it frequently occurs in this field, that the presented results have been derived under electrostatic assumption, but they hold valid also in the low frequency regime, as long as the wavelength is much larger than the largest dimension of the inclusions. The analysis performed in the following has immediate application to the field of the liquid crystals. Actually, our microstructure describes a material positionally disordered, but with partial orientational order, which corresponds to a nematic phase in liquid crystals [57,58]. The level of ordering is reflected in the macroscopic properties. Some previous works have been devoted to an analysis similar to that developed in this work but only from a dielectrically linear point of view [59,60,61,62]. So, the following development can be considered as a nonlinear extension of such previous ones.

## 4. Field Perturbation Due to One Single Nonlinear Ellipsoidal Inclusion in a Uniform Field

Here we present a general solution to the problem of a nonlinear ellipsoidal particle embedded in a linear material. The theory is based on the following result derived for the linear case, which describes the behavior of one electrically linear ellipsoidal particle of permittivity $\varepsilon_{2}$ in a linear homogeneous medium of permittivity $\varepsilon_{1}$. Let the axes of the ellipsoid be $l_{x}$, $l_{y}$ and $l_{z}$ (aligned with axes *x*, *y*, *z* of ellipsoid reference frame) and let a uniform electric field ${\vec{E}}_{0}=\left(E_{0x},E_{0y},E_{0z}\right)$ be applied to the structure. Then, according to Stratton [43], the electric field ${\vec{E}}_{s}=\left(E_{sx},E_{sy},E_{sz}\right)$ inside the ellipsoid is uniform and it can be expressed as follows

(43) Esi=E0i1+Liε2ε2ε1-1ε1-1

Here, and throughout the paper, the index *i* takes the *x*, *y* and *z* values. The expressions for the depolarization factors $L_{i}$ in the case of generally shaped ellipsoid can be found in the literature [17]. They can be expressed in terms of elliptic integrals. The condition $L_{x}+L_{y}+L_{z}=1$ is always fulfilled.

Let’s now generalize Equation (43) to the case where a dielectrically nonlinear ellipsoid is embedded in the linear matrix. A nonlinear isotropic and homogenous ellipsoid can be described from the electrical point of view by the constitutive equation $\vec{D}=\varepsilon \left(E\right)\vec{E}$ [63]. Here, $\vec{D}$ is the electric displacement inside the particle, $\vec{E}$ is the electric field and the function *ε* depends only on the modulus *E* of $\vec{E}$. This latter property accounts for the fact that the medium inside the ellipsoid is isotropic and homogenous. The main result follows. The electric field inside the inclusion is uniform even in the nonlinear case and it may be calculated by means of the following system of equations [27]

(44) Esi=E0i1+LiεEsεEsε1ε1-1,∀i

where, as before, ${\vec{E}}_{0}$ is a uniform electric field applied to the structure and ${\vec{E}}_{s}$, the unknown in the nonlinear system (44), is a uniform field as well. This property holds true due to the following reason: if a solution of (44) exists, due to self-consistency, all the boundary conditions are fulfilled and the problem is completely analogous to its linear counterpart, treated by Stratton [43], provided that $\varepsilon_{2}=\varepsilon \left(E_{s}\right)$.

In order to simplify the following analysis we will adopt ellipsoids of revolution. Thus we consider $l_{x}=l_{y}$ and we define the aspect ratio as $e=l_{z}$/$l_{x}=l_{z}$/$l_{y}$. The depolarization factors for ellipsoids of revolution may be computed in closed form as follows and the results depend on the shape of the ellipsoid [17]. It is prolate (of ovary or elongated form) if e>1 and oblate (of planetary or flattened form) if e<1

(45) $$\begin{array}{ccc} L_{x}=L_{y} & = & \frac{e}{2}\int_{0}^{+\infty }\frac{d\xi }{{\left(\xi +1\right)}^{2}{\left(\xi +e^{2}\right)}^{1/2}}=\left\{\begin{array}{c} \frac{e}{4p^{3}}\left[2ep+\ln\frac{e-p}{e+p}\right]\;if\;e>1\\ \frac{e}{4q^{3}}\left[\pi -2eq-2\arctan\frac{e}{q}\right]\;if\;e<1 \end{array}\right. \end{array}$$

(46) $$\begin{array}{ccc} L_{z} & = & \frac{e}{2}\int_{0}^{+\infty }\frac{d\xi }{\left(\xi +1\right){\left(\xi +e^{2}\right)}^{3/2}}=\left\{\begin{array}{c} \frac{1}{2p^{3}}\left[e\ln\frac{e+p}{e-p}-2p\right]\;if\;e>1\\ \frac{1}{2q^{3}}\left[2q-e\pi +2e\arctan\frac{e}{q}\right]\;if\;e<1 \end{array}\right. \end{array}$$

where $p=\sqrt{e^{2}-1}$ and $q=\sqrt{1-e^{2}}$. The relation 2$L_{x}+L_{z}$ = 1 holds on and therefore we will consider $L=L_{z}$ as main geometric parameter of the system. An interesting aspect related to the problem faced in this section shows up when one considers the nonlinear Equation (44) and tries to solve it iteratively [27]. This means that, in order to solve for $E_{s}$, one starts with a given initial value $E_{s}^{0}$, and one uses the successive approximations described by the iteration rule

(47) Esin+1=E0i1+LiεE→snεE→snε1ε1-1

The following sufficient convergence criterion has been verified [27]: the iteration rule given by Equation (47) is convergent to the exact internal electric field if the nonlinear material of the ellipsoid fulfils the condition $\left|\frac{E}{\varepsilon }\frac{\partial \varepsilon }{\partial E}\right|<1$. In a general context, one can describe isotropic nonlinear dielectric materials by means of the so-called Kerr nonlinearity relation, often adopted in metamaterials study

(48) $$\varepsilon \left(E\right)=\varepsilon_{2}+\alpha E^{2}$$

which assumes that $\varepsilon_{2}$ and *α* are constant. The Kerr nonlinearity is called *focusing* or *defocusing* according to the fact that α> 0 or α< 0, respectively [63]. It is straightforward to verify that the convergence condition $\left|\frac{E}{\varepsilon }\frac{\partial \varepsilon }{\partial E}\right|<1$ is always verified for *defocusing* Kerr nonlinearity and is verified only if $E_{s}^{2}<\varepsilon_{2}/\alpha$ (here $E_{s}$ is the modulus of the actual electric field inside the inclusion) in the case of *focusing* nonlinearity [27].

## 5. Average Electric Field Inside a Single Pseudo-Random Oriented Inclusion

Now, our aim is to find an explicit version of Equation (44), which is valid when the nonlinear permittivity is given by Equation (48). To begin the analysis, we substitute Equation (48), holding for a single ellipsoid, in Equation (44)

(49) $$E_{si}=\frac{\varepsilon_{1}E_{0i}}{\varepsilon_{1}+L_{i}\left[\varepsilon_{2}-\varepsilon_{1}+\alpha \left(E_{sx}^{2}+E_{sy}^{2}+E_{sz}^{2}\right)\right]}$$

This is an algebraic system of degree nine with three unknowns, namely $E_{sx},E_{sy}$, and $E_{sz}$. It might be hard, if not impossible, to be solved analytically, but we are interested, for our purposes, in just the first terms of a series expansion for the solution. To obtain it, we may adopt the *ansatz* $E_{si}=k_{i}E_{0i}+h_{i}E_{0i}^{3}$ and solve for $k_{i}\;\mathrm{and}\;h_{i}$. Alternatively, we may use the iterative scheme given in Equation (47), in literal form, adopting only the first iterations. For sake of brevity, we omit here the simple but long calculation, which leads to the solution

(50) $$\begin{array}{ccc} E_{si} & = & \frac{\varepsilon_{1}E_{0i}}{\left(1-L_{i}\right)\varepsilon_{1}+L_{i}\varepsilon_{2}}-\frac{\alpha \varepsilon_{1}^{3}L_{i}E_{0i}}{{\left[\left(1-L_{i}\right)\varepsilon_{1}+L_{i}\varepsilon_{2}\right]}^{2}}\sum_{j}\frac{E_{0j}^{2}}{{\left[\left(1-L_{j}\right)\varepsilon_{1}+L_{j}\varepsilon_{2}\right]}^{2}}+... \end{array}$$

We observe that the first term represents the classical Lorentz field appearing in a dielectrically linear ellipsoidal inclusion. The second term is the first nonlinear contribution, which is directly proportional to the inclusion hyper-susceptibility *α*. To simplify the expressions, from now on, we will use the notation: $a_{i}=\left(1-L_{i}\right)\varepsilon_{1}+L_{i}\varepsilon_{2}$. To derive the mixture behavior, we need to calculate the electric field in a single nonlinear ellipsoidal inclusion arbitrarily oriented in space and embedded in a homogeneous medium with permittivity $\varepsilon_{1}$. In order to do this, we shall express Equation (50) in the global framework of reference of the mixture. We define three unit vectors, indicating the principal directions of each ellipsoid in space: they are referred to as ${\hat{n}}_{x},{\hat{n}}_{y}\;\mathrm{and}\;{\hat{n}}_{z}$, and they correspond to the axes of the ellipsoid. By using Equation (50), we may compute the electric field induced by a given external arbitrary uniform electric field inside the inclusion (from now on we will omit the additional higher order terms)

(51) $${\vec{E}}_{s}=\left[\frac{\varepsilon_{1}{\vec{E}}_{0}\cdot {\hat{n}}_{i}}{a_{i}}-\frac{\alpha \varepsilon_{1}^{3}L_{i}{\vec{E}}_{0}\cdot {\hat{n}}_{i}}{a_{i}^{2}}\frac{\left({\vec{E}}_{0}\cdot {\hat{n}}_{j}\right)\left({\vec{E}}_{0}\cdot {\hat{n}}_{j}\right)}{a_{j}^{2}}\right]{\hat{n}}_{i}$$

We shall now average it over all the possible orientations of the particle. The expression for the internal electric field in Equation (51) can be rewritten component by component as follows

(52) $$E_{sk}=\left[\frac{\varepsilon_{1}E_{0l}n_{il}}{a_{i}}-\frac{\alpha \varepsilon_{1}^{3}L_{i}E_{0l}n_{il}}{a_{i}^{2}}\frac{E_{0q}n_{jq}E_{0p}n_{jp}}{a_{j}^{2}}\right]n_{ik}$$

where $n_{jk}$ is the *k*-th component of the unit vector ${\hat{n}}_{j}$, (j=x, *y*, *z*) and we have considered the implicit sums of *i*, *j*, *l*, *q* and *p*over 1, 2 and 3. For the following derivation, we are interested in the average value of the electric field ${\vec{E}}_{s}$ over all the possible orientations of the ellipsoid itself and then we have to compute the following

(53) $$\langle E_{sk}\rangle =\frac{\varepsilon_{1}E_{0l}\langle n_{il}n_{ik}\rangle }{a_{i}}-\frac{\alpha \varepsilon_{1}^{3}L_{i}E_{0l}E_{0q}E_{0p}\langle n_{ik}n_{il}n_{jq}n_{jp}\rangle }{a_{i}^{2}a_{j}^{2}}$$

We may use Euler angles representation (ψ,φ and ϑ) to write down the explicit expressions for the components of the unit vectors ${\hat{n}}_{x},{\hat{n}}_{y}\;\mathrm{and}\;{\hat{n}}_{z}$

(54) $$\left\{\begin{array}{c} {\hat{n}}_{x}=\left(\cos\psi \cos\varphi -\sin\psi \sin\varphi \cos\vartheta ,-\cos\psi \sin\varphi -\sin\psi \cos\varphi \cos\vartheta ,\sin\psi \sin\vartheta \right)\\ {\hat{n}}_{y}=\left(\sin\psi \cos\varphi +\cos\psi \sin\varphi \cos\vartheta ,-\sin\psi \sin\varphi +\cos\psi \cos\varphi \cos\vartheta ,-\cos\psi \sin\vartheta \right)\\ {\hat{n}}_{z}=\left(\sin\varphi \sin\vartheta ,\sin\vartheta \cos\varphi ,\cos\vartheta \right) \end{array}\right.$$

In order to obtain the average value of the electric field ${\vec{E}}_{s}$, we need to calculate the average value of the quantities defined in Equation (53). This is done by the following integral over the Euler angles

(55) $$\langle E_{sk}\rangle =\frac{1}{4\pi^{2}}\int_{0}^{\pi }\int_{0}^{2\pi }\int_{0}^{2\pi }\left[\frac{\varepsilon_{1}E_{0l}n_{il}n_{ik}}{a_{i}}-\frac{\alpha \varepsilon_{1}^{3}L_{i}E_{0l}E_{0q}E_{0p}n_{ik}n_{il}n_{jq}n_{jp}}{a_{i}^{2}a_{j}^{2}}\right]d\varphi \;d\psi \;f_{\Theta }\left(\vartheta \right)d\vartheta$$

In order to represent the above described orientation of the particles, the angles *φ* and *ψ* are uniformly distributed over the entire range [0 2*π*] and the angle *ϑ* follows the given probability density $f_{\Theta }\left(\vartheta \right)$ over the range [0 *π*]. So, by performing the integration described in Equation (55) and by using Equation (54), we may obtain that the average value of $E_{sk}$ depends on the two following parameters, defined by means of the density probability $f_{\Theta }\left(\vartheta \right)$

(56) $$C_{2}=\int_{0}^{\pi }\cos^{2}\vartheta f_{\Theta }\left(\vartheta \right)d\vartheta \;\;\;\;\;\;\;\;\mathrm{and}\;\;\;\;\;\;\;\;C_{4}=\int_{0}^{\pi }\cos^{4}\vartheta f_{\Theta }\left(\vartheta \right)d\vartheta$$

These two parameters completely characterize the effects of the pseudo-orientation of the particles inside the medium. Some particular values follow. When we are in the case of perfect order we have $f_{\Theta }\left(\vartheta \right)=\left(1/2\right)\left(\delta \left(\vartheta \right)+\delta \left(\vartheta -\pi \right)\right)$ and the corresponding values are $C_{2}=1$ and $C_{4}=1$. If we are in the case of complete disorder we have $f_{\Theta }\left(\vartheta \right)=\sin\left(\vartheta \right)/2$ and we obtain $C_{2}=1/3$ and $C_{4}=1/5$. Finally, when the particles all have the principal axis orthogonal to the *z*-axis we have $f_{\Theta }\left(\vartheta \right)=\delta \left(\vartheta -\pi /2\right)$ and the values of the parameters are $C_{2}=0$ and $C_{4}=0$.

Since we are dealing with ellipsoids of revolution, in performing the integration of Equation (55) we use the simplified notation $a_{1}=a_{2}$ and $L_{1}=L_{2}=\left(1-L\right)/2$, $L_{3}=L$. The factor *L* assumes some characteristic values in correspondence to special shapes of the particles: for spheres L=1/3, for cylinders L=0 and for lamellae or penny shaped inclusions L=1. Summing up, we verified, after a very long by straightforward integration, that the following simple relation gives the final result of the averaging process

(57) $$\langle E_{sk}\rangle =\gamma_{k}E_{0k}-\alpha \mu_{kl}E_{0k}E_{0l}^{2}$$

Here, the sum on the index *l* is implied and the parameters $\gamma_{k}$ and $\mu_{kl}$ can be organized as follows

(58) $$\gamma =\left[\begin{array}{c} \gamma_{1}\\ \gamma_{1}\\ \gamma_{3} \end{array}\right];\;\;\mu =\left[\begin{array}{ccc} \mu_{11} & \mu_{11} & \mu_{13}\\ \mu_{11} & \mu_{11} & \mu_{13}\\ \mu_{31} & \mu_{31} & \mu_{33} \end{array}\right]$$

The explicit results for the parameters $\gamma_{k}$ are

(59) $$\gamma_{1}=\frac{1}{2}\varepsilon_{1}\frac{a_{1}+a_{3}}{a_{1}a_{3}}+\frac{1}{2}\varepsilon_{1}\frac{a_{3}-a_{1}}{a_{1}a_{3}}C_{2}$$

(60) $$\gamma_{3}=\varepsilon_{1}\frac{1}{a_{1}}+\varepsilon_{1}\frac{a_{1}-a_{3}}{a_{1}a_{3}}C_{2}$$

Moreover, the explicit results for the parameters $\mu_{kl}$ are

(61) $$\mu_{11}=\frac{\varepsilon_{1}^{3}}{16a_{1}^{4}a_{3}^{4}}\left\{\begin{array}{c} 3a_{3}^{4}\left(1-L\right)+6La_{1}^{4}+C_{2}\left[2a_{1}^{2}a_{3}^{2}\left(1+L\right)+2a_{3}^{4}\left(1-L\right)-12La_{1}^{4}\right]+\\ a_{1}^{2}a_{3}^{2}\left(1+L\right)+C_{4}\left[6La_{1}^{4}-3a_{1}^{2}a_{3}^{2}\left(1+L\right)+3a_{3}^{4}\left(1-L\right)\right] \end{array}\right\}$$

(62) $$\mu_{13}=\frac{\varepsilon_{1}^{3}}{16a_{1}^{4}a_{3}^{4}}\left\{\begin{array}{c} 8La_{1}^{2}a_{3}^{2}+4a_{3}^{4}\left(1-L\right)+C_{2}\left[8a_{3}^{4}\left(1-L\right)+24La_{1}^{4}-4a_{1}^{2}a_{3}^{2}\left(1+7L\right)\right]+\\ +C_{4}\left[12a_{1}^{2}a_{3}^{2}\left(1+L\right)-12a_{3}^{4}\left(1-L\right)-24La_{1}^{4}\right] \end{array}\right\}$$

(63) $$\mu_{31}=\frac{\varepsilon_{1}^{3}}{4a_{1}^{4}a_{3}^{4}}\left\{\begin{array}{c} a_{1}^{2}a_{3}^{2}\left(1-L\right)+a_{3}^{4}\left(1-L\right)+C_{2}\left[2a_{3}^{4}\left(1-L\right)+6La_{1}^{4}+2a_{1}^{2}a_{3}^{2}\left(L-2\right)\right]+\\ +C_{4}\left[3a_{1}^{2}a_{3}^{2}\left(1+L\right)-3a_{3}^{4}\left(1-L\right)-6La_{1}^{4}\right] \end{array}\right\}$$

(64) $$\mu_{33}=\frac{\varepsilon_{1}^{3}}{4a_{1}^{4}a_{3}^{4}}\left\{\begin{array}{c} 2a_{3}^{4}\left(1-L\right)+C_{2}\left[2a_{1}^{2}a_{3}^{2}\left(1+L\right)-4a_{3}^{4}\left(1-L\right)\right]+\\ +C_{4}\left[2a_{3}^{4}\left(1-L\right)-2a_{1}^{2}a_{3}^{2}\left(1+L\right)+4La_{1}^{4}\right] \end{array}\right\}$$

This is a first analytical result, which will play a crucial role in the following development of the theory. It is interesting to observe that if $a_{1}=a_{3}$ and L=1/3 (we are dealing with spherical inclusions), then the terms containing $C_{2}$ and $C_{4}$ completely disappear in Equations (59)–(64): it is correct since the orientation is not important for an isotropic spherical object.

## 6. Averaging Process in a Dilute Mixture

From now on we analyze the dispersion of pseudo-oriented nonlinear ellipsoids. The permittivity of the inclusions is described by the isotropic nonlinear relation $\varepsilon \left(E\right)=\varepsilon_{2}+\alpha E^{2}$ [see Equation (48)] and the linear matrix has permittivity $\varepsilon_{1}$; the overall electrical behavior of the dispersion is expected to be anisotropic because of the pseudo-random orientation of the particles. This is true because the *z*-axis has a special character induced by the partial alignment of the particles. Therefore, the equivalent electric constitutive equation can be expanded in series with respect to the averaged electric field components: $\langle D_{k}\rangle =\varepsilon_{kj}^{eq}\langle E_{j}\rangle +\chi_{kjil}^{eq}\langle E_{j}\rangle \langle E_{i}\rangle \langle E_{l}\rangle +...$, where the coefficients $\varepsilon^{eq}$ (the superscript *eq* pointing out the *equivalent* character of the term) and $\chi^{eq}$ are tensors depending on various parameters of the mixture such as the aspect ratio *e* of the ellipsoids, the volume fraction *c* of the included phase, the density probability $f_{\Theta }\left(\vartheta \right)$ describing the orientational distribution, the permittivities $\varepsilon_{1}$, $\varepsilon_{2}$ and the Kerr susceptibility *α* of the inclusions. The homogenization procedure should provide the structure of the entries of the tensors $\varepsilon^{eq}$ and $\chi^{eq}$ in terms of the mentioned parameters. In the technical literature, the coefficients *α* and $\chi^{eq}$ (the first nonlinear terms of the expanded constitutive equations for inclusions and mixture, respectively) are often called hyper-susceptibilities [27].

The main achievement of this work is the derivation of a closed form expression for the hyper-susceptibility ratio $\chi^{eq}/\alpha$. These quantities are of interest as much as they represent the amplification of the composite material nonlinear behavior with respect to that of the inclusions. In particular we are interested in the dependence of these parameters on the state of order/disorder of the system, which is well described by the density probability $f_{\Theta }\left(\vartheta \right)$. In other words, it means that we will write our results in terms of the order parameters $C_{2}$ and $C_{4}$. Moreover, we may describe the dependence of the hyper-susceptibility ratio $\chi^{eq}/\alpha$ on the aspect ratio of the embedded particles, i.e., on the parameter *e* or *L*. The final expressions are derived under the assumption that the constitutive equation of the composite medium is of the form $\langle D_{k}\rangle =\varepsilon_{kj}^{eq}\langle E_{j}\rangle +\chi_{kjil}^{eq}\langle E_{j}\rangle \langle E_{i}\rangle \langle E_{l}\rangle$, which neglects higher order terms. All the computations are carried out under the same hypothesis underlying the linear Maxwell-Garnett theory, that is, low concentration *c* of the dispersed phase. So, if we consider a mixture with a volume fraction c<<1 of pseudo-randomly oriented, dielectrically nonlinear ellipsoids embedded in a homogeneous matrix with permittivity $\varepsilon_{1}$, we can evaluate the average of the electric field over the space occupied by the mixture. It can be done via the following relationship

(65) $$\langle \vec{E}\rangle =c\langle {\vec{E}}_{s}\rangle +\left(1-c\right){\vec{E}}_{0}$$

This means that we do not take into account the interactions among the inclusions because of the very low concentration: each ellipsoid behaves as an isolated one. Once more, to derive Equation (65), we assume an approximately uniform average electric field ${\vec{E}}_{0}$ in the space outside the inclusions. To evaluate the equivalent constitutive equation, we compute the average value of the displacement vector inside the random material. The region *V* is defined as the space occupied by the mixture, *Ve* as the region occupied by the inclusions, and *Vo* as the remaining space (so that *V* = *Ve*∪*Vo*). The average value of $\vec{D}\left(\vec{r}\right)=\varepsilon \vec{E}\left(\vec{r}\right)$ is evaluated as follows ($\vec{D}\;\mathrm{and}\;\vec{E}$ represent the local fields, $\langle \vec{D}\rangle \;\mathrm{and}\;\langle \vec{E}\rangle$ their macroscopic counterparts)

(66) $$\begin{array}{ccc} \langle \vec{D}\rangle & = & \frac{1}{\left|V\right|}\int_{V}\varepsilon \vec{E}\left(\vec{r}\right)d\vec{r}=\frac{1}{\left|V\right|}\varepsilon_{1}\int_{V_{o}}\vec{E}\left(\vec{r}\right)d\vec{r}+\frac{1}{\left|V\right|}\int_{V_{e}}\varepsilon \vec{E}\left(\vec{r}\right)d\vec{r}\\ & = & \frac{1}{\left|V\right|}\varepsilon_{1}\int_{V_{o}}\vec{E}\left(\vec{r}\right)d\vec{r}+\frac{1}{\left|V\right|}\varepsilon_{1}\int_{V_{e}}\vec{E}\left(\vec{r}\right)d\vec{r}+\frac{1}{\left|V\right|}\frac{\left|V_{e}\right|}{\left|V_{e}\right|}\int_{V_{e}}\left(\varepsilon -\varepsilon_{1}\right)\vec{E}\left(\vec{r}\right)d\vec{r}\\ & = & \varepsilon_{1}\langle \vec{E}\rangle +c\langle \left[\varepsilon \left(E_{s}\right)-\varepsilon_{1}\right]{\vec{E}}_{s}\rangle \end{array}$$

Here $\left|V\right|$is the measure of the region *V*. It can be noted that the average value given by the expression $\langle \left[\varepsilon \left(∥{\vec{E}}_{s}∥\right)-\varepsilon_{1}\right]{\vec{E}}_{s}\rangle$ is not available from the previous computations. We consider a single ellipsoidal nonlinear inclusion and we search for the average value of the quantity $\left[\varepsilon \left(∥{\vec{E}}_{s}∥\right)-\varepsilon_{1}\right]{\vec{E}}_{s}$ over all the possible orientations of the particle. From Equations (44) and (50) we obtain

(67) $$\left[\varepsilon \left(∥{\vec{E}}_{s}∥\right)-\varepsilon_{1}\right]E_{si}=\frac{\varepsilon_{1}}{L_{i}}\left(E_{0i}-E_{si}\right)=\frac{\varepsilon_{1}}{L_{i}}\left[E_{0i}-\frac{\varepsilon_{1}E_{0i}}{a_{i}}+\frac{\alpha \varepsilon_{1}^{3}L_{i}E_{0i}}{a_{i}^{2}}\sum_{j}\frac{E_{0j}^{2}}{a_{j}^{2}}\right]$$

therefore, in vector notation

(68) $$\left[\varepsilon \left(∥{\vec{E}}_{s}∥\right)-\varepsilon_{1}\right]{\vec{E}}_{s}=\frac{\varepsilon_{1}}{L_{i}}\left[{\vec{E}}_{0}\cdot {\hat{n}}_{i}-\frac{\varepsilon_{1}{\vec{E}}_{0}\cdot {\hat{n}}_{i}}{a_{i}}+\frac{\alpha \varepsilon_{1}^{3}L_{i}{\vec{E}}_{0}\cdot {\hat{n}}_{i}}{a_{i}^{2}}\frac{\left({\vec{E}}_{0}\cdot {\hat{n}}_{j}\right)\left({\vec{E}}_{0}\cdot {\hat{n}}_{j}\right)}{a_{j}^{2}}\right]\;{\hat{n}}_{i}$$

By taking the *k*-th component in the global reference framework, we may write

(69) $$\left[\varepsilon \left(∥{\vec{E}}_{s}∥\right)-\varepsilon_{1}\right]E_{sk}=\frac{\varepsilon_{1}}{L_{i}}\left[E_{0l}n_{il}-\frac{\varepsilon_{1}E_{0l}n_{il}}{a_{i}}+\frac{\alpha \varepsilon_{1}^{3}L_{i}E_{0l}n_{il}}{a_{i}^{2}}\frac{E_{0q}n_{jq}E_{0p}n_{jp}}{a_{j}^{2}}\right]n_{ik}$$

and averaging, after some straightforward computation

(70) $$\langle D_{sk}-\varepsilon_{1}E_{sk}\rangle =\frac{\varepsilon_{1}\left(\varepsilon_{2}-\varepsilon_{1}\right)}{a_{i}}E_{0l}\langle n_{il}n_{ik}\rangle +\frac{\alpha \varepsilon_{1}^{4}E_{0l}}{a_{i}^{2}}\frac{E_{0q}E_{0p}}{a_{j}^{2}}\langle n_{il}n_{ik}n_{jq}n_{jp}\rangle$$

At this point, the explicit average value can be found by taking into consideration the expressions of the unit vectors ${\hat{n}}_{x},{\hat{n}}_{y}\;\mathrm{and}\;{\hat{n}}_{z}$, given in Equation (54), and performing the integration in a similar way to that shown in Equation (55). The effects of the pseudo-orientation of the particles inside the medium are described, as before, by the order parameters $C_{2}$ and $C_{4}$. Of course, performing the averaging in Equation (70) we have used again the simplified notation $a_{1}=a_{2}$ and $L_{1}=L_{2}=\left(1-L\right)/2$, $L_{3}=L$. So, the final result for the quantity $\langle D_{sk}-\varepsilon_{1}E_{sk}\rangle$ is given by the following simple relation

(71) $$\langle D_{sk}-\varepsilon_{1}E_{sk}\rangle =\left(\varepsilon_{2}-\varepsilon_{1}\right)\gamma_{k}E_{0k}+\alpha \lambda_{kl}E_{0k}E_{0l}^{2}$$

Here the sum on the index *l* is implied and the parameters $\gamma_{k}$ have been defined in Equations (58), (59) and (60). Moreover, the parameters $\lambda_{kl}$ can be arranged in following matrix notation

(72) $$\lambda =\left[\begin{array}{ccc} \lambda_{11} & \lambda_{11} & \lambda_{13}\\ \lambda_{11} & \lambda_{11} & \lambda_{13}\\ \lambda_{13} & \lambda_{13} & \lambda_{33} \end{array}\right]$$

The explicit values of the relative entries have been calculated as follows

(73) $$\lambda_{11}=\frac{\varepsilon_{1}^{4}}{8a_{1}^{4}a_{3}^{4}}\left\{3a_{1}^{4}+2a_{1}^{2}a_{3}^{2}+3a_{3}^{4}+C_{2}\left[4a_{1}^{2}a_{3}^{2}+2a_{3}^{4}-6a_{1}^{4}\right]+C_{4}\left[3a_{1}^{4}-6a_{1}^{2}a_{3}^{2}+3a_{3}^{4}\right]\right\}$$

(74) $$\lambda_{13}=\frac{\varepsilon_{1}^{4}}{8a_{1}^{4}a_{3}^{4}}\left\{4a_{1}^{2}a_{3}^{2}+4a_{3}^{4}+C_{2}\left[8a_{3}^{4}+12a_{1}^{4}-20a_{1}^{2}a_{3}^{2}\right]+C_{4}\left[24a_{1}^{2}a_{3}^{2}-12a_{3}^{4}-12a_{1}^{4}\right]\right\}$$

(75) $$\lambda_{33}=\frac{\varepsilon_{1}^{4}}{2a_{1}^{4}a_{3}^{4}}\left\{2a_{3}^{4}+C_{2}\left[4a_{1}^{2}a_{3}^{2}-4a_{3}^{4}\right]+C_{4}\left[2a_{3}^{4}+2a_{1}^{4}-4a_{1}^{2}a_{3}^{2}\right]\right\}$$

Once again, we observe that if $a_{1}=a_{3}$ (we are dealing with spherical inclusions), then the terms containing $C_{2}$ and $C_{4}$ completely disappear in Equations (73)–(75): the orientation is not relevant for an isotropic spherical object. At this point we have all the balance equations needed to describe the overall electrical behavior of the pseudo random dispersion. These relationships have been summarized in Equation (76). The first relation corresponds to Equation (65) and furnishes the average electric field over the mixture volume in terms of the applied field and the average internal field (equation deduced under the hypothesis of low concentration). The second relation is taken from Equation (57) and gives the explicit value of the average electric field inside an inclusion (it accounts just for the first nonlinear terms). The third relation [see Equation (66)] furnishes the average value of the displacement vector over the entire mixture volume (this formula is exact). Finally, the fourth equation is taken from Equation (71) and gives the average value of the quantities $\langle D_{sk}-\varepsilon_{1}E_{sk}\rangle$ in terms of the applied electric field (as before, it accounts just for the first nonlinear terms). The complete set of the balance equations follows

(76) $$\left\{\begin{array}{c} \langle E_{k}\rangle =c\langle E_{sk}\rangle +\left(1-c\right)E_{0k}\\ \langle E_{sk}\rangle =\gamma_{k}E_{0k}-\alpha \mu_{kl}E_{0k}E_{0l}^{2}\\ \langle D_{k}\rangle =\varepsilon_{1}\langle E_{k}\rangle +c\langle D_{sk}-\varepsilon_{1}E_{sk}\rangle \\ \langle D_{sk}-\varepsilon_{1}E_{sk}\rangle =\left(\varepsilon_{2}-\varepsilon_{1}\right)\gamma_{k}E_{0k}+\alpha \lambda_{kl}E_{0k}E_{0l}^{2} \end{array}\right.$$

We may observe that the nonlinear terms, appearing in the second and in the fourth equations, are simply proportional to the hypersusceptibility *α* of the inclusions. By substituting the second equation in the first one and the fourth relation in the third one we obtain a simpler system

(77) $$\left\{\begin{array}{c} \langle E_{k}\rangle =\left(1-c+c\gamma_{k}\right)E_{0k}-c\alpha \mu_{kl}E_{0k}E_{0l}^{2}\\ \langle D_{k}\rangle =\varepsilon_{1}\langle E_{k}\rangle +c\left(\varepsilon_{2}-\varepsilon_{1}\right)\gamma_{k}E_{0k}+c\alpha \lambda_{kl}E_{0k}E_{0l}^{2} \end{array}\right.$$

Here, we have three vector fields involved: the average electric field over the entire mixture, the average electric displacement and the external applied electric field. In order to find out the effective constitutive equation for the whole composite material, we should obtain a relationship among the components $\langle D_{k}\rangle$ and the components $\langle E_{h}\rangle$. So we have to eliminate the external field $E_{0k}$ in Equation (77). Therefore, now we need to solve the first relation in Equation (77) with respect to $E_{0k}$: for our purposes it is sufficient to obtain a series solution with two terms and thus we let $E_{0k}=g_{k}\langle E_{k}\rangle +m_{kl}\langle E_{k}\rangle {\langle E_{l}\rangle }^{2}$, we substitute it in the first relation in Equation (77) and we solve for the unknown coefficients $g_{k}$ and $m_{kl}$. The result is

(78) $$E_{0k}=\frac{\langle E_{k}\rangle }{1-c+c\gamma_{k}}+\frac{c\alpha \mu_{kl}\langle E_{k}\rangle {\langle E_{l}\rangle }^{2}}{{\left(1-c+c\gamma_{k}\right)}^{2}{\left(1-c+c\gamma_{l}\right)}^{2}}$$

The final achievement is obtained by substituting Equation (78) in the second equation of system (77) and neglecting the powers of $\langle E_{k}\rangle$ greater than three

(79) $$\langle D_{k}\rangle =\left[\varepsilon_{1}+c\left(\varepsilon_{2}-\varepsilon_{1}\right)\frac{\gamma_{k}}{1-c+c\gamma_{k}}\right]\langle E_{k}\rangle +\left[\alpha \frac{c^{2}\left(\varepsilon_{2}-\varepsilon_{1}\right)\gamma_{k}\mu_{kl}+c\lambda_{kl}\left(1-c+c\gamma_{k}\right)}{{\left(1-c+c\gamma_{k}\right)}^{2}{\left(1-c+c\gamma_{l}\right)}^{2}}\right]\langle E_{k}\rangle {\langle E_{l}\rangle }^{2}$$

This is the first form of the constitutive equation of the overall dispersion. This result can be further simplified by defining the following quantities, which better describes the transversely isotropic (uniaxial) character of the composite material: $E_{⊥}^{2}={\langle E_{1}\rangle }^{2}+{\langle E_{2}\rangle }^{2}\;$, $E_{//}=\langle E_{3}\rangle$, $D_{⊥}^{2}={\langle D_{1}\rangle }^{2}+{\langle D_{2}\rangle }^{2}$ and $D_{//}=\langle D_{3}\rangle$. The symbol // indicates the components aligned with the principal axis (the *z*-axis) and the symbol *⊥* indicates the components orthogonal to the principal axis. With such conventions Equation (79) may be rearranged as follows

(80) $$\left\{\begin{array}{c} D_{⊥}=E_{⊥}\left[\varepsilon_{⊥}+\chi_{⊥,⊥}E_{⊥}^{2}+\chi_{⊥,//}E_{//}^{2}\right]\\ D_{//}=E_{//}\left[\varepsilon_{//}+\chi_{//,⊥}E_{⊥}^{2}+\chi_{//,//}E_{//}^{2}\right] \end{array}\right.$$

The linear permittivities $\varepsilon_{⊥}\;\text{and}\;\varepsilon_{//}$ and the nonlinear hyper-susceptibilities $\chi_{⊥,⊥},\;\chi_{⊥,//},\chi_{//,⊥}\;\text{and}\;\chi_{//,//}$ can be derived by comparison with Equation (79) and the relative explicit expressions are given below

(81) $$\left\{\begin{array}{c} \varepsilon_{⊥}=\varepsilon_{1}+c\left(\varepsilon_{2}-\varepsilon_{1}\right)\frac{\gamma_{1}}{1-c+c\gamma_{1}}\\ \varepsilon_{//}=\varepsilon_{1}+c\left(\varepsilon_{2}-\varepsilon_{1}\right)\frac{\gamma_{3}}{1-c+c\gamma_{3}} \end{array}\right.\;\;\left\{\begin{array}{c} \chi_{⊥,⊥}=\alpha \frac{c^{2}\left(\varepsilon_{2}-\varepsilon_{1}\right)\gamma_{1}\mu_{11}+c\lambda_{11}\left(1-c+c\gamma_{1}\right)}{{\left(1-c+c\gamma_{1}\right)}^{4}}\\ \chi_{⊥,//}=\alpha \frac{c^{2}\left(\varepsilon_{2}-\varepsilon_{1}\right)\gamma_{1}\mu_{13}+c\lambda_{13}\left(1-c+c\gamma_{1}\right)}{{\left(1-c+c\gamma_{1}\right)}^{2}{\left(1-c+c\gamma_{3}\right)}^{2}}\\ \chi_{//,⊥}=\alpha \frac{c^{2}\left(\varepsilon_{2}-\varepsilon_{1}\right)\gamma_{3}\mu_{31}+c\lambda_{13}\left(1-c+c\gamma_{3}\right)}{{\left(1-c+c\gamma_{1}\right)}^{2}{\left(1-c+c\gamma_{3}\right)}^{2}}\\ \chi_{//,//}=\alpha \frac{c^{2}\left(\varepsilon_{2}-\varepsilon_{1}\right)\gamma_{3}\mu_{33}+c\lambda_{33}\left(1-c+c\gamma_{3}\right)}{{\left(1-c+c\gamma_{3}\right)}^{4}} \end{array}\right.$$

All these quantities are the main parameters describing the nonlinear electrical behavior of the overall dispersion. We may observe that the nonlinear susceptibilities $\chi_{⊥,⊥},\;\chi_{⊥,//},\chi_{//,⊥}\;\text{and}\;\chi_{//,//}$ are proportional to the susceptibility *α* of the inclusions and depend on the factors $\gamma_{k},\;\mu_{kl}\;\text{and}\;\lambda_{kl}$ defined in Equations (59)–(64) and Equations (73)–(75). It is worth pointing out that, as it is expected, the results are explicitly written in terms of the depolarizing factor $L_{z}=L$ of the inclusions, which directly depends on the aspect ratio *e* [see Equation (45)], and in terms of the order parameters $C_{2}$ and $C_{4}$ that define the state of orientational order/disorder, which depends on the probability density $f_{\Theta }\left(\vartheta \right)$ (see Equation (56)). Finally, the particular cases of spherical inclusions ($a_{1}=a_{3}$ and *L* = 1/3) and of ellipsoidal inclusions with isotropic distribution ($C_{2}$ = 1/3 and $C_{4}$ = 1/5) provide results in perfect agreement with previous investigations [27].

## 7. Example of Application

In order to show some results of the previous procedure we choose a particular probability density $f_{\Theta }\left(\vartheta \right)$ that depends on one parameter *a*. This probability density is particularly useful because when the parameter *a* varies from -∞ to +∞ the orientational distribution of the inclusions assumes all the interesting possibilities. More precisely, when a→-∞ we are in the case of perfect order and all the particles are aligned with the *z*-axis, when *a* = 0 we are in the case of complete disorder (all the particles uniformly random oriented in the space) and when a→+∞ all the inclusions have the principal axis orthogonal to the *z*-axis. The expression of the normalized probability density over the range [0, *π*] follows

(82) $$f_{\Theta }\left(\vartheta \right)=\left\{\begin{array}{c} \frac{1}{2}\sin\left(\vartheta \right)\frac{\left(a^{2}+1\right)\;e^{a\vartheta }}{ae^{a\frac{\pi }{2}}+1}\;\;\;if\;0\leq \vartheta \leq \frac{\pi }{2}\\ \frac{1}{2}\sin\left(\vartheta \right)\frac{\left(a^{2}+1\right)\;e^{a\left(\pi -\vartheta \right)}}{ae^{a\frac{\pi }{2}}+1}\;if\;\frac{\pi }{2}<\vartheta \leq \pi \end{array}\right.$$

The function is symmetrical with respect to ϑ=π/2. The above statement can be also formulated as follows: if a→-∞ one can verify that $f_{\Theta }\left(\vartheta \right)=\left(1/2\right)\left(\delta \left(\vartheta \right)+\delta \left(\vartheta -\pi \right)\right)$, where *δ* is the Dirac delta function (perfect order); if a=0 we obtain $f_{\Theta }\left(\vartheta \right)=\sin\left(\vartheta \right)/2$ (complete disorder); finally, if a→+∞ it is possible to show that $f_{\Theta }\left(\vartheta \right)=\delta \left(\vartheta -\pi /2\right)$ and all the particles have the principal axis orthogonal to the *z*-axis.

**Figure 3:**
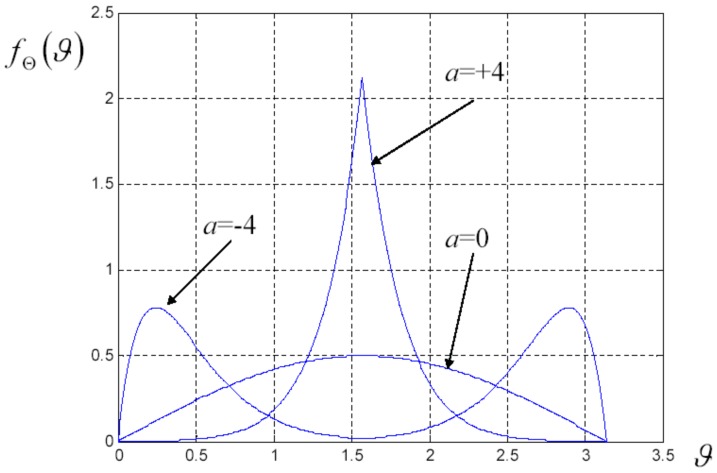
Shape of the probability density defined in Equation (82) in correspondence to three different values of the parameter *a* (*a* = -4, *a* = 0 and *a* = 4).

In Figure 3 one can find the shape of this probability density in correspondence to three different values of the parameter *a* (*a* = -4, *a* = 0 and *a* = 4). One can observe that, for negative values of *a*, we obtain a bimodal density, for *a* = 0 we obtain the sine shaped function $f_{\Theta }\left(\vartheta \right)=\sin\left(\vartheta \right)/2$ and for positive value of *a*, we have a unimodal behavior. This probability density is particularly useful also because it allows computing the order parameters $C_{2}$ and $C_{4}$ in a closed form

(83) $$C_{2}=\int_{0}^{\pi }\cos^{2}\vartheta f_{\Theta }\left(\vartheta \right)d\vartheta =\frac{1}{2}\frac{\left(a^{2}+1\right)\;}{ae^{a\frac{\pi }{2}}+1}\int_{0}^{\pi }\cos^{2}\vartheta \sin\vartheta e^{a\vartheta }d\vartheta =\frac{2ae^{a\frac{\pi }{2}}+a^{2}+3}{\left(a^{2}+9\right)\left(ae^{a\frac{\pi }{2}}+1\right)}$$

(84) $$C_{4}=\int_{0}^{\pi }\cos^{4}\vartheta f_{\Theta }\left(\vartheta \right)d\vartheta =\frac{1}{2}\frac{\left(a^{2}+1\right)\;}{ae^{a\frac{\pi }{2}}+1}\int_{0}^{\pi }\cos^{4}\vartheta \sin\vartheta e^{a\vartheta }d\vartheta =\frac{24ae^{a\frac{\pi }{2}}+a^{4}+22a^{2}+45}{\left(a^{2}+25\right)\left(a^{2}+9\right)\left(ae^{a\frac{\pi }{2}}+1\right)}$$

**Figure 4:**
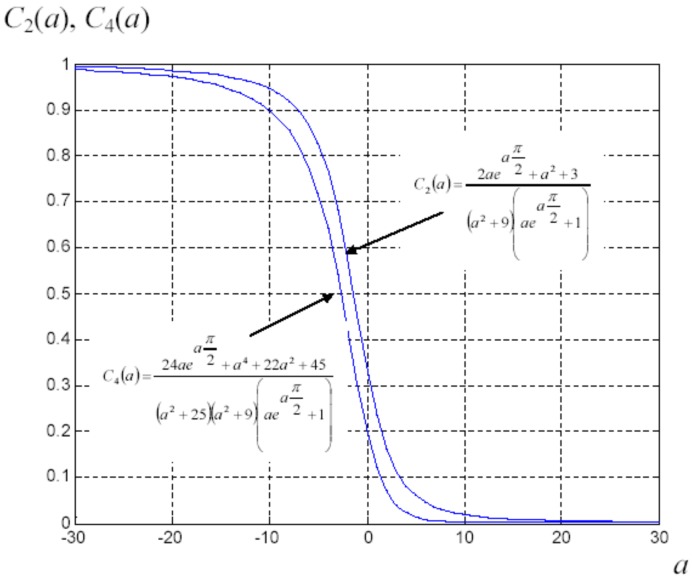
Behavior of the order parameters $C_{2}$ and $C_{4}$ in terms of the coefficient *a* as described by Equations (83) and (84), respectively.

The previous expressions furnish the following special values: if a→-∞ we have $C_{2}=C_{4}=1$, if a=0 we have $C_{2}=1/3$ and $C_{4}=1/5$ and, finally, if a→+∞ we obtain $C_{2}=C_{4}=0$. In Figure 4 one can find the behavior of the coefficients $C_{2}$ and $C_{4}$ versus the parameter *a*.

**Figure 5:**
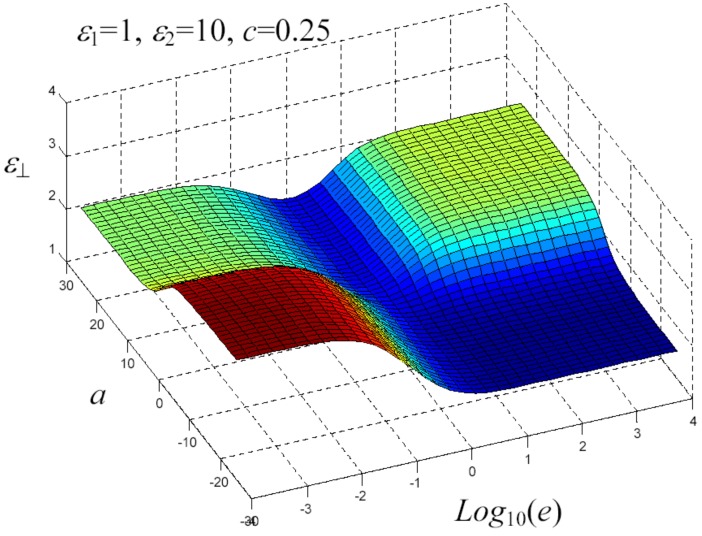
Permittivity $\varepsilon_{⊥}$ versus *a* and *Log*${}_{10}$ (e) for *ε*${}_{1}$ = 1, *ε*${}_{2}$ = 10 and *c* = 0.25.

**Figure 6:**
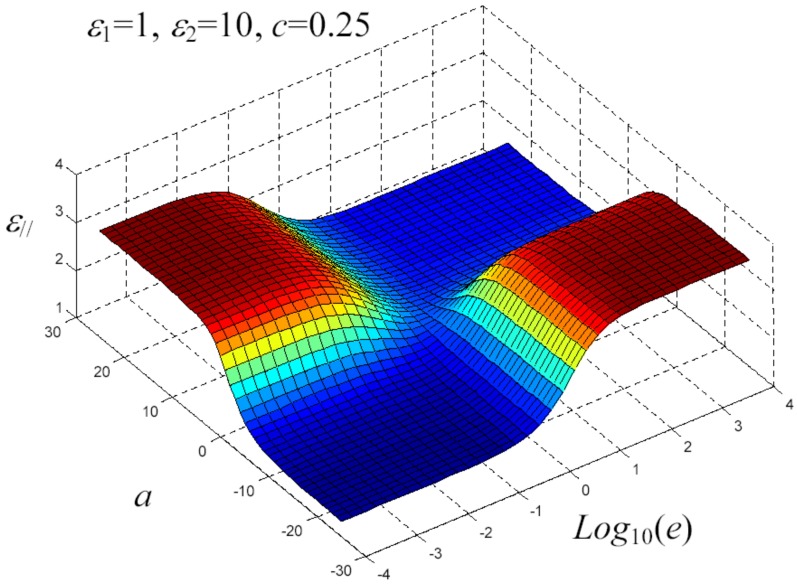
Permittivity $\varepsilon_{//}$ versus *a* and *Log*${}_{10}$ (e) for *ε*${}_{1}$ = 1, *ε*${}_{2}$ = 10 and *c* = 0.25.

**Figure 7:**
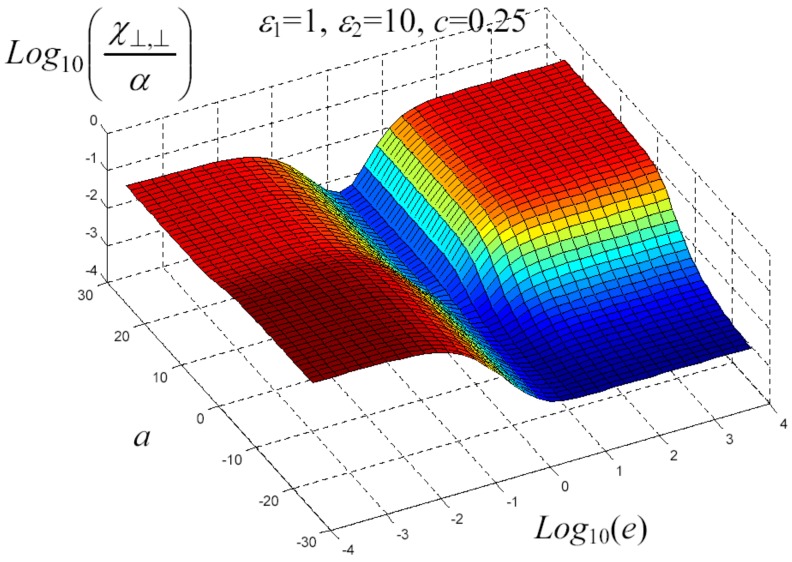
Susceptibility amplification $Log_{10}\left(\chi_{⊥,⊥}/\alpha \right)$ versus *a* and *Log*${}_{10}$ (e) for *ε*${}_{1}$ = 1, *ε*${}_{2}$ = 10 and *c* = 0.25.

**Figure 8:**
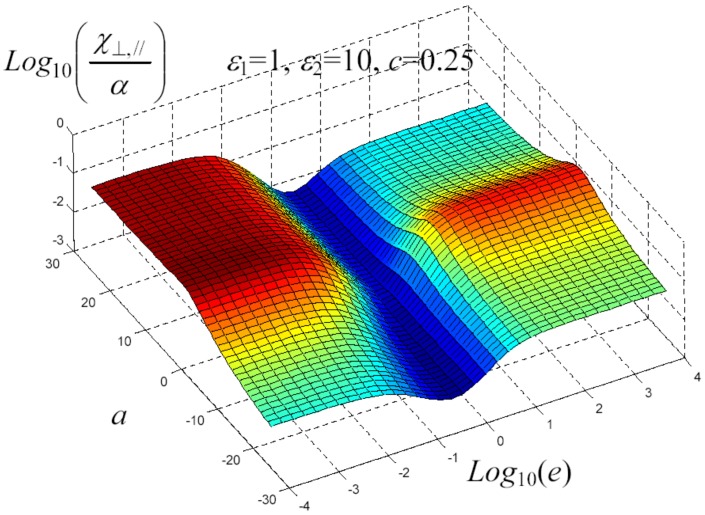
Susceptibility amplification $Log_{10}\left(\chi_{⊥,//}/\alpha \right)$ versus *a* and *Log*${}_{10}$ (e) for *ε*${}_{1}$ = 1, *ε*${}_{2}$ = 10 and *c* = 0.25.

**Figure 9:**
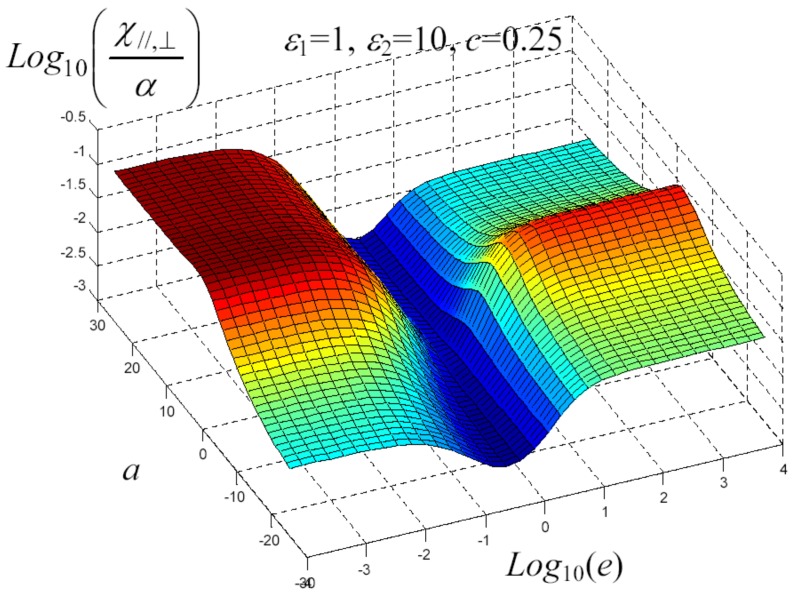
Susceptibility amplification $Log_{10}\left(\chi_{//,⊥}/\alpha \right)$ versus *a* and *Log*${}_{10}$ (e) for *ε*${}_{1}$ = 1, *ε*${}_{2}$ = 10 and *c* = 0.25.

**Figure 10:**
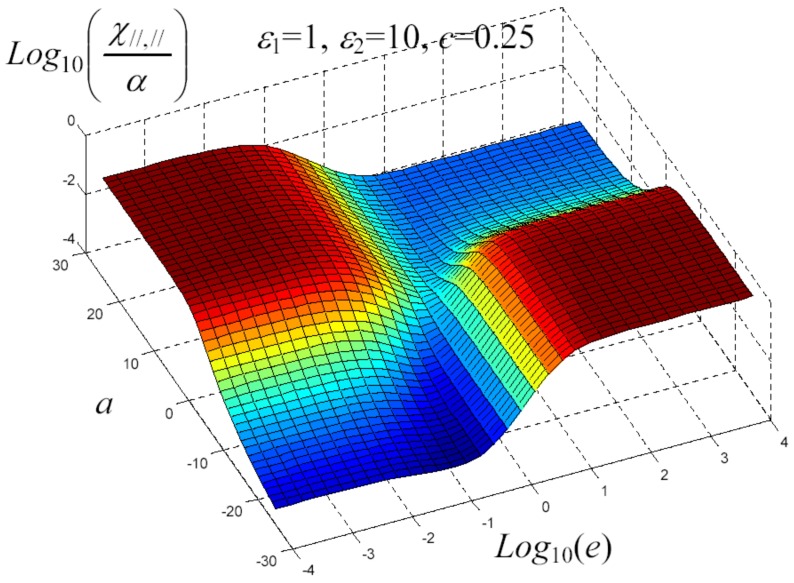
Susceptibility amplification $Log_{10}\left(\chi_{//,//}/\alpha \right)$ versus *a* and *Log*${}_{10}$ (e) for *ε*${}_{1}$ = 1, *ε*${}_{2}$ = 10 and *c* = 0.25.

**Figure 11:**
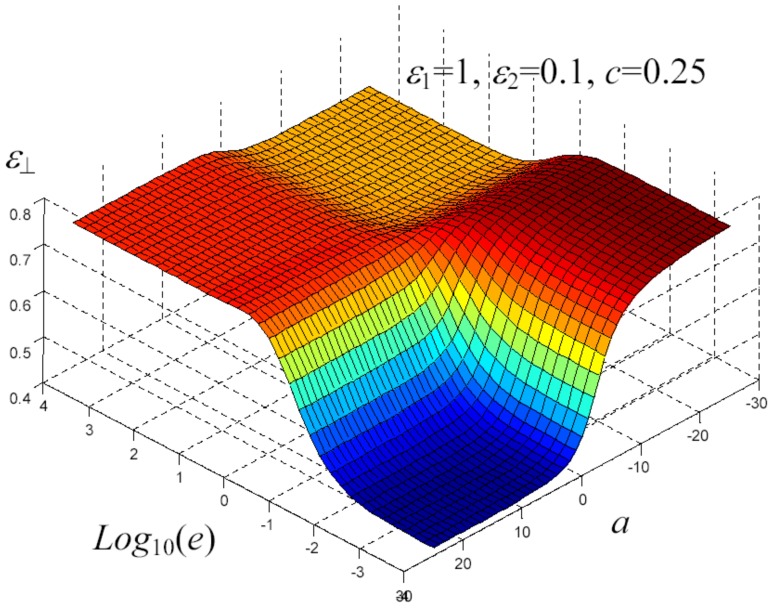
Permittivity $\varepsilon_{⊥}$ versus *a* and *Log*${}_{10}$ (e) for *ε*${}_{1}$ = 1, *ε*${}_{2}$ = 0.1 and *c* = 0.25.

**Figure 12:**
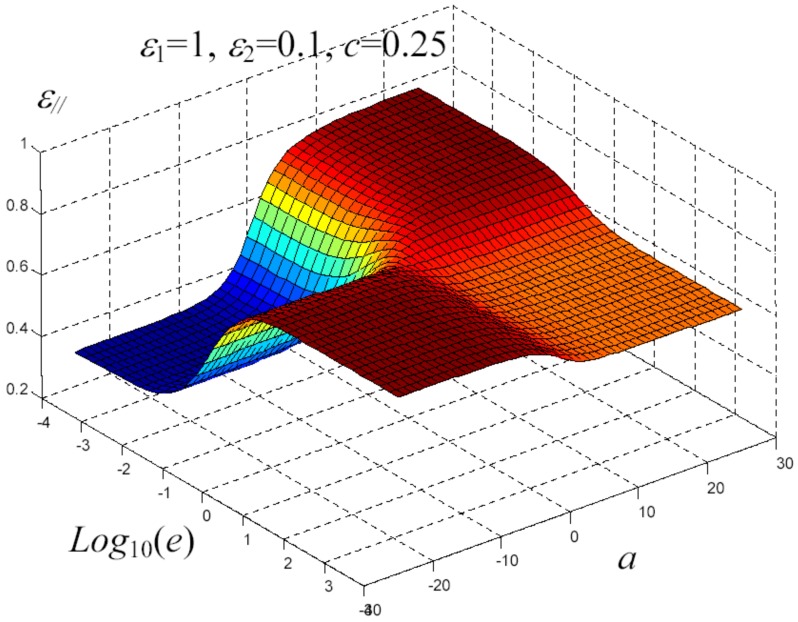
Permittivity $\varepsilon_{//}$ versus *a* and *Log*${}_{10}$ (e) for *ε*${}_{1}$ = 1, *ε*${}_{2}$ = 0.1 and *c* = 0.25.

**Figure 13:**
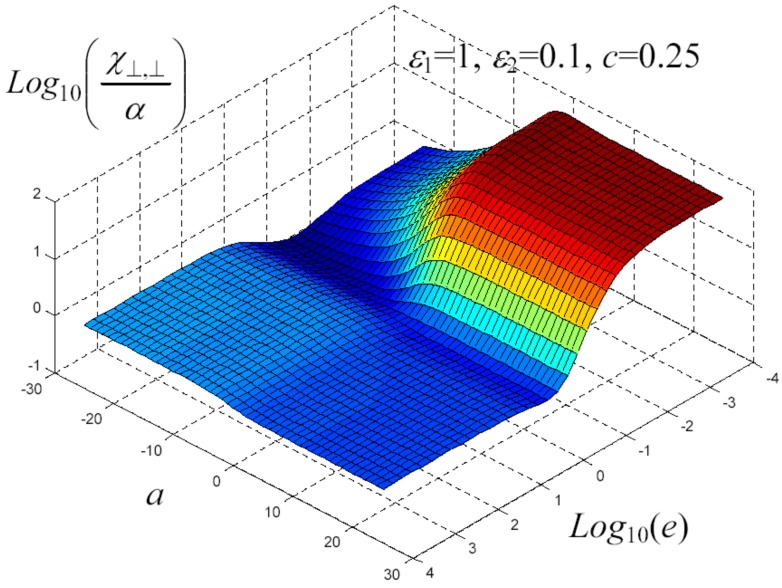
Susceptibility amplification $Log_{10}\left(\chi_{⊥,⊥}/\alpha \right)$ versus *a* and *Log*${}_{10}$ (e) for *ε*${}_{1}$ = 1, *ε*${}_{2}$ = 0.1 and *c* = 0.25.

**Figure 14:**
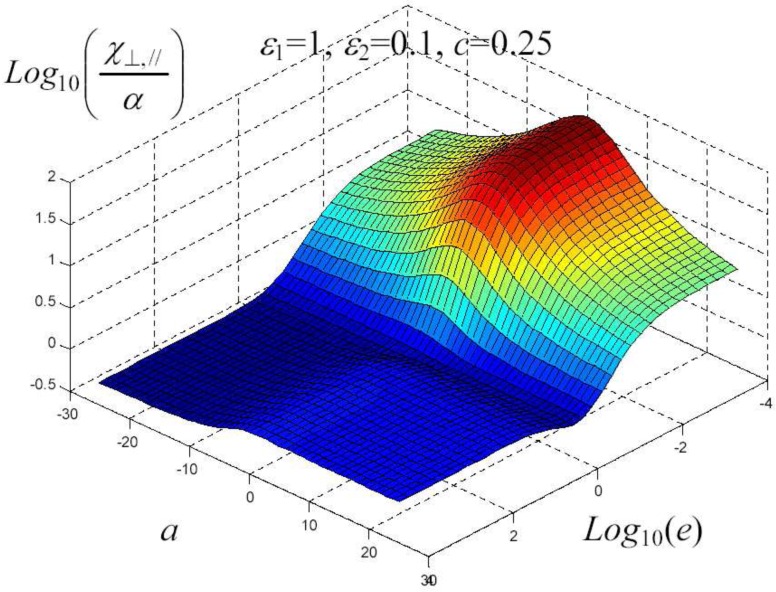
Susceptibility amplification $Log_{10}\left(\chi_{⊥,//}/\alpha \right)$ versus *a* and *Log*${}_{10}$ (e) for *ε*${}_{1}$ = 1, *ε*${}_{2}$ = 0.1 and *c* = 0.25.

**Figure 15:**
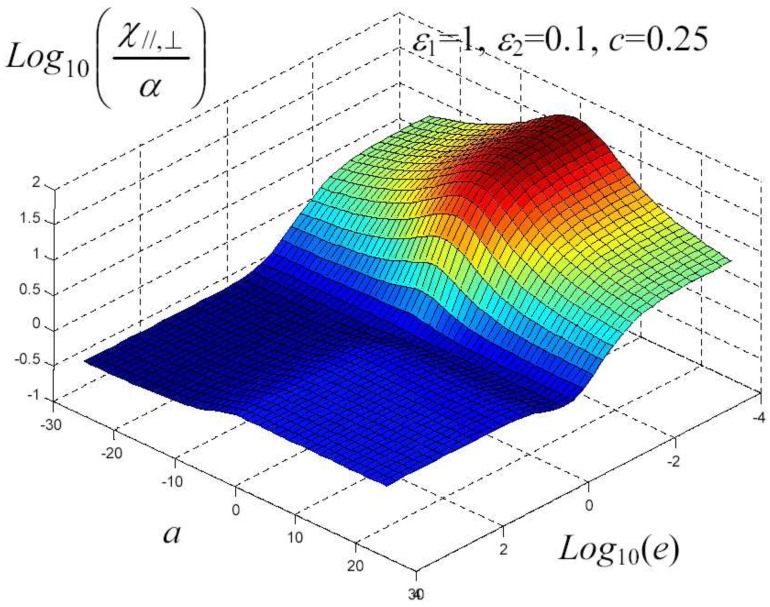
Susceptibility amplification $Log_{10}\left(\chi_{//,⊥}/\alpha \right)$ versus *a* and *Log*${}_{10}$ (e) for *ε*${}_{1}$ = 1, *ε*${}_{2}$ = 0.1 and *c* = 0.25.

We have written a software code that implements the complete procedure summed up in Equation (81), in order to obtain the macroscopic linear and nonlinear features of the composite material in terms of the aspect ratio *e* of the ellipsoids and of the parameter *a* controlling the state of order, as above described. A first series of results concerns the case with $\varepsilon_{1}$ = 1, $\varepsilon_{2}$ = 10 and *c* = 0.25. They are shown in Figure 4, Figure 5, Figure 6, Figure 7, Figure 8 and Figure 9 versus *a* and $Log_{10}\left(e\right)$. More precisely, one can find: in Figure 4 the permittivity $\varepsilon_{⊥}$, in Figure 5 the permittivity $\varepsilon_{//}$, in Figure 6 the susceptibility amplification $Log_{10}\left(\chi_{⊥,⊥}/\alpha \right)$, in Figure 7 the susceptibility amplification $Log_{10}\left(\chi_{⊥,//}/\alpha \right)$, in Figure 8 the susceptibility amplification $Log_{10}\left(\chi_{//,⊥}/\alpha \right)$ and, finally, in Figure 9 the susceptibility amplification $Log_{10}\left(\chi_{//,//}/\alpha \right)$. A second series of results, concerning the case with $\varepsilon_{1}=1$, $\varepsilon_{2}=0.1$ and c=0.25 can be found in Figure 10, Figure 11, Figure 12, Figure 13, Figure 14 and Figure 15. As before, they have been represented in terms of *a* and $Log_{10}\left(e\right)$ and we have adopted the same ordering for the plots. We may draw a comparison among the results obtained when $\varepsilon_{2}/\varepsilon_{1}=10$ and the results obtained when $\varepsilon_{2}/\varepsilon_{1}=1/10$: in both cases, as for the longitudinal and transversal permittivities, we may observe that the effect of the order/disorder has opposite behavior for prolate and oblate particles. Moreover, the complex behavior of the susceptibility amplifications is inverted moving from the case with $\varepsilon_{2}/\varepsilon_{1}=10$ to the case with $\varepsilon_{2}/\varepsilon_{1}=1/10$. The plots exhibit a very complex scenario for the macroscopic properties of the nonlinear material, strongly dependent on the state of order and on the geometric features of the embedded ellipsoids of revolution (prolate or oblate).

We draw some conclusions about the homogenization procedure described. We have analyzed the nonlinear dielectric effects of the orientational order/disorder of non-spherical particles in composite or heterogeneous materials. As result of this analysis we have found the correct definition of two order parameters ($C_{2}$ and $C_{4}$) in such a way to predict the macroscopic electric properties as function of the state of microscopic order. In particular, we have found out explicit relationships that allow us the calculation of the linear permittivity tensor and the nonlinear susceptibility tensor in terms of the shape of the embedded particles and the order parameters. We have outlined and applied a complete procedure which takes into account any given orientational distribution of ellipsoids in the matrix. The theory can find many applications to real physical situations ranging from technological aspects of composite materials to optical characterization of nematic liquid crystals and to tissues modeling in biophysics.

**Figure 16:**
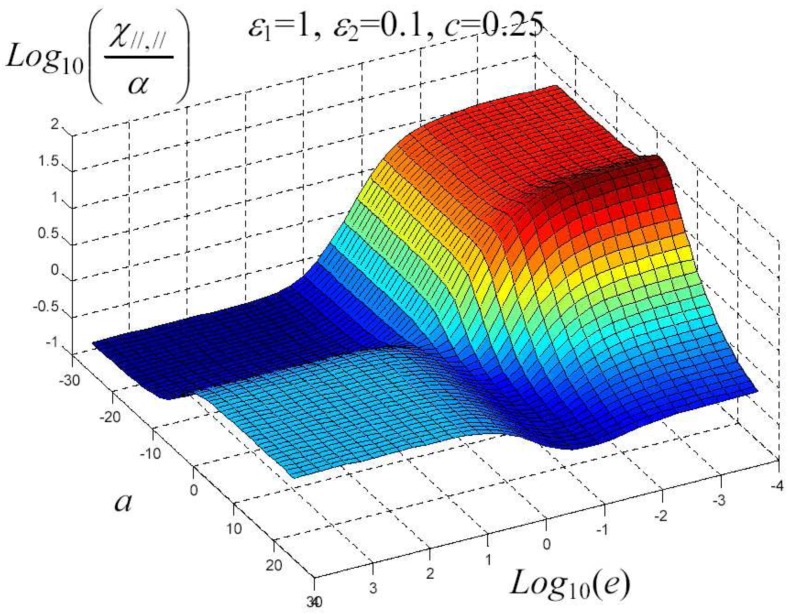
Susceptibility amplification $Log_{10}\left(\chi_{//,//}/\alpha \right)$ versus *a* and *Log*${}_{10}$ (e) for *ε*${}_{1}$ = 1, *ε*${}_{2}$ = 0.1 and *c* = 0.25.

## 8. Nonlinear Elastic Homogenization

The theoretical approaches utilized to analyze nonlinear elastic composite materials are typically based on rigorous variational principles which, in addition to possessing mathematical rigor, have the advantage of leading to bounds and relatively accurate estimates for the mechanical properties. Such variational principles allow one to obtain estimates of the effective energy densities of nonlinear materials in terms of the corresponding information for linear composites with the same microstructure. These methodologies can be found on an excellent review by Ponte Castañeda and Suquet [64]. From the historical point of view, the variational procedure of Hashin and Shtrikman [3,4] is the first important result concerning the linear behavior of electric and elastic heterogeneous materials. This variational procedure provides lower and upper bounds on the elastic moduli and elastic tensors for isotropic composites (reinforced by randomly positioned particles). A generalization of the Hashin-Shtrikman variational principles, suitable for nonlinear materials, was developed by Talbot and Willis [65,66]. This extension can be used to obtain improved bounds (depending on a two-point statistical information) for nonlinear composites. Variational methods for deriving improved bounds and estimates for the effective properties of nonlinear materials, utilizing the effective modulus tensor of suitably selected linear-elastic comparison materials, were introduced by Ponte Castañeda [67,68,69,70] for materials with isotropic phases and by Suquet [71] for composites with power-law phases. Moreover, a hybrid of the Talbot-Willis and Ponte Castañeda procedures, using a linear thermoelastic comparison material, was proposed by Talbot and Willis [72]. An important advantage of the variational procedures that involve linear comparison materials is that, they can not only produce the nonlinear Hashin-Shtrikman-type bounds of the Talbot-Willis procedure directly from the corresponding linear Hashin-Shtrikman bounds, but also yield higher-order nonlinear bounds, such as Beran-type bounds [73], as well as other types of estimates.

The general nonlinear elastic features are relevant in many materials science problems. For example, in biomechanics, transient elastography has shown its efficiency to map the nonlinear properties of soft tissues and it can be used as diagnostic technique [74,75]. In material science the linear theory is incapable of fully capturing all fracture phenomena and hyperelasticity plays a governing role in the dynamics of fracture [76,77]. The quantum dots growth, ordering and orientation (occurring during processing) are largely affected by elastic phenomena, even beyond the linear regime [78,79]. Finally, many problems of fracture mechanics in composite materials do contain nonlinear features like, e.g., the interaction between a crack and a fiber (or, more generally, an inclusion) [80].

The aim of the remaining part of the present work is to review the elastic properties of dispersions of nonlinear elastic inclusions embedded in a linear elastic hosting matrix. Here, we do not apply methodologies based on variational principles since we can obtain, in this particular case, the estimate of the effective elastic behavior by means of the direct calculations of the elastic fields. Therefore, we describe a procedure similar to that utilized, in the previous sections, for the dielectric homogenization. In particular, we will study the elastic fields induced in a single nonlinear particle and then we use such results to homogenize complex dispersions. It is known that the concept of nonlinearity can be introduced in the theory of elasticity in two different ways [81]. A first nonlinearity can be taken into account by means of the exact relation for the strain (not limited for small deformation) and the exact equilibrium equations for a volume element of the body (this first aspect is referred to as geometrical nonlinearity since it is related to the equations not depending on the material under consideration). Secondly, another nonlinear effect can be considered through the arbitrariness of the (generically not Hookean) stress-strain constitutive relation (this aspect is referred to as physical nonlinearity since it is related to properties of the material under consideration). Therefore, by combining the two previous contributions, it follows that there are four different types of problems in the theory of elasticity [82]

- those having both physical and geometrical linearity;
- those which are physically nonlinear but geometrically linear;
- those linear physically but nonlinear geometrically;
- those nonlinear both physically and geometrically.

The problems of the first type are the subject of the (classical) theory of elasticity (small deformation in Hookean materials). In this review, we adopt the second conceptual framework. The angles of rotation can be neglected in determining changes in dimensions in the line elements and in formulating the conditions of equilibrium of a volume elements: therefore, the balance equations are based on the standard small-strain tensor and on the Cauchy stress tensor (typically introduced in the problem of the first type). However, the elongations exceed the Hookean limit of proportionality (between stress and strain) and this requires a nonlinear stress-strain relationship. This conceptual framework is sometimes referred to as *hypoelasticity*: it is intended to model perfectly reversible nonlinear stress-strain behavior but restricted to infinitesimal strains. Such a description has been already adopted in the past in order to model nonlinear cubic polycrystals with perturbative and self-consistent methods [83].

In the following we outline a complete homogenizing procedure for two nonlinear composite materials that paradigmatically represent most features of the above described examples. Firstly, we consider a dispersion of nonlinear (but isotropic, i.e., amorphous or polycrystalline) spheres embedded in a linear homogeneous and isotropic matrix. The nonlinearity of the spheres can be described, at most, by four parameters (the so-called Landau coefficients) measuring the deviation from the linearity. Since the overall behavior of the heterogeneous structure will be elastically nonlinear, the key point is the evaluation of the effective nonlinear properties of the composite material. Secondly, a similar procedure has been developed for a distribution of parallel (nonlinear) cylinders embedded in a (linear) matrix. In both geometries, the most important methodological aspect is given by a useful generalization of the Eshelby theory [84] to nonlinear inhomogeneities.

## 9. Nonlinear Elastic Constitutive Equations

In geometrically linear elasticity, the balance of linear and angular momentum hold for all materials, regardless of their constitution.

The balance of the linear momentum leads to the equation of motion in the form $\frac{\partial T_{ij}}{\partial x_{j}}+b_{j}=\rho \frac{\partial^{2}u_{i}}{\partial t^{2}}$ where the $T_{ij}$ are the components of the Cauchy stress tensor $\hat{T}$, $b_{j}$ are the components of the externally applied body force $\vec{b}$, *ρ* is the mass density and $u_{i}$ are the components of the displacement $\vec{u}$ [81]. The balance of the angular momentum leads to the symmetry of the stress tensor ($T_{ij}=T_{ji}$). However, these relations are generally insufficient to determine the elastic fields produced by given boundary conditions and body forces. They need to be supplemented by a further set of relations, referred to as constitutive equations, which characterize the constitution of the body. The convenient starting point is a set of relations in which the stress components are regarded as single-valued functions of the strain components

(85) $$T_{ij}=f_{ij}\left(\hat{\epsilon }\right)\;\;\;\mathrm{or}\;\;\;\hat{T}=f\left(\hat{\epsilon }\right)$$

where the functions $f_{ij}$ are chosen so that $f_{ij}=f_{ji}$ in order to satisfy the stress symmetry and $\hat{\epsilon }$ is the small-strain tensor with components $\epsilon_{ij}=\frac{1}{2}\left(\frac{\partial u_{i}}{\partial x_{j}}+\frac{\partial u_{j}}{\partial x_{i}}\right)$ [81].

For example, in physically linear elasticity the tensor relationship

(86) $$T_{ij}=C_{ijkh}\epsilon_{kh}$$

is taken into consideration in order to describe any kind of anisotropy. The stiffness tensor $C_{ijkh}$ must fulfill the following constraints:

- $C_{ijkh}=C_{jikh}$ and $C_{ijkh}=C_{ijhk}$, in order to preserve the symmetry of the stress tensor and of the strain tensor;
- $C_{ijkh}=C_{khij}$, derived by the existence of an elastic energy density (Green hypothesis).

For isotropic materials the previous considerations lead to the notable stress-strain relation $\hat{T}=2\mu \hat{\epsilon }+\lambda \mathrm{Tr}\left(\hat{\epsilon }\right)\hat{I}$, which is based on the two independent Lamè constants *λ* and *μ* [85,86].

When considering nonlinear elastic material, Equation (86) can be generalized by means of higher order elastic moduli taking into account the deviation from the stress-strain proportionality [87,88]

(87) $$\begin{array}{c} T_{ij}=C_{ijkh}\epsilon_{kh}+\frac{1}{2}L_{ijkhnm}\epsilon_{kh}\epsilon_{nm}+...=\left[C_{ijkh}+\frac{1}{2}L_{ijkhnm}\epsilon_{nm}+...\right]\epsilon_{kh}=C_{ijkh}^{NL}\left(\hat{\epsilon }\right)\epsilon_{kh} \end{array}$$

where ${\hat{C}}^{NL}\left(\hat{\epsilon }\right)$ is the nonlinear (strain dependent) stiffness tensor. It can be noticed that the tensor $\hat{C}$ has 21 independent entries, while the second order tensor $\hat{L}$ has 56 independent components. Tables for the values of $C_{ijkh}$ and $L_{ijkhnm}$ can be found in literature [83]. These values can be obtained by experimental procedure [89,90] and by computational techniques (e.g., molecular dynamics [91] or first-principles calculations [92]).

For the following purposes we are interested in the isotropic nonlinear constitutive equations expanded up to the second order in the strain components. In order to introduce these forms of physical nonlinearities we can take into account two different approaches, as described below.

### 9.1. Cauchy elasticity

The Cauchy approach to the constitutive equations is the less restrictive starting point for the elasticity theory since it does not consider the strain energy function. It is simply based on the Equation (85). To develop this approach in an isotropic context an assumption must be made concerning the behavior of Equation (85) under rigid-body rotations. The function $f\left(\hat{\epsilon }\right)$ must satisfy the identity [81]

(88) $${\hat{R}}^{T}f\left(\hat{\epsilon }\right)\hat{R}=f\left({\hat{R}}^{T}\hat{\epsilon }\hat{R}\right)$$

for all proper orthogonal tensor $\hat{R}$ representing the rotation. A function satisfying the previous identity is known as an isotropic tensor function, and it can be represented in the form [81]

(89) $$\hat{T}=f\left(\hat{\epsilon }\right)=q_{1}\hat{I}+q_{2}\hat{\epsilon }+q_{3}{\hat{\epsilon }}^{2}$$

where $\hat{I}$ is the identity operator and $q_{1}$, $q_{2}$ and $q_{3}$ are scalar functions of the invariants $\mathrm{Tr}(\hat{\epsilon })$, $\mathrm{Tr}({\hat{\epsilon }}^{2})$ e $\mathrm{Tr}({\hat{\epsilon }}^{3})$ of the strain tensor $\hat{\epsilon }$

(90) $$q_{\alpha }=q_{\alpha }\left(\mathrm{Tr}\left(\hat{\epsilon }\right),\mathrm{Tr}\left({\hat{\epsilon }}^{2}\right),\mathrm{Tr}\left({\hat{\epsilon }}^{3}\right)\right)$$

The development of Equation (89), up to the second order in the powers of $\hat{\epsilon }$, provides the following constitutive equation

(91) $$\begin{array}{ccc} \hat{T} & = & 2\mu \hat{\epsilon }+\lambda \mathrm{Tr}\left(\hat{\epsilon }\right)\hat{I}+A{\hat{\epsilon }}^{2}+B\mathrm{Tr}\left({\hat{\epsilon }}^{2}\right)\hat{I}+C{\left[\mathrm{Tr}\left(\hat{\epsilon }\right)\right]}^{2}\hat{I}+D\hat{\epsilon }\mathrm{Tr}\left(\hat{\epsilon }\right) \end{array}$$

where *μ* and *λ* are the standard Lamè moduli concerning the linear contribution and A,B,C and *D* are the coefficients describing the nonlinear behavior of the material.

### 9.2. Green elasticity

The Green elasticity is based on Equation (85) with an additional hypothesis: we suppose that the stress power, in a given deformation, is absorbed into a strain energy function $U(\hat{\epsilon })$, representing the density of elastic potential energy. The existence of such a function and the consideration of energy balance in the continuum, lead to the evolution equation [85]

(92) $$\frac{dU(\hat{\epsilon })}{dt}=T_{ij}\left(\hat{\epsilon }\right)\frac{d\epsilon_{ij}}{dt}$$

affirming that the function $U(\hat{\epsilon })$ is an exact differential form such that

(93) $$T_{ij}\left(\hat{\epsilon }\right)=\frac{\partial U(\hat{\epsilon })}{\partial \epsilon_{ij}}$$

So, if a function $U(\hat{\epsilon })$ exists, the (arbitrarily nonlinear) constitutive equation for a given material can be determined by Equation (93) [85,86]. From the thermodynamics point of view, the strain energy function can be identified with the internal energy per unit volume in an isentropic process, or with the Helmholtz free energy per unit volume in an isothermal process. Such an approach can be further developed for isotropic media: in this case, the function $U(\hat{\epsilon })$ must satisfy the relation [81]

(94) $$U\left(\hat{\epsilon }\right)=U\left({\hat{R}}^{T}\hat{\epsilon }\hat{R}\right)$$

for any rotation tensor $\hat{R}$. Equation (94) represents the scalar counterpart of the tensor relation Equation (88). If Equation (94) is true then it follows that the function $U(\hat{\epsilon })$ can depend only on the principal invariants of the strain tensor

(95) $$U=U\left(\mathrm{Tr}\left(\hat{\epsilon }\right),\mathrm{Tr}\left({\hat{\epsilon }}^{2}\right),\mathrm{Tr}\left({\hat{\epsilon }}^{3}\right)\right)$$

We may expand Equation (95) up to the third order in the strain components, obtaining [86]

(96) $$\begin{array}{ccc} U(\hat{\epsilon }) & = & \mu \mathrm{Tr}\left({\hat{\epsilon }}^{2}\right)+\frac{\lambda }{2}{\left[\mathrm{Tr}\left(\hat{\epsilon }\right)\right]}^{2}+\frac{A}{3}\mathrm{Tr}\left({\hat{\epsilon }}^{3}\right)+B\mathrm{Tr}\left(\hat{\epsilon }\right)\mathrm{Tr}\left({\hat{\epsilon }}^{2}\right)+\frac{C}{3}{\left[\mathrm{Tr}\left(\hat{\epsilon }\right)\right]}^{3} \end{array}$$

Finally, performing the derivatives indicated in Equation (93), we obtain the nonlinear isotropic constitutive equation (within the Green approach) expanded up to the second order in the strain tensor

(97) $$\begin{array}{ccc} \hat{T} & = & 2\mu \hat{\epsilon }+\lambda \mathrm{Tr}\left(\hat{\epsilon }\right)\hat{I}+A{\hat{\epsilon }}^{2}+B\left\{\mathrm{Tr}\left({\hat{\epsilon }}^{2}\right)\hat{I}+2\hat{\epsilon }\mathrm{Tr}\left(\hat{\epsilon }\right)\right\}+C{\left[\mathrm{Tr}\left(\hat{\epsilon }\right)\right]}^{2}\hat{I} \end{array}$$

It is evident by comparison of Equation (91) and Equation (97) that the Green elasticity is more restrictive than the Cauchy elasticity: we obtain the Green formulation from the Cauchy formulation by imposing D=2B. We use four independent parameters (A,B,C and *D*) in the Cauchy elasticity and three independent parameters (A,B and *C*) in the Green elasticity. These parameters are called Landau coefficients [86].

## 10. Eshelby Theory for Nonlinear Inhomogeneities

**Figure 17:**
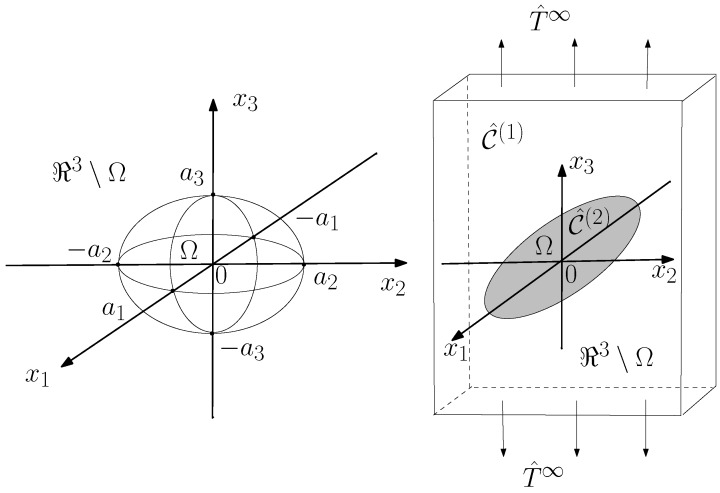
Scheme of an ellipsoidal inhomogeneity.

A nonlinear isotropic and homogenous ellipsoid can be generically described by the relation $\hat{T}={\hat{C}}^{\left(2\right)}\left(\hat{\epsilon }\right)\hat{\epsilon }$ (see Equation (87)). Let us now place this inhomogeneity in a linear matrix characterized by a stiffness tensor ${\hat{C}}^{\left(1\right)}$ (see Figure 17) and let us calculate the strain field inside the particle when a uniform field ${\hat{T}}^{\infty }={\hat{C}}^{\left(1\right)}{\hat{\epsilon }}^{\infty }$ is remotely applied to the system.

If the particle were linear, with ${\hat{C}}^{\left(2\right)}$ independent from the strain, we would have, inside the ellipsoid, a uniform strain field ${\hat{\epsilon }}^{s}$ given by the Eshelby theory [93,94]

(98) $${\hat{\epsilon }}^{s}={\left[\hat{I}-\hat{S}\left(\hat{I}-{\left({\hat{C}}^{\left(1\right)}\right)}^{-1}{\hat{C}}^{\left(2\right)}\right)\right]}^{-1}{\hat{\epsilon }}^{\infty }$$

where, we have introduced the Eshelby tensor $\hat{S}$, which depends only on geometrical factors of the ellipsoid (the semi-axes $a_{1}$, $a_{2}$ and $a_{3}$) and on the Poisson ratio of the host matrix [84]. Conversely, if the ellipsoid were nonlinear, it is easy to prove that the internal uniform field must satisfy the equation

(99) $${\hat{\epsilon }}^{s}={\left[\hat{I}-\hat{S}\left(\hat{I}-{\left({\hat{C}}^{\left(1\right)}\right)}^{-1}{\hat{C}}^{\left(2\right)}\left({\hat{\epsilon }}^{s}\right)\right)\right]}^{-1}{\hat{\epsilon }}^{\infty }$$

obtained form Equation (98) through the substitution ${\hat{C}}^{\left(2\right)}\to {\hat{C}}^{\left(2\right)}\left({\hat{\epsilon }}^{s}\right)$. If a solution ${\hat{\epsilon }}^{s*}$ exists for a given ${\hat{\epsilon }}^{\infty }$, it means that the nonlinear inhomogeneity could be replaced by a linear one with constant stiffness ${\hat{C}}^{\left(2\right)}={\hat{C}}^{\left(2\right)}\left({\hat{\epsilon }}^{s*}\right)$, without modifications of the elastic fields at any point. Therefore, if a solution exists, then Equation (99) exactly describes, through self-consistency, the elastic behavior of the nonlinear anisotropic inclusion. This is not a trivial result: for instance, such a generalization of Equation (98) is not valid if a nonlinear behavior is assumed for material 1 (matrix). The calculation of the internal strain field from Equation (99) is very complicated and it strongly depends on the kind of nonlinearity $\hat{T}={\hat{C}}^{\left(2\right)}\left(\hat{\epsilon }\right)\hat{\epsilon }$. This task will be accomplished in the following, dealing with a sphere or a cylinder described by physical nonlinearities as those in Equation (91) (Cauchy) or Equation (97) (Green).

To conclude, we have proved the following general statement: if the linear elastic space with a single inhomogeneity of ellipsoidal shape is subjected to remote uniform loading, the stress field inside the inhomogeneity will be uniform independent of the constitutive law for the inhomogeneity, provided that both the matrix and the particle are homogeneous bodies. Some similar properties can be found in earlier literature [95,96,97].

When the Green approach is considered it is also possible to verify the existence and the uniqueness for the solution of Equation (99) [98,99]. The proof follows.

### 10.1. Nonlinear Eshelby theory within green elasticity

We adopt here, from the energetic point of view, the Green formulation of the elasticity theory. A strain energy function $U(\hat{\epsilon })$ defines the constitutive equation $\hat{T}\left(\hat{\epsilon }\right)=\frac{\partial U(\hat{\epsilon })}{\partial \hat{\epsilon }}$ of the inhomogeneity, which is equivalent to $\hat{T}\left(\hat{\epsilon }\right)={\hat{C}}^{\left(2\right)}\left(\hat{\epsilon }\right)\hat{\epsilon }$. In these conditions, the existence and uniqueness of a solution for Equation (99) can be exactly proved under the sole hypothesis of convexity for the strain energy function $U(\hat{\epsilon })$ [98,99]. To prove this statement, we rearrange Equation (99) as follows

(100) $$\begin{array}{ccc} \left\{\hat{I}-\hat{S}\left[\hat{I}-{\left({\hat{C}}^{\left(1\right)}\right)}^{-1}{\hat{C}}^{\left(2\right)}\left({\hat{\epsilon }}^{s}\right)\right]\right\}{\hat{\epsilon }}^{s} & = & {\hat{\epsilon }}^{\infty }\\ {\hat{\epsilon }}^{s}-\hat{S}\left[\hat{I}-{\left({\hat{C}}^{\left(1\right)}\right)}^{-1}{\hat{C}}^{\left(2\right)}\left({\hat{\epsilon }}^{s}\right)\right]{\hat{\epsilon }}^{s} & = & {\hat{\epsilon }}^{\infty }\\ {\hat{\epsilon }}^{s}-\hat{S}{\hat{\epsilon }}^{s}+\hat{S}{\left({\hat{C}}^{\left(1\right)}\right)}^{-1}{\hat{C}}^{\left(2\right)}\left({\hat{\epsilon }}^{s}\right){\hat{\epsilon }}^{s} & = & {\hat{\epsilon }}^{\infty }\\ \left[\hat{I}-\hat{S}\right]{\hat{\epsilon }}^{s}+\hat{S}{\left({\hat{C}}^{\left(1\right)}\right)}^{-1}\frac{\partial U({\hat{\epsilon }}^{s})}{\partial {\hat{\epsilon }}^{s}} & = & {\hat{\epsilon }}^{\infty }\\ {\hat{C}}^{\left(1\right)}\left[{\hat{S}}^{-1}-\hat{I}\right]{\hat{\epsilon }}^{s}-{\hat{C}}^{\left(1\right)}{\hat{S}}^{-1}{\hat{\epsilon }}^{\infty }+\frac{\partial U({\hat{\epsilon }}^{s})}{\partial {\hat{\epsilon }}^{s}} & = & 0 \end{array}$$

Now, the first linear term can be converted to the gradient of a quadratic form and the second constant term can be converted to the gradient of a linear form. At the end we observe that the internal strain field must satisfy the following relation [98,99]

(101) $$\frac{\partial }{\partial \hat{\epsilon }}\left\{\frac{1}{2}\hat{\epsilon }{\hat{C}}^{\left(1\right)}\left[{\hat{S}}^{-1}-\hat{I}\right]\hat{\epsilon }-\hat{\epsilon }{\hat{C}}^{\left(1\right)}{\hat{S}}^{-1}{\hat{\epsilon }}^{\infty }+U\left(\hat{\epsilon }\right)\right\}=0$$

which is exactly equivalent to Equation (99). The first term represents a symmetric and positive definite quadratic form in $\hat{\epsilon }$ (see Appendix A) while the second term is a linear function of $\hat{\epsilon }$. Therefore, the sum of these two terms is a convex functional with relative minimum at $\left[\hat{I}-\hat{S}\right]{\hat{\epsilon }}^{\infty }$. This value represents the strain field in a void (${\hat{C}}^{\left(2\right)}\left(\hat{\epsilon }\right)=0$ in Equation (99) or $U(\hat{\epsilon })=0$ in Equation (101)) embedded in the matrix with stiffness ${\hat{C}}^{\left(1\right)}$. If $U(\hat{\epsilon })$ is a convex functional (with U(0)=0) the brackets in Equation (101) contain the sum of two convex terms: they result in an overall convex functional with a unique minimal extremum at ${\hat{\epsilon }}^{s}$.

## 11. Elastic Dispersion of Nonlinear Spherical Inhomogeneities

**Figure 18:**
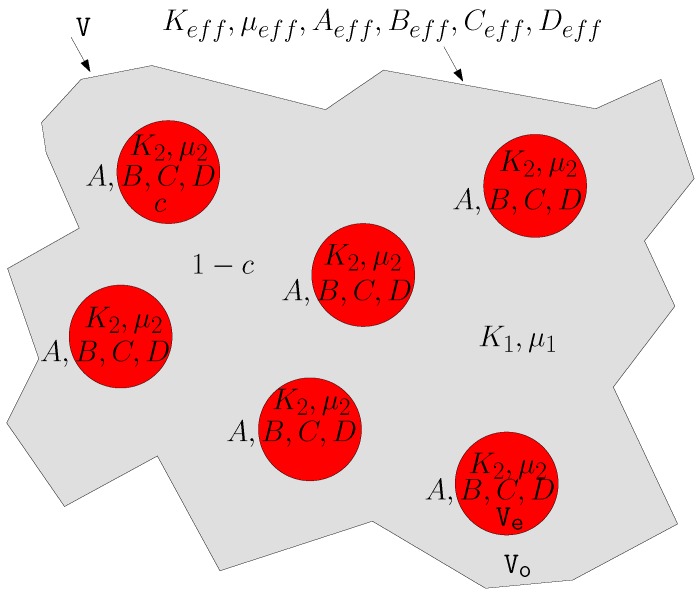
Scheme of a dispersion of nonlinear spheres embedded in a linear matrix.

We consider an assembly of spherical inhomogeneities (see Figure 18) described by a Cauchy constitutive relation

(102) $$\begin{array}{ccc} {\hat{T}}^{s} & = & 2\mu_{2}{\hat{\epsilon }}^{s}+\lambda_{2}\mathrm{Tr}\left({\hat{\epsilon }}^{s}\right)\hat{I}+A{\left({\hat{\epsilon }}^{s}\right)}^{2}+B\mathrm{Tr}\left[{\left({\hat{\epsilon }}^{s}\right)}^{2}\right]\hat{I}+C{\left[\mathrm{Tr}\left({\hat{\epsilon }}^{s}\right)\right]}^{2}\hat{I}+D{\hat{\epsilon }}^{s}\mathrm{Tr}\left({\hat{\epsilon }}^{s}\right) \end{array}$$

randomly embedded in a linear matrix with stiffness tensor ${\hat{C}}^{\left(1\right)}$ (moduli $\lambda_{1}$ and $\mu_{1}$). We also introduce the bulk moduli $K_{1}=\lambda_{1}+\frac{2}{3}\mu_{1}$ and $K_{2}=\lambda_{2}+\frac{2}{3}\mu_{2}$. If needed, we can easily move to the Green elasticity by assuming D=2B. We suppose that the volume fraction *c* of the embedded phase is small (dilute dispersion). Since the elastic interactions can be neglected, each sphere behaves as an isolated one under the effect of a remote load ${\hat{T}}^{\infty }={\hat{C}}^{\left(1\right)}{\hat{\epsilon }}^{\infty }$. The starting point for the evaluation of the induced internal strain ${\hat{\epsilon }}^{s}$ is Equation (99), which can be usefully rearranged as follows

(103) $${\hat{\epsilon }}^{s}-\hat{S}{\hat{\epsilon }}^{s}+\hat{S}{\left({\hat{C}}^{\left(1\right)}\right)}^{-1}{\hat{T}}^{s}={\hat{\epsilon }}^{\infty }$$

Here, we have introduced the internal stress given by the relation ${\hat{T}}^{s}={\hat{C}}^{\left(2\right)}\left({\hat{\epsilon }}^{s}\right){\hat{\epsilon }}^{s}$. The result of the application of ${\left({\hat{C}}^{\left(1\right)}\right)}^{-1}$ over the stress tensor ${\hat{T}}^{s}$ can be easily written in explicit form

(104) $${\left({\hat{C}}^{\left(1\right)}\right)}^{-1}{\hat{T}}^{s}=\frac{1}{2\mu_{1}}{\hat{T}}^{s}-\frac{\lambda_{1}}{2\mu_{1}\left(2\mu_{1}+3\lambda_{1}\right)}\mathrm{Tr}\left({\hat{T}}^{s}\right)\hat{I}$$

Moreover, the explicit expression of the Eshelby tensor for a sphere is reported in literature [28,84]

(105) $$\begin{array}{ccc} S_{ijkh} & = & \frac{1}{15(1-\nu_{1})}\left[\left(\delta_{ik}\delta_{jh}+\delta_{ih}\delta_{jk}\right)\left(4-5\nu_{1}\right)+\delta_{kh}\delta_{ij}\left(5\nu_{1}-1\right)\right] \end{array}$$

We can evaluate the effect of $S_{ijkh}$ over an arbitrary strain $\epsilon_{kh}^{s}$, getting

(106) $$\begin{array}{c} S_{ijkh}\epsilon_{kh}^{s}=\frac{2(4-5\nu_{1})}{15(1-\nu_{1})}\epsilon_{ij}^{s}+\frac{5\nu_{1}-1}{15(1-\nu_{1})}\epsilon_{kk}^{s}\delta_{ij} \end{array}$$

Now, the Poisson ratio $\nu_{1}$ of the matrix can be written in terms of the bulk modulus $K_{1}$ and the shear modulus $\mu_{1}$ through the standard relation $\nu_{1}=\frac{3K_{1}-2\mu_{1}}{2(3K_{1}+\mu_{1})}$, obtaining

(107) $$\begin{array}{c} \hat{S}{\hat{\epsilon }}^{s}=\frac{6}{5}\frac{K_{1}+2\mu_{1}}{3K_{1}+4\mu_{1}}{\hat{\epsilon }}^{s}+\frac{1}{5}\frac{3K_{1}-4\mu_{1}}{3K_{1}+4\mu_{1}}\mathrm{Tr}\left({\hat{\epsilon }}^{s}\right)\hat{I} \end{array}$$

In order to find a single equation for the internal strain ${\hat{\epsilon }}^{s}$, we can substitute Equations (102), (104) and (107) in Equation (103). A long algebraic calculation leads to the important equation [98,99].

(108) $$\begin{array}{ccc} L{\hat{\epsilon }}^{s} & + & M\mathrm{Tr}\left({\hat{\epsilon }}^{s}\right)\hat{I}+N{\left({\hat{\epsilon }}^{s}\right)}^{2}+O{\hat{\epsilon }}^{s}\mathrm{Tr}\left({\hat{\epsilon }}^{s}\right)+P\mathrm{Tr}\left[{\left({\hat{\epsilon }}^{s}\right)}^{2}\right]\hat{I}+Q{\left[\mathrm{Tr}\left({\hat{\epsilon }}^{s}\right)\right]}^{2}\hat{I}={\hat{\epsilon }}^{\infty } \end{array}$$

which completely defines the internal strain induced in a nonlinear sphere by the uniform remote deformation ${\hat{\epsilon }}^{\infty }$. The parameters L,M,N,O,P and *Q* have been written in terms of the shear moduli, bulk moduli and nonlinear coefficients as follows

(109) $$\begin{array}{ccc} L & = & 1+\frac{6}{5}\frac{K_{1}+2\mu_{1}}{3K_{1}+4\mu_{1}}\left(\frac{\mu_{2}}{\mu_{1}}-1\right) \end{array}$$

(110) $$\begin{array}{ccc} M & = & \frac{1}{5\left(3K_{1}+4\mu_{1}\right)}\left[5K_{2}-K_{1}\left(3+2\frac{\mu_{2}}{\mu_{1}}\right)-4\left(\mu_{2}-\mu_{1}\right)\right] \end{array}$$

(111) $$\begin{array}{ccc} N & = & \frac{3}{5}\frac{A}{\mu_{1}}\frac{K_{1}+2\mu_{1}}{3K_{1}+4\mu_{1}} \end{array}$$

(112) $$\begin{array}{ccc} O & = & \frac{3}{5}\frac{D}{\mu_{1}}\frac{K_{1}+2\mu_{1}}{3K_{1}+4\mu_{1}} \end{array}$$

(113) $$\begin{array}{ccc} P & = & \frac{1}{15\left(3K_{1}+4\mu_{1}\right)}\left[15B-A\left(1+3\frac{K_{1}}{\mu_{1}}\right)\right] \end{array}$$

(114) $$\begin{array}{ccc} Q & = & \frac{1}{15\left(3K_{1}+4\mu_{1}\right)}\left[15C-D\left(1+3\frac{K_{1}}{\mu_{1}}\right)\right] \end{array}$$

At this point we take into consideration the actual dispersion of spheres. We define V as the total volume of the composite material, V${}_{e}$ as the volume corresponding to the spheres and V${}_{o}$ as the volume of the matrix ($V=V_{o}\cup V_{e}$, see Figure 18). Since we are working under the hypothesis of small volume fraction *c*, we can consider the average value of the strain in the matrix equal to the externally applied strain ${\hat{\epsilon }}^{\infty }$. Therefore, the average value of the strain in the overall system is given by

(115) $$\begin{array}{c} \langle \hat{\epsilon }\rangle =c{\hat{\epsilon }}^{s}+\left(1-c\right){\hat{\epsilon }}^{\infty } \end{array}$$

On the other hand, the average value of the stress over the entire structure can be calculated as follows

(116) $$\begin{array}{ccc} \langle \hat{T}\rangle & = & \frac{1}{V}\int_{V}\hat{T}dv=\frac{1}{V}{\hat{C}}^{\left(1\right)}\int_{V_{o}}\hat{\epsilon }dv+\frac{1}{V}\int_{V_{e}}\hat{T}dv\\ & = & \frac{1}{V}{\hat{C}}^{\left(1\right)}\int_{V_{o}}\hat{\epsilon }dv+\frac{1}{V}\int_{V_{e}}\hat{T}dv+\frac{1}{V}{\hat{C}}^{\left(1\right)}\int_{V_{e}}\hat{\epsilon }dv-\frac{1}{V}{\hat{C}}^{\left(1\right)}\int_{V_{e}}\hat{\epsilon }dv\\ & = & \frac{1}{V}{\hat{C}}^{\left(1\right)}\int_{V}\hat{\epsilon }dv+\frac{1}{V}\left[\int_{V_{e}}\hat{T}dv+{\hat{C}}^{\left(1\right)}\int_{V_{e}}\hat{\epsilon }dv\right]\\ & = & {\hat{C}}^{\left(1\right)}\langle \hat{\epsilon }\rangle +c\left[{\hat{T}}^{s}-{\hat{C}}^{\left(1\right)}{\hat{\epsilon }}^{s}\right] \end{array}$$

In order to obtain the macroscopic characterization of the material, we search for the relationship between $\langle \hat{T}\rangle$ and $\langle \hat{\epsilon }\rangle$, given in Equations (115) and (116), respectively.

By substituting Equation (108) in Equation (115), we obtain the average strain $\langle \hat{\epsilon }\rangle$ in terms of the internal strain ${\hat{\epsilon }}^{s}$

(117) $$\begin{array}{ccc} \langle \hat{\epsilon }\rangle & = & \left[c+(1-c)L\right]{\hat{\epsilon }}^{s}\\ & + & \left(1-c\right)\left\{M\mathrm{Tr}\left({\hat{\epsilon }}^{s}\right)\hat{I}+N{\left({\hat{\epsilon }}^{s}\right)}^{2}\right.\\ & + & \left.O{\hat{\epsilon }}^{s}\mathrm{Tr}\left({\hat{\epsilon }}^{s}\right)+P\mathrm{Tr}\left[{\left({\hat{\epsilon }}^{s}\right)}^{2}\right]\hat{I}+Q{\left[\mathrm{Tr}\left({\hat{\epsilon }}^{s}\right)\right]}^{2}\hat{I}\right\} \end{array}$$

and by substituting the constitutive relations in Equation (116), we obtain the average stress $\langle \hat{T}\rangle$ in terms of ${\hat{\epsilon }}^{s}$

(118) $$\begin{array}{ccc} \langle \hat{T}\rangle & = & 2\mu_{1}\langle \hat{\epsilon }\rangle +\left(K_{1}-\frac{2}{3}\mu_{1}\right)\mathrm{Tr}\langle \hat{\epsilon }\rangle \hat{I}\\ & + & c\left\{2\left(\mu_{2}-\mu_{1}\right){\hat{\epsilon }}^{s}+\left[K_{2}-K_{1}-\frac{2}{3}\left(\mu_{2}-\mu_{1}\right)\right]\mathrm{Tr}\left({\hat{\epsilon }}^{s}\right)\hat{I}\right.\\ & + & \left.A{\left({\hat{\epsilon }}^{s}\right)}^{2}+B\mathrm{Tr}\left[{\left({\hat{\epsilon }}^{s}\right)}^{2}\right]\hat{I}+C{\left[\mathrm{Tr}\left({\hat{\epsilon }}^{s}\right)\right]}^{2}\hat{I}+D{\hat{\epsilon }}^{s}\mathrm{Tr}\left({\hat{\epsilon }}^{s}\right)\right\} \end{array}$$

The last two expressions, although in implicit form, define the macroscopic constitutive equation relating $\langle \hat{T}\rangle$ and $\langle \hat{\epsilon }\rangle$. In fact, we may obtain ${\hat{\epsilon }}^{s}$ in terms of $\langle \hat{\epsilon }\rangle$ from Equation (117) and this result can be replaced in Equation (118), leading to the final characterization. In order to follow this scheme, we rewrite Equation (117) in a simpler form

(119) $$\begin{array}{ccc} \langle \hat{\epsilon }\rangle & = & L^{\prime }{\hat{\epsilon }}^{s}+M^{\prime }\mathrm{Tr}\left({\hat{\epsilon }}^{s}\right)\hat{I}+N^{\prime }{\left({\hat{\epsilon }}^{s}\right)}^{2}+O^{\prime }{\hat{\epsilon }}^{s}\mathrm{Tr}\left({\hat{\epsilon }}^{s}\right)+P^{\prime }\mathrm{Tr}\left[{\left({\hat{\epsilon }}^{s}\right)}^{2}\right]\hat{I}+Q^{\prime }{\left[\mathrm{Tr}\left({\hat{\epsilon }}^{s}\right)\right]}^{2}\hat{I} \end{array}$$

where we have used the definitions

(120) $$\begin{array}{ccc} L^{\prime } & = & c+\left(1-c\right)L \end{array}$$

(121) $$\begin{array}{ccc} M^{\prime } & = & (1-c)M \end{array}$$

(122) $$\begin{array}{ccc} N^{\prime } & = & (1-c)N \end{array}$$

(123) $$\begin{array}{ccc} O^{\prime } & = & (1-c)O \end{array}$$

(124) $$\begin{array}{ccc} P^{\prime } & = & (1-c)P \end{array}$$

(125) $$\begin{array}{ccc} Q^{\prime } & = & (1-c)Q \end{array}$$

Starting from Equation (119), we can straightforwardly calculate the quantities $\mathrm{Tr}\langle \hat{\epsilon }\rangle$, ${\langle \hat{\epsilon }\rangle }^{2}$, $\langle \hat{\epsilon }\rangle \mathrm{Tr}\langle \hat{\epsilon }\rangle$, $\mathrm{Tr}\left({\langle \hat{\epsilon }\rangle }^{2}\right)$ and ${\left[Tr\langle \hat{\epsilon }\rangle \right]}^{2}$ in terms of the internal strain ${\hat{\epsilon }}^{s}$ (by using the relation $\mathrm{Tr}(\hat{I})=3$). These set of relations can be written neglecting the terms of order greater than two in ${\hat{\epsilon }}^{s}$, since we are interested in the characterization of the nonlinear elastic properties of the dispersion up to the second order. Therefore, this set of equations can be arranged in a matrix form, as follows

(126) $$\begin{array}{c} \tilde{U}\left[\begin{array}{c} {\hat{\epsilon }}^{s}\\ \mathrm{Tr}\left({\hat{\epsilon }}^{s}\right)\hat{I}\\ {\left({\hat{\epsilon }}^{s}\right)}^{2}\\ {\hat{\epsilon }}^{s}\mathrm{Tr}\left({\hat{\epsilon }}^{s}\right)\\ \mathrm{Tr}\left[{\left({\hat{\epsilon }}^{s}\right)}^{2}\right]\hat{I}\\ {\left[\mathrm{Tr}\left({\hat{\epsilon }}^{s}\right)\right]}^{2}\hat{I} \end{array}\right]=\left[\begin{array}{c} \langle \hat{\epsilon }\rangle \\ \mathrm{Tr}\langle \hat{\epsilon }\rangle \hat{I}\\ {\langle \hat{\epsilon }\rangle }^{2}\\ \langle \hat{\epsilon }\rangle \mathrm{Tr}\langle \hat{\epsilon }\rangle \\ \mathrm{Tr}\left[{\langle \hat{\epsilon }\rangle }^{2}\right]\hat{I}\\ {\left[\mathrm{Tr}\langle \hat{\epsilon }\rangle \right]}^{2}\hat{I} \end{array}\right] \end{array}$$

The elements of the matrix $\tilde{U}$ have been written in terms of the parameters defined in Equations (120)–(125)

(127) $$\begin{array}{c} \tilde{U}=\left[\begin{array}{cccccc} L^{\prime } & M^{\prime } & N^{\prime } & O^{\prime } & P^{\prime } & Q^{\prime }\\ 0 & L^{\prime }+3M^{\prime } & 0 & 0 & N^{\prime }+3P^{\prime } & O^{\prime }+3Q^{\prime }\\ 0 & 0 & {L^{\prime }}^{2} & 2L^{\prime }M^{\prime } & 0 & {M^{\prime }}^{2}\\ 0 & 0 & 0 & L^{\prime }\left(L^{\prime }+3M^{\prime }\right) & 0 & M^{\prime }\left(L^{\prime }+3M^{\prime }\right)\\ 0 & 0 & 0 & 0 & {L^{\prime }}^{2} & M^{\prime }\left(2L^{\prime }+3M^{\prime }\right)\\ 0 & 0 & 0 & 0 & 0 & {\left(L^{\prime }+3M^{\prime }\right)}^{2} \end{array}\right] \end{array}$$

Finally, by using Equation (118) and by inverting Equation (126), we may obtain the matrix form of the complete constitutive relation

(128) $$\begin{array}{c} \langle \hat{T}\rangle =\left({\left[\begin{array}{c} 2\mu_{1}\\ K_{1}-\frac{2}{3}\mu_{1}\\ 0\\ 0\\ 0\\ 0 \end{array}\right]}^{T}+c{\left[\begin{array}{c} 2(\mu_{2}-\mu_{1})\\ K_{2}-K_{1}-\frac{2}{3}\left(\mu_{2}-\mu_{1}\right)\\ A\\ D\\ B\\ C \end{array}\right]}^{T}{\tilde{U}}^{-1}\right)\left[\begin{array}{c} \langle \hat{\epsilon }\rangle \\ \mathrm{Tr}\langle \hat{\epsilon }\rangle \hat{I}\\ {\langle \hat{\epsilon }\rangle }^{2}\\ \langle \hat{\epsilon }\rangle \mathrm{Tr}\langle \hat{\epsilon }\rangle \\ \mathrm{Tr}\left({\langle \hat{\epsilon }\rangle }^{2}\right)\hat{I}\\ {\left[\mathrm{Tr}\langle \hat{\epsilon }\rangle \right]}^{2}\hat{I} \end{array}\right] \end{array}$$

### 11.1. Results

The constitutive equation in the form of Equation (128) can be written in terms of the effective linear and nonlinear elastic moduli as follows

(129) $$\begin{array}{ccc} \langle \hat{T}\rangle & = & 2\mu_{eff}\langle \hat{\epsilon }\rangle +\left(K_{eff}-\frac{2}{3}\mu_{eff}\right)\mathrm{Tr}\langle \hat{\epsilon }\rangle \hat{I}\\ & + & A_{eff}{\langle \hat{\epsilon }\rangle }^{2}+B_{eff}\mathrm{Tr}\left[{\langle \hat{\epsilon }\rangle }^{2}\right]\hat{I}+C_{eff}{\left[\mathrm{Tr}\langle \hat{\epsilon }\rangle \right]}^{2}\hat{I}+D_{eff}\langle \hat{\epsilon }\rangle \mathrm{Tr}\langle \hat{\epsilon }\rangle \end{array}$$

As for the linear elastic moduli, we obtain

(130) $$\begin{array}{ccc} \mu_{eff} & = & \mu_{1}+c\frac{\mu_{2}-\mu_{1}}{L^{\prime }} \end{array}$$

(131) $$\begin{array}{ccc} K_{eff} & = & K_{1}+c\frac{K_{2}-K_{1}}{L^{\prime }+3M^{\prime }} \end{array}$$

and, as for the nonlinear elastic moduli, we have [98,99]

(132) $$\begin{array}{c} A_{eff}=c\frac{A}{{L^{\prime }}^{2}}-2c\frac{N^{\prime }\left(\mu_{2}-\mu_{1}\right)}{{L^{\prime }}^{3}} \end{array}$$

(133) $$\begin{array}{ccc} B_{eff} & = & 2c\frac{\left(N^{\prime }M^{\prime }-L^{\prime }P^{\prime }\right)\left(\mu_{2}-\mu_{1}\right)}{{L^{\prime }}^{3}\left(L^{\prime }+3M^{\prime }\right)}-c\frac{\left(N^{\prime }+3P^{\prime }\right)\left[K_{2}-K_{1}-\frac{2}{3}\left(\mu_{2}-\mu_{1}\right)\right]}{{L^{\prime }}^{2}\left(L^{\prime }+3M^{\prime }\right)}+c\frac{B}{{L^{\prime }}^{2}} \end{array}$$

(134) $$\begin{array}{ccc} C_{eff} & = & \frac{1}{9}\frac{c\left(9C+3B+3D+A\right)}{{\left(L^{\prime }+3M^{\prime }\right)}^{2}}+\frac{1}{9}\frac{c\left(A-3B\right)}{{L^{\prime }}^{2}}\\ & - & \frac{4}{9}\frac{N^{\prime }\left(\mu_{2}-\mu_{1}\right)c}{{L^{\prime }}^{3}}-\frac{1}{9}\frac{c\left(3D+2A\right)}{L^{\prime }\left(L^{\prime }+3M^{\prime }\right)}+\frac{1}{9}\frac{c\left(4N^{\prime }+6O^{\prime }\right)\left(\mu_{2}-\mu_{1}\right)}{{L^{\prime }}^{2}\left(L^{\prime }+3M^{\prime }\right)}\\ & + & \frac{1}{9}\frac{c\left(3N^{\prime }+9P^{\prime }\right)\left(K_{2}-K_{1}\right)}{{L^{\prime }}^{2}\left(L^{\prime }+3M^{\prime }\right)}-\frac{1}{3}\frac{c\left(9Q^{\prime }+3O^{\prime }+3P^{\prime }+N^{\prime }\right)\left(K_{2}-K_{1}\right)}{{\left(L^{\prime }+3M^{\prime }\right)}^{3}} \end{array}$$

(135) $$\begin{array}{ccc} D_{eff} & = & 2c\frac{\left(2N^{\prime }M^{\prime }-L^{\prime }O^{\prime }\right)\left(\mu_{2}-\mu_{1}\right)}{{L^{\prime }}^{3}\left(L^{\prime }+3M^{\prime }\right)}-2c\frac{M^{\prime }A}{{L^{\prime }}^{2}\left(L^{\prime }+3M^{\prime }\right)}+c\frac{D}{L^{\prime }\left(L^{\prime }+3M^{\prime }\right)} \end{array}$$

If we use the definitions of the parameters $L^{\prime }$ and $M^{\prime }$, given in Equations (120) and (121), we obtain for the effective shear and bulk moduli the explicit expressions [98,99]

(136) $$\begin{array}{c} \mu_{eff}=\mu_{1}+c\frac{\mu_{2}-\mu_{1}}{c+\left(1-c\right)\left[1+\frac{6}{5}\left(\frac{\mu_{2}}{\mu_{1}}-1\right)\frac{K_{1}+2\mu_{1}}{3K_{1}+4\mu_{1}}\right]}\;\; \end{array}$$

(137) $$\begin{array}{c} K_{eff}=K_{1}+\frac{\left(3K_{1}+4\mu_{1}\right)\left(K_{2}-K_{1}\right)c}{3K_{2}+4\mu_{1}-3c\left(K_{2}-K_{1}\right)}\;\;\;\;\;\;\;\;\;\;\;\;\;\;\;\;\;\;\;\;\; \end{array}$$

**Figure 19:**
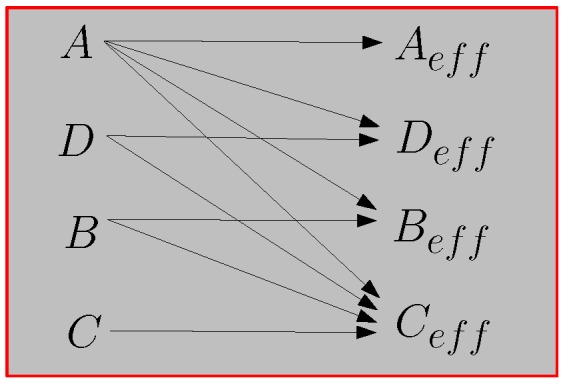
Mixing scheme for the nonlinear modes.

These two expressions are coincident with those obtained for a linear dispersion of elastic spheres [30]. However, they are completed by Equations (132)–(135) in order to characterize the nonlinear properties of the mixture . This set of results fulfils a series of important general properties [98,99]:

1. Equations (132)–(137) are also true for c=1; in this case (very high volume fraction of spheres) the procedure is not expected to be valid but nonetheless the results appears to be exact (if c=1 then $\mu_{eff}=\mu_{2}$, $K_{eff}=K_{2}$, $A_{eff}=A$, $B_{eff}=B$, $C_{eff}=C$, $D_{eff}=D$).
2. The nonlinear elastic moduli *A*, *B*, *C* and *D* influence the effective nonlinear moduli of the composite material following the universal scheme showed in Figure 19. Therefore, there is a complicated mixing of the nonlinear elastic modes induced by the heterogeneity of the structure. The results for the nonlinear effective parameters, obtained with a single coefficient (*A*, *B*, *C* or *D*) different from zero, are reported in Appendix B, in form of series expansions in the volume fraction up to the first order. They are coherent with the scheme shown in Figure 19.

3. If the linear elastic moduli of the matrix and of the spheres are the very same ($K_{1}=K_{2}$ and $\mu_{1}=\mu_{2}$), we simply obtain $K_{eff}=K_{1}$, $\mu_{eff}=\mu_{1}$ and the following special set of results for the nonlinear components
  (138) $$\begin{array}{ccc} A_{eff} & = & cA \end{array}$$
  (139) $$\begin{array}{ccc} B_{eff} & = & cB \end{array}$$
  (140) $$\begin{array}{ccc} C_{eff} & = & cC \end{array}$$
  (141) $$\begin{array}{ccc} D_{eff} & = & cD \end{array}$$
  It means that the nonlinearity of the overall system is simply proportional to the nonlinearity of the spheres.
4. We have developed our procedure under the hypothesis of Cauchy nonlinear elasticity for the spheres embedded in the linear matrix. If we let D=2B we move from the Cauchy elasticity to the Green elasticity, assuming the existence of a strain energy function for the inhomogeneities. It is important to remark that the following property holds: if D=2B then the relation $D_{eff}=2B_{eff}$ is true for the effective nonlinear moduli. It can be verified by direct calculation and it means that our approach is perfectly consistent with the energy balance of the composite material. In other words, we have verified that if a strain energy function exists for the embedded spheres, then an overall strain energy function exists for the whole composite structure.
5. If we consider the special value of the Poisson ratio $\nu_{1}=\nu_{2}=1/5$ (both for the matrix and the spheres) and different values for the Young moduli $E_{1}\neq E_{2}$, we obtain another interesting result: the effective Poisson ratio assume the same value $\nu_{eff}=1/5$, the effective Young modulus $E_{eff}$ assumes the value
  (142) $$\begin{array}{c} E_{eff}=\frac{E_{1}\left(1-c\right)+E_{2}\left(1+c\right)}{E_{1}\left(1+c\right)+E_{2}\left(1-c\right)}E_{1} \end{array}$$
  and the effective nonlinear elastic moduli can be calculated as follows
  (143) $$\begin{array}{c} X_{eff}=\frac{8E_{1}^{3}c}{{\left[E_{1}\left(1+c\right)+E_{2}\left(1-c\right)\right]}^{3}}X \end{array}$$
  where the symbol *X* represents any modulus *A*, *B*, *C* or *D* (the four effective parameters exhibit the same behavior). Therefore, we can say that the special value $\nu_{1}=\nu_{2}=1/5$ uncouples the behavior of the nonlinear elastic modes (described at the point 2), generating a direct correspondence among the nonlinear moduli of the spheres and the effective nonlinear moduli. Furthermore, if we add the condition $E_{1}=E_{2}$, we get back to the point 3. The special value 1/5 for the Poisson ratio comes out in several issues considering a dispersion of spheres. For example, for linear porous materials (with spherical pores) and for linear dispersions of rigid spheres the value 1/5 is a fixed points for the Poisson ratio: if $\nu_{1}=1/5$, then we have $\nu_{eff}=1/5$ for all spheres concentrations [33,100]. Moreover, there is another interesting behavior of the effective Poisson ratio for high volume fraction of pores or rigid spheres: in both cases for c→1 the effective Poisson ratio converges to the fixed value $\nu_{eff}=1/5$, irrespective of the matrix Poisson ratio [33,100,101,102].
6. Finally, we analyze the properties of the dispersion when incompressible material is utilized for the embedded spheres: the constitutive relation Equation (102) describes an incompressible medium in the limit $\lambda_{2}\to \infty$ (or, equivalently, $K_{2}\to \infty$ since $K_{2}=\lambda_{2}+2\mu_{2}/3$); by inverting Equation (102), writing the strain tensor in terms of the stress tensor and performing such a limit, we obtain (up to the second order)
  (144) $$\begin{array}{ccc} {\hat{\epsilon }}^{s} & = & \frac{1}{2\mu_{2}}{\hat{T}}^{s}-\frac{1}{6\mu_{2}}\mathrm{Tr}\left({\hat{T}}^{s}\right)\hat{I}-\frac{A}{8\mu_{2}^{3}}{\left({\hat{T}}^{s}\right)}^{2}\\ & + & \frac{A}{24\mu_{2}^{3}}\mathrm{Tr}\left[{\left({\hat{T}}^{s}\right)}^{2}\right]\hat{I}-\frac{A}{36\mu_{2}^{3}}{\left[\mathrm{Tr}\left({\hat{T}}^{s}\right)\right]}^{2}\hat{I}+\frac{A}{12\mu_{2}^{3}}{\hat{T}}^{s}\mathrm{Tr}\left({\hat{T}}^{s}\right) \end{array}$$
  which describes a nonlinear isotropic and incompressible material. We remark that only the nonlinear modulus *A* intervenes in defining such a constitutive equation and that Equation (144) imposes $\mathrm{Tr}\left({\hat{\epsilon }}^{s}\right)=0$, as requested by the incompressibility. In this limiting condition, as for the effective linear moduli, we observe that Equation (136) for $\mu_{eff}$ remains unchanged and Equation (137) leads to
  (145) $$\begin{array}{c} K_{eff}=K_{1}+\left(K_{1}+\frac{4}{3}\mu_{1}\right)\frac{c}{1-c} \end{array}$$
  On the other hand, the nonlinear elastic moduli have been eventually found as
  (146) $$\begin{array}{ccc} A_{eff} & = & 125A\theta \end{array}$$
  (147) $$\begin{array}{ccc} B_{eff} & = & -\frac{125}{3}A\theta \end{array}$$
  (148) $$\begin{array}{ccc} C_{eff} & = & \frac{250}{9}A\theta \end{array}$$
  (149) $$\begin{array}{ccc} D_{eff} & = & -\frac{250}{3}A\theta \end{array}$$
  where
  (150) $$\begin{array}{ccc} \theta & = & \frac{c{\left(3K_{1}+4\mu_{1}\right)}^{3}\mu_{1}^{3}}{\psi^{3}} \end{array}$$
  (151) $$\begin{array}{ccc} \psi & = & 6\left(K_{1}+2\mu_{1}\right)\left[c\mu_{1}+\left(1-c\right)\mu_{2}\right]+\mu_{1}\left(9K_{1}+8\mu_{1}\right) \end{array}$$
  One can observe that, as expected, the effective nonlinear elastic moduli depend only on the modulus *A* describing the nonlinearity of the spheres, as shown in Equation (144). Moreover, we remark that a single modulus *A* for the spheres can generate four different effective nonlinear moduli, as predicted by the scheme in Figure 19.

**Figure 20:**
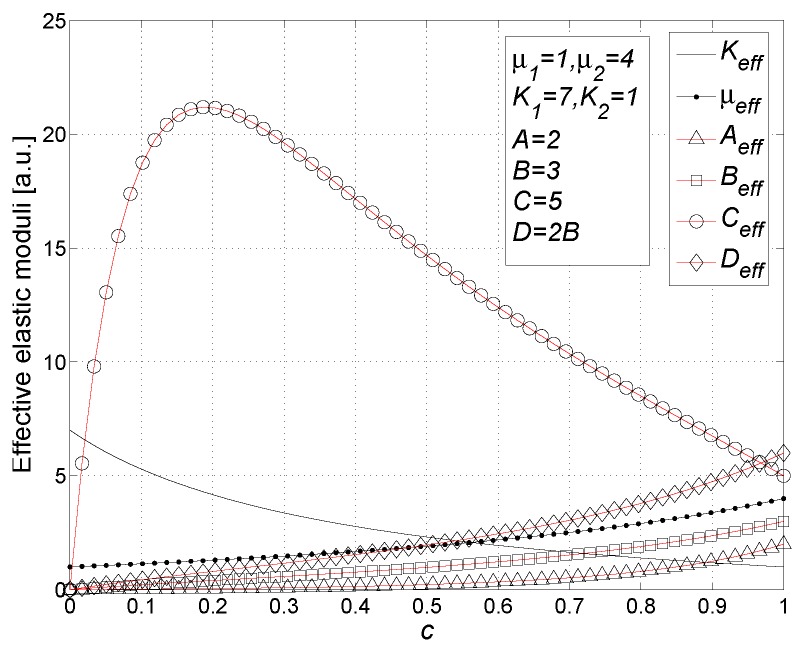
Linear and nonlinear effective elastic moduli of a dispersion of spheres in terms of the volume fraction *c*. We have used the values $\mu_{1}=1,\mu_{2}=4,K_{1}=7,K_{2}=1,A=2,B=3,C=5,D=2B$ in arbitrary units.

**Figure 21:**
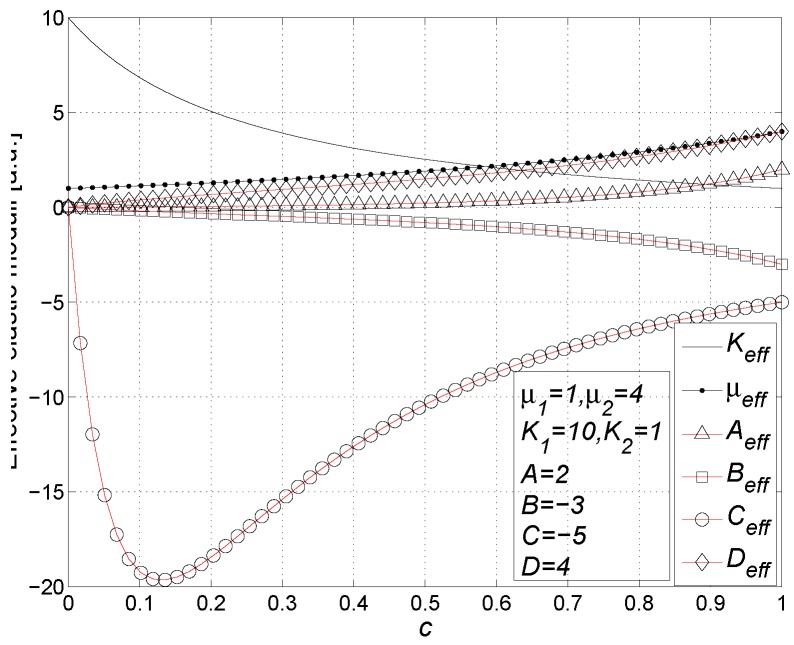
Linear and nonlinear effective elastic moduli of a dispersion of spheres in terms of the volume fraction *c*. We have used the values $\mu_{1}=1,\mu_{2}=4,K_{1}=10,K_{2}=1,A=2,B=-3,C=-5,D=4$ in arbitrary units.

To conclude, we present some numerical results obtained by the implementation of Equations (132)–(137). In Figure 20 we have considered Green nonlinear elasticity and the mixture parameters: $\mu_{1}=1,\mu_{2}=4,K_{1}=7,K_{2}=1,A=2,B=3,C=5,D=2B$ in arbitrary units. In Figure 21 we have considered Cauchy nonlinear elasticity and the mixture parameters: $\mu_{1}=1,\mu_{2}=4,K_{1}=10,K_{2}=1,A=2,B=-3,C=-5,D=4$ in arbitrary units. The results have been presented in terms of the volume fraction *c* of the spheres. In both cases we may observe a consistent amplification of the nonlinear effective modulus $C_{eff}$. We have verified that such a phenomenon is always exhibited when

$K_{1}\gg K_{2}$ (i.e., when the matrix is much more incompressible than the spheres) and that the higher values of $C_{eff}$ appear for small values of the volume fraction *c*, belonging to the range of applicability of the present theory.

As it is well known, simple limitations for the values of the linear effective moduli are well established

(152) $$\begin{array}{ccc} \frac{1}{\frac{1-c}{K_{1}}+\frac{c}{K_{2}}}≦ & K_{eff} & ≦\left(1-c\right)K_{1}+cK_{2} \end{array}$$

(153) $$\begin{array}{ccc} \frac{1}{\frac{1-c}{\mu_{1}}+\frac{c}{\mu_{2}}}≦ & \mu_{eff} & ≦\left(1-c\right)\mu_{1}+c\mu_{2} \end{array}$$

The lower bounds in Equations (152) and (153) are referred to as the Voigt bounds, and the upper bounds are designated as the Reuss bounds [29]. Unfortunately, these bounds are of no practical value, but more refined bounds, with realistic applications, have been derived by Hashin and Shtrikman [4]. From our numerical results, shown in Figure 20 and Figure 21, we may observe that the nonlinear properties, contrary to the linear ones, are not bounded by some given values and in certain conditions exhibit a strong amplification, which leads to nonlinear effective moduli much greater than those of the constituents. This point is important in the topic of designing materials with desired properties and functions.

## 12. Elastic Dispersion of Parallel Nonlinear Cylindrical Inhomogeneities

**Figure 22:**
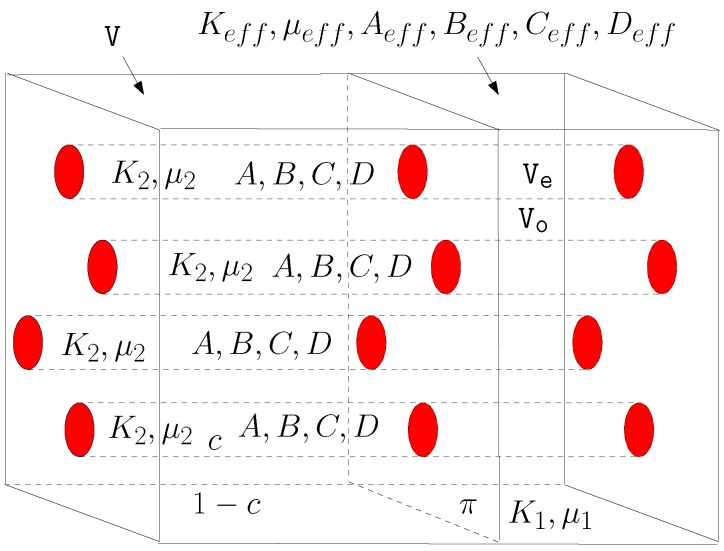
Scheme of a dispersion of nonlinear parallel cylinders embedded in a linear matrix.

We now take into consideration an assembly of parallel cylinders, as represented in Figure 22, described by an arbitrary Cauchy constitutive relation [see Equation (102)]. As before, when needed, we can easily move to the Green elasticity by assuming D=2B. The cylindrical inhomogeneities are randomly embedded in a linear matrix with elastic moduli $K_{1}$ and $\mu_{1}$. This is a simple but complete way for modeling a nonlinear fibrous material. In earlier works the linear analysis for a parallel distribution of fibers has been developed by means of the Eshelby methodology and of the differential effective medium theory [32,103]. Moreover, the mechanical response of elastic and inelastic fiber-strengthened materials has been investigated, also with self-consistent models [104,105,106]. Here, in order to deal with the nonlinear properties, we suppose that the volume fraction *c* of the embedded phase is small (dilute dispersion). It means that each cylinder can be considered isolated in the space (not interacting with

other inhomogeneities) and subject to the same external loading. In order to simplify the modeling and considering that the system shows a transverse isotropic symmetry (otherwise said uniaxial symmetry), we assume the plane strain condition on an arbitrary plane *π* (see Figure 22) orthogonal to the cylinders. It means that we are dealing with a problem belonging to the two-dimensional elasticity. Moreover, in plain strain condition, it is a common choice to introduce the two dimensional elastic moduli $\mu^{2D}=\mu$ and $K^{2D}=K+\mu /3$, where *K* and *μ* are the customarily used three-dimensional moduli [103]. Throughout this section we indicate for brevity *K* and *μ* alluding to the two-dimensional version of the elastic moduli. It means that the linear matrix is described by

(154) $$\begin{array}{ccc} \hat{T} & = & 2\mu_{1}\hat{\epsilon }+\left(K_{1}-\mu_{1}\right)\mathrm{Tr}\left(\hat{\epsilon }\right)\hat{I} \end{array}$$

and the cylindrical inhomogeneities are described by the Cauchy constitutive relation

(155) $$\begin{array}{c} {\hat{T}}^{s}=2\mu_{2}{\hat{\epsilon }}^{s}+\left(K_{2}-\mu_{2}\right)\mathrm{Tr}\left({\hat{\epsilon }}^{s}\right)\hat{I}+A{\left({\hat{\epsilon }}^{s}\right)}^{2}+B\mathrm{Tr}\left[{\left({\hat{\epsilon }}^{s}\right)}^{2}\right]\hat{I}+C{\left[\mathrm{Tr}\left({\hat{\epsilon }}^{s}\right)\right]}^{2}\hat{I}+D{\hat{\epsilon }}^{s}\mathrm{Tr}\left({\hat{\epsilon }}^{s}\right) \end{array}$$

where any strain or stress tensor is represented by a square matrix of order two, working in the framework of the two-dimensional elasticity. Now, we remark that Equation (99) or, equivalently, Equation (103) are correct for any geometry and, therefore, they can be directly used in the present analysis. Nevertheless, in order to use Equation (103) we need to consider some ingredients: the result of the application of the compliance tensor of the matrix on the stress tensor ${\hat{T}}^{s}$ can be written as

(156) $${\left({\hat{C}}^{\left(1\right)}\right)}^{-1}{\hat{T}}^{s}=\frac{1}{2\mu_{1}}{\hat{T}}^{s}-\frac{K_{1}-\mu_{1}}{4\mu_{1}K_{1}}\mathrm{Tr}\left({\hat{T}}^{s}\right)\hat{I}$$

Moreover, the effect of the Eshelby tensor $\hat{S}$ for a cylinder over an arbitrary strain tensor ${\hat{\epsilon }}^{s}$ is given by [84]

(157) $$\begin{array}{c} \hat{S}{\hat{\epsilon }}^{s}=\frac{1}{2}\frac{K_{1}+2\mu_{1}}{K_{1}+\mu_{1}}{\hat{\epsilon }}^{s}+\frac{1}{4}\frac{K_{1}-2\mu_{1}}{K_{1}+\mu_{1}}\mathrm{Tr}\left({\hat{\epsilon }}^{s}\right)\hat{I} \end{array}$$

Now, in order to obtain a single equation for the internal strain ${\hat{\epsilon }}^{s}$, we can substitute Equations (155), (156) and (157) in the starting Equation (103). A tedious calculation leads to the equation [99]

(158) $$\begin{array}{ccc} L{\hat{\epsilon }}^{s} & + & M\mathrm{Tr}\left({\hat{\epsilon }}^{s}\right)\hat{I}+N{\left({\hat{\epsilon }}^{s}\right)}^{2}+O{\hat{\epsilon }}^{s}\mathrm{Tr}\left({\hat{\epsilon }}^{s}\right)+P\mathrm{Tr}\left[{\left({\hat{\epsilon }}^{s}\right)}^{2}\right]\hat{I}+Q{\left[\mathrm{Tr}\left({\hat{\epsilon }}^{s}\right)\right]}^{2}\hat{I}={\hat{\epsilon }}^{\infty } \end{array}$$

which completely defines the internal strain induced in a nonlinear cylinder by the uniform externally applied deformation ${\hat{\epsilon }}^{\infty }$. The parameters L,M,N,O,P and *Q* have been defined as

(159) $$\begin{array}{ccc} L & = & 1+\frac{1}{2}\frac{K_{1}+2\mu_{1}}{K_{1}+\mu_{1}}\left(\frac{\mu_{2}}{\mu_{1}}-1\right) \end{array}$$

(160) $$\begin{array}{ccc} M & = & \frac{1}{4\left(K_{1}+\mu_{1}\right)}\left[2K_{2}-K_{1}\left(1+\frac{\mu_{2}}{\mu_{1}}\right)-2\left(\mu_{2}-\mu_{1}\right)\right] \end{array}$$

(161) $$\begin{array}{ccc} N & = & \frac{A}{4\mu_{1}}\frac{K_{1}+2\mu_{1}}{K_{1}+\mu_{1}} \end{array}$$

(162) $$\begin{array}{ccc} O & = & \frac{D}{4\mu_{1}}\frac{K_{1}+2\mu_{1}}{K_{1}+\mu_{1}} \end{array}$$

(163) $$\begin{array}{ccc} P & = & \frac{1}{8\left(K_{1}+\mu_{1}\right)}\left(4B-A\frac{K_{1}}{\mu_{1}}\right) \end{array}$$

(164) $$\begin{array}{ccc} Q & = & \frac{1}{8\left(K_{1}+\mu_{1}\right)}\left(4C-D\frac{K_{1}}{\mu_{1}}\right) \end{array}$$

We follow a procedure similar to that described in Section 4. We use again Equations (116) and (119) for the average values of the stress and the strain over the whole composite material. At this point, starting from Equation (119), we obtain the system given in Equation (126) (by using the two-dimensional relation $\mathrm{Tr}(\hat{I})=2$). It is defined by the matrix $\tilde{U}$ where the elements depend on the parameters defined in Equations (120)–(125) and calculated by means of Equations (159)–(164)

(165) $$\begin{array}{c} \tilde{U}=\left[\begin{array}{cccccc} L^{\prime } & M^{\prime } & N^{\prime } & O^{\prime } & P^{\prime } & Q^{\prime }\\ 0 & L^{\prime }+2M^{\prime } & 0 & 0 & N^{\prime }+2P^{\prime } & O^{\prime }+2Q^{\prime }\\ 0 & 0 & {L^{\prime }}^{2} & 2L^{\prime }M^{\prime } & 0 & {M^{\prime }}^{2}\\ 0 & 0 & 0 & L^{\prime }\left(L^{\prime }+2M^{\prime }\right) & 0 & M^{\prime }\left(L^{\prime }+2M^{\prime }\right)\\ 0 & 0 & 0 & 0 & {L^{\prime }}^{2} & 2M^{\prime }\left(L^{\prime }+M^{\prime }\right)\\ 0 & 0 & 0 & 0 & 0 & {\left(L^{\prime }+2M^{\prime }\right)}^{2} \end{array}\right] \end{array}$$

Finally, by inverting Equation (126), we may obtain the matrix form of the complete constitutive relation

(166) $$\begin{array}{c} \langle \hat{T}\rangle =\left({\left[\begin{array}{c} 2\mu_{1}\\ K_{1}-\mu_{1}\\ 0\\ 0\\ 0\\ 0 \end{array}\right]}^{T}+c{\left[\begin{array}{c} 2(\mu_{2}-\mu_{1})\\ K_{2}-K_{1}-\left(\mu_{2}-\mu_{1}\right)\\ A\\ D\\ B\\ C \end{array}\right]}^{T}{\tilde{U}}^{-1}\right)\left[\begin{array}{c} \langle \hat{\epsilon }\rangle \\ \mathrm{Tr}\langle \hat{\epsilon }\rangle \hat{I}\\ {\langle \hat{\epsilon }\rangle }^{2}\\ \langle \hat{\epsilon }\rangle \mathrm{Tr}\langle \hat{\epsilon }\rangle \\ \mathrm{Tr}\left({\langle \hat{\epsilon }\rangle }^{2}\right)\hat{I}\\ {\left[\mathrm{Tr}\langle \hat{\epsilon }\rangle \right]}^{2}\hat{I} \end{array}\right] \end{array}$$

### 12.1. Results

The constitutive equation in the form of Equation (166) can be written in terms of the effective linear and nonlinear elastic moduli as follows

(167) $$\begin{array}{ccc} \langle \hat{T}\rangle & = & 2\mu_{eff}\langle \hat{\epsilon }\rangle +\left(K_{eff}-\mu_{eff}\right)\mathrm{Tr}\langle \hat{\epsilon }\rangle \hat{I}\\ & + & A_{eff}{\langle \hat{\epsilon }\rangle }^{2}+B_{eff}\mathrm{Tr}\left[{\langle \hat{\epsilon }\rangle }^{2}\right]\hat{I}+C_{eff}{\left[\mathrm{Tr}\langle \hat{\epsilon }\rangle \right]}^{2}\hat{I}+D_{eff}\langle \hat{\epsilon }\rangle \mathrm{Tr}\langle \hat{\epsilon }\rangle \end{array}$$

As for the linear elastic moduli, we obtain [99]

(168) $$\begin{array}{ccc} \mu_{eff} & = & \mu_{1}+c\frac{\mu_{2}-\mu_{1}}{L^{\prime }}=\mu_{1}+c\frac{\mu_{2}-\mu_{1}}{c+\left(1-c\right)\left[1+\frac{1}{2}\left(\frac{\mu_{2}}{\mu_{1}}-1\right)\frac{K_{1}+2\mu_{1}}{K_{1}+\mu_{1}}\right]} \end{array}$$

(169) $$\begin{array}{ccc} K_{eff} & = & K_{1}+c\frac{K_{2}-K_{1}}{L^{\prime }+2M^{\prime }}=K_{1}+c\frac{K_{2}-K_{1}}{c+\left(1-c\right)\frac{\mu_{1}+K_{2}}{\mu_{1}+K_{1}}} \end{array}$$

It is important to remember that the bulk modulus $K_{eff}$ represents the two-dimensional version, as above defined. Moreover, the two linear results given in Equations (168) and (169) are perfectly coincident with earlier literature [107]. As for the effective nonlinear elastic moduli, we have the following final results [99]

(170) $$\begin{array}{c} A_{eff}=\frac{Ac}{{L^{\prime }}^{2}}-2c\frac{N^{\prime }\left(\mu_{2}-\mu_{1}\right)}{{L^{\prime }}^{3}} \end{array}$$

(171) $$\begin{array}{ccc} B_{eff} & = & \frac{c\left[N^{\prime }\left(\mu_{2}-\mu_{1}\right)+BL^{\prime }\right]}{{L^{\prime }}^{3}}-\frac{c\left(2P^{\prime }+N^{\prime }\right)\left(K_{2}-K_{1}\right)}{{L^{\prime }}^{2}\left(L^{\prime }+2M^{\prime }\right)} \end{array}$$

(172) $$\begin{array}{ccc} C_{eff} & = & c\frac{4C+2B+2D+A}{4{\left(L^{\prime }+2M^{\prime }\right)}^{2}}+c\frac{A-2B}{4{L^{\prime }}^{2}}+c\frac{2\left(O^{\prime }+N^{\prime }\right)\left(\mu_{2}-\mu_{1}\right)+\left(2P^{\prime }+N^{\prime }\right)\left(K_{2}-K_{1}\right)}{2{L^{\prime }}^{2}\left(L^{\prime }+2M^{\prime }\right)}\\ & - & \frac{c\left(2P^{\prime }+N^{\prime }+4Q^{\prime }+2O^{\prime }\right)\left(K_{2}-K_{1}\right)}{2{\left(L^{\prime }+2M^{\prime }\right)}^{3}}-\frac{cN^{\prime }\left(\mu_{2}-\mu_{1}\right)}{{L^{\prime }}^{3}}-c\frac{A+D}{2L^{\prime }\;\left(L^{\prime }+2M^{\prime }\right)} \end{array}$$

(173) $$\begin{array}{ccc} D_{eff} & = & 2\frac{\left(2N^{\prime }M^{\prime }-L^{\prime }O^{\prime }\right)\left(\mu_{2}-\mu_{1}\right)c}{{L^{\prime }}^{3}\left(L^{\prime }+2M^{\prime }\right)}-2c\frac{M^{\prime }A}{{L^{\prime }}^{2}\left(L^{\prime }+2M^{\prime }\right)}+\frac{cD}{L^{\prime }\left(L^{\prime }+2M^{\prime }\right)} \end{array}$$

They represent the complete nonlinear characterization of the random dispersion of parallel cylinders. It is interesting to observe that all the properties described in the previous section for the dispersion of spheres (points 1–6) can be easily verified also for the present case [99]. In particular, the scheme represented in Figure 19 remains valid. In Appendix C we have reported the explicit results giving the first order expansions of the nonlinear elastic moduli with respect to the volume fraction, corresponding to the simple cases where only one nonlinear parameter of the cylinders is different from zero. We analyze the case corresponding to the point 5 of the previous section: we consider the special value of the three-dimensional Poisson ratio $\nu_{1}=\nu_{2}=1/4$ (corresponding to the two-dimensional Poisson ratio $\nu_{2D}=\nu_{3D}/\left(1-\nu_{3D}\right)=1/3$ [103]) and different values for the three-dimensional Young moduli $E_{1}\neq E_{2}$. In this case, the effective 3D Poisson ratio assume the value $\nu_{eff}=1/4$ and the effective 3D Young modulus $E_{eff}$ assumes the value

(174) $$\begin{array}{c} E_{eff}=\frac{E_{1}\left(1-c\right)+E_{2}\left(2+c\right)}{E_{1}\left(1+2c\right)+2E_{2}\left(1-c\right)}E_{1} \end{array}$$

Moreover, the effective nonlinear elastic moduli can be calculated as follows

(175) $$\begin{array}{c} X_{eff}=\frac{27E_{1}^{3}c}{{\left[E_{1}\left(1+2c\right)+2E_{2}\left(1-c\right)\right]}^{3}}X \end{array}$$

where the symbol *X* represents any modulus *A*, *B*, *C* or *D* (the four effective parameters exhibit the same behavior). Therefore, as before, we can say that the special value $\nu_{1}=\nu_{2}=1/4$ uncouples the behavior of the nonlinear elastic modes, generating a direct correspondence among the nonlinear moduli of the spheres and the effective nonlinear moduli.

**Figure 23:**
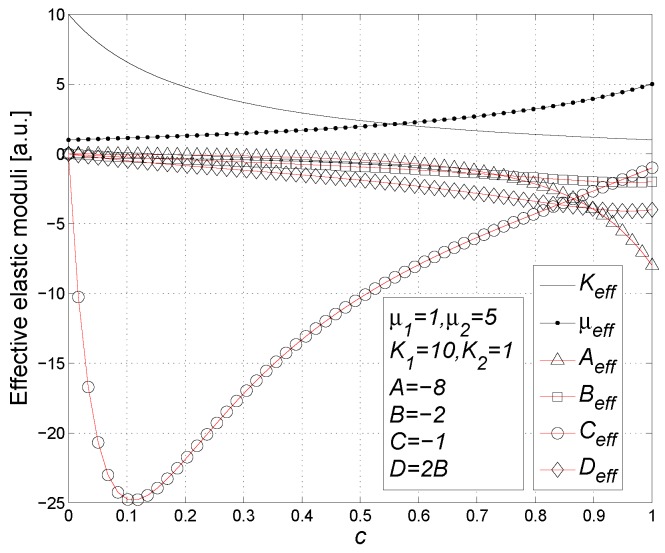
Linear and nonlinear effective elastic moduli for a dispersion of cylinders in terms of the volume fraction *c*. We have used the values $\mu_{1}=1,\mu_{2}=5,K_{1}=10,K_{2}=1,A=-8,B=-2,C=-1,D=2B$ in arbitrary units.

**Figure 24:**
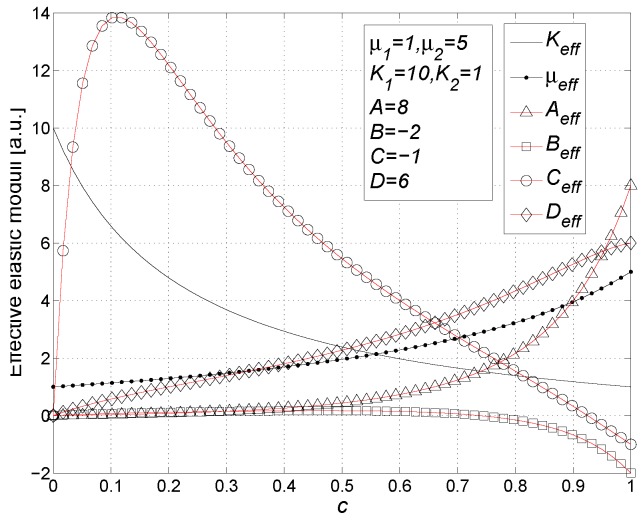
Linear and nonlinear effective elastic moduli for a dispersion of cylinders in terms of the volume fraction *c*. We have used the values $\mu_{1}=1,\mu_{2}=5,K_{1}=10,K_{2}=1,A=8,B=-2,C=-1,D=6$ in arbitrary units.

Finally, we have numerically implemented Equations (168)–(173) in order to show some explicit results. In Figure 23 we have considered Green nonlinear elasticity and the mixture parameters: $\mu_{1}=1,\mu_{2}=5,K_{1}=10,K_{2}=1,A=-8,B=-2,C=-1,D=2B$ in arbitrary units. In Figure 24 we have considered Cauchy nonlinear elasticity and the mixture parameters: $\mu_{1}=1,\mu_{2}=5,K_{1}=10,K_{2}=1,A=8,B=-2,C=-1,D=6$ in arbitrary units. As in the previous section, we may observe a consistent amplification of the nonlinear effective modulus $C_{eff}$. We have also verified that such a phenomenon is exhibited when $K_{1}\gg K_{2}$ (i.e., when the matrix is much more incompressible than the spheres) and that the higher values of $C_{eff}$ appear for small values of the volume fraction *c*, belonging to the range of applicability of the present theory.

## 13. Conclusions

In the previous sections we have considered the linear and nonlinear elastic behavior of a composite material. In particular we have taken into account a dispersion of isotropic nonlinear inhomogeneities

(spheres or parallel cylinders) embedded into a linear isotropic host matrix. The nonlinearity of the inhomogeneities has been described either by the Cauchy model (four parameters) or by the energy-based Green approach (three parameters).

We have introduced two simplifying hypotheses: the small volume fraction of the embedded particles and the small deformations of the whole solid body. Nevertheless, we have described useful results both for analyzing the mechanical properties of a given heterogeneous structure and for designing a composite material with a desired linear and nonlinear elastic behavior.

The main concept introduced to homogenize the heterogeneous structures is a generalization of the linear Eshelby methodology developed for extending its applicability to nonlinear materials. This approach has been analytically applied to perform a linear and nonlinear micromechanical averaging in the composite structure and, therefore, to develop a complete homogenizing procedure yielding the mechanical behavior of the solid body at the macro-scale.

As for the linear properties, we have obtained a series of results in perfect agreement with earlier researches on this subject. This point can be considered as a check of the mathematical procedure. As for the nonlinear properties, firstly, we have obtained the expressions of the four effective elastic moduli of the composite medium with inhomogeneities described by the Cauchy constitutive equations, which represent the less restrictive way to model the nonlinear elasticity. Then, we have considered, as a particular case, the Green elasticity to describe the nonlinear behavior of the particles. In this case we have verified that if a strain energy function exists for the inhomogeneities, then an overall strain energy function exists for the whole composite structure. This point confirms the perfect coherence between our micromechanical averaging procedure and the thermodynamics of the composite material.

Moreover, we have observed that the nonlinear effective elastic moduli, contrary to the linear ones, are not subject to specific bounds that limit their values when the behaviors of the constituents are chosen. We have indeed found some strong amplifications of the nonlinear behavior in certain given

conditions. More specifically, for example, we have observed that the nonlinear modulus $C_{eff}$ can assume values much greater than *C* if the matrix is much more incompressible than the inhomogeneities. This is a crucial point that can be applied in analyzing and designing composite materials with a given microstructure.

Finally, some special values of the Poisson ratio of the materials have been found in order to obtain a direct correspondence among the nonlinear moduli of the inhomogeneities and the effective moduli of the composite structure. It means that, under the above conditions, we can realize a perfect scaling of the nonlinear properties (see Equation (143) or (175)) modulated by the ratio $E_{1}/E_{2}$ between the Young moduli of the constituents.

## Figures and Tables

**Figure 25:**
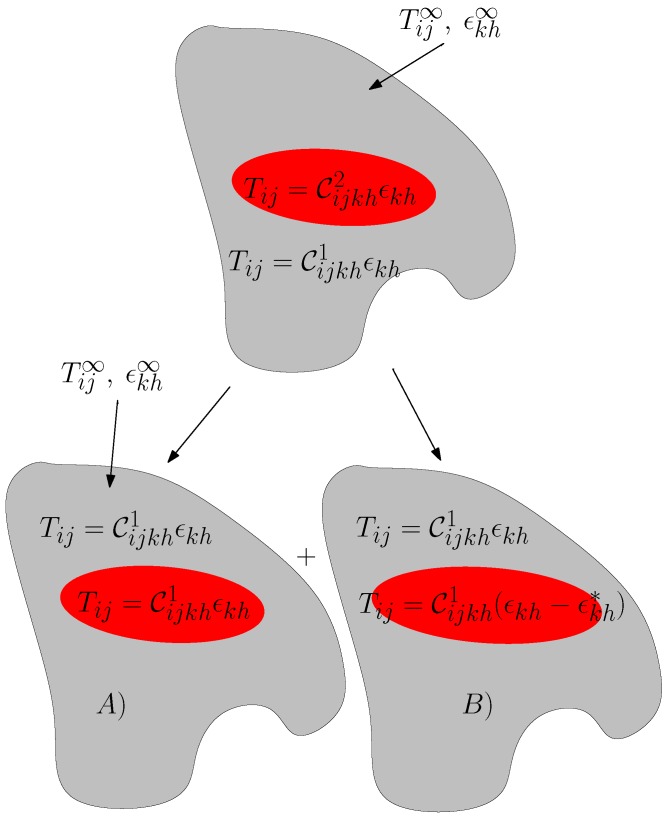
Scheme of an ellipsoidal inhomogeneity and the Eshelby equivalence principle.

**Figure 26:**
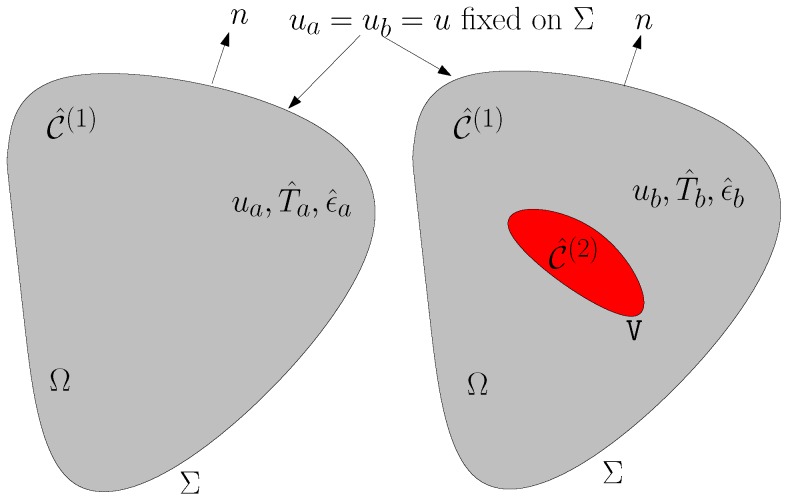
Schemes of an homogeneous region and an heterogeneous one with an inhomogeneity V. The boundary conditions prescribe the same displacement on the external surface.

## Appendix A Symmetry and Positive Definiteness of the Tensor ${\hat{C}}^{\left(1\right)}[{\hat{S}}^{-1}-\hat{I}]$

We briefly outline the concepts of inclusion and linear inhomogeneity in order to present the adopted notation and to recall the most important equations of the Eshelby theory [84,93,94].

### Concept of inclusion

We consider an infinite medium with stiffness tensor ${\hat{C}}^{\left(1\right)}$; moreover, we consider an embedded ellipsoidal inclusion V described by the constitutive equation $\hat{T}={\hat{C}}^{\left(1\right)}\left(\hat{\epsilon }-{\hat{\epsilon }}^{*}\right)$. The strain ${\hat{\epsilon }}^{*}$ is called eigenstrain (or stress-free strain). In these conditions the following relations describe the strain inside and outside the inclusion [84]

(176) $$\begin{array}{c} \hat{\epsilon }\left(\vec{x}\right)=\left\{\begin{array}{cc} \hat{S}{\hat{\epsilon }}^{*} & \;\mathrm{if}\;\vec{x}\in V\\ {\hat{S}}^{\infty }\left(\vec{x}\right){\hat{\epsilon }}^{*} & \;\mathrm{if}\;\vec{x}\notin V \end{array}\right. \end{array}$$

where $\hat{S}$ is the internal Eshelby tensor and ${\hat{S}}^{\infty }$ is the external Eshelby tensor.

### Concept of inhomogeneity

We now consider an infinite medium with stiffness tensor ${\hat{C}}^{\left(1\right)}$ in ${ℜ}^{3}\setminus V$ (matrix) and ${\hat{C}}^{\left(2\right)}$ in the ellipsoidal region V (inhomogeneity). We remotely load the system with a uniform strain ${\hat{\epsilon }}^{\infty }$ or, equivalently, with the uniform stress ${\hat{T}}^{\infty }$. Of course we have ${\hat{T}}^{\infty }={\hat{C}}^{\left(1\right)}{\hat{\epsilon }}^{\infty }$. This configuration can be analyzed by means of the Eshelby equivalence principle [93]. The system can be described by the superimposition of two simpler cases (see Figure 25) [84]. The first situation *A* concerns a medium with stiffness ${\hat{C}}^{\left(1\right)}$

(without inclusions or inhomogeneities) uniformly deformed by means of the remote loads ${\hat{\epsilon }}^{\infty }$ or $T^{\infty }$. The second situation *B* is represented by an inclusion embedded in a medium, characterized everywhere by ${\hat{C}}^{\left(1\right)}$ and having an eigenstrain ${\hat{\epsilon }}^{*}$ in V. The situation *B* is without remote loads. The eigenstrain must be imposed searching for the equivalence between the original inhomogeneity problem and the superimposition A+B. The following relation hold on inside the region V (*s* means inside V)

(177) $$\begin{array}{ccc} {\hat{\epsilon }}^{s} & = & {\hat{\epsilon }}^{A,s}+{\hat{\epsilon }}^{B,s}={\hat{\epsilon }}^{\infty }+\hat{S}{\hat{\epsilon }}^{*}\\ {\hat{T}}^{s} & = & {\hat{T}}^{A,s}+{\hat{T}}^{B,s}={\hat{C}}^{\left(1\right)}{\hat{\epsilon }}^{\infty }+{\hat{C}}^{\left(1\right)}\left({\hat{\epsilon }}^{B,s}-{\hat{\epsilon }}^{*}\right)={\hat{C}}^{\left(1\right)}{\hat{\epsilon }}^{\infty }+{\hat{C}}^{\left(1\right)}\left(\hat{S}{\hat{\epsilon }}^{*}-{\hat{\epsilon }}^{*}\right) \end{array}$$

In the inhomogeneity we have ${\hat{T}}^{s}={\hat{C}}^{\left(2\right)}{\hat{\epsilon }}^{s}$ and therefore

(178) $$\underset{{\hat{T}}^{s}}{\underset{︸}{{\hat{C}}^{\left(1\right)}{\hat{\epsilon }}^{\infty }+{\hat{C}}^{\left(1\right)}\left(\hat{S}{\hat{\epsilon }}^{*}-{\hat{\epsilon }}^{*}\right)}}={\hat{C}}^{\left(2\right)}\underset{{\hat{\epsilon }}^{s}}{\underset{︸}{\left({\hat{\epsilon }}^{\infty }+\hat{S}{\hat{\epsilon }}^{*}\right)}}$$

The following relations can be finally obtained for the eigenstrain and for the actual strain in V

(179) $$\begin{array}{ccc} {\hat{\epsilon }}^{*} & = & {\left[{\left(\hat{I}-{\left({\hat{C}}^{\left(1\right)}\right)}^{-1}{\hat{C}}^{\left(2\right)}\right)}^{-1}-\hat{S}\right]}^{-1}{\hat{\epsilon }}^{\infty } \end{array}$$

(180) $$\begin{array}{ccc} {\hat{\epsilon }}^{s} & = & {\left(\hat{I}-{\left({\hat{C}}^{\left(1\right)}\right)}^{-1}{\hat{C}}^{\left(2\right)}\right)}^{-1}{\hat{\epsilon }}^{*} \end{array}$$

(181) $$\begin{array}{ccc} {\hat{\epsilon }}^{s} & = & {\left[\hat{I}-\hat{S}\left(\hat{I}-{\left({\hat{C}}^{\left(1\right)}\right)}^{-1}{\hat{C}}^{\left(2\right)}\right)\right]}^{-1}{\hat{\epsilon }}^{\infty } \end{array}$$

If ${\hat{C}}^{\left(2\right)}=0$ (void) we obtain

(182) $$\begin{array}{cc} {\hat{\epsilon }}^{*}={\hat{\epsilon }}^{s}= & {\left[\hat{I}-\hat{S}\right]}^{-1}{\hat{\epsilon }}^{\infty } \end{array}$$

### Lemma: The tensor ${\hat{C}}^{\left(1\right)}\left[{\hat{S}}^{-1}-\hat{I}\right]$ is symmetric

We consider the same inclusion V with two different values for the eigenstrain ${\hat{\epsilon }}^{*}$ and ${\hat{\epsilon }}^{**}$ embedded in the material defined by ${\hat{C}}^{\left(1\right)}$. The symmetry of the tensor can be established by means of a revised version of the Betti’s reciprocal theorem [85]. We define ${\hat{T}}^{*}={\hat{C}}^{\left(1\right)}{\hat{\epsilon }}^{*}$ and ${\hat{T}}^{**}={\hat{C}}^{\left(1\right)}{\hat{\epsilon }}^{**}$. The first situation is described by the fields ${\hat{T}}^{\prime },{\hat{\epsilon }}^{\prime },{\vec{u}}^{\prime }$ and the second one by ${\hat{T}}^{\prime \prime },{\hat{\epsilon }}^{\prime \prime },{\vec{u}}^{\prime \prime }$ everywhere in the space. The preliminary symmetry of the tensor $\hat{S}{\left[{\hat{C}}^{\left(1\right)}\right]}^{-1}$ is proved. We begin by considering the following relation (V is the inclusion volume , Σ its boundary and $\vec{n}$ its external normal unit vector)

(183) $$\begin{array}{ccc} V{\hat{T}}^{*}\hat{S}{\left[{\hat{C}}^{\left(1\right)}\right]}^{-1}{\hat{T}}^{**} & = & V{\hat{T}}^{*}\hat{S}{\hat{\epsilon }}^{**}=V{\hat{T}}^{*}{\hat{\epsilon }}^{\prime \prime }\\ & = & {\hat{T}}^{*}\int_{V}{\hat{\epsilon }}^{\prime \prime }dv={\hat{T}}^{*}\int_{V}\frac{\partial {\vec{u}}^{\prime \prime }}{\partial \vec{x}}dv\\ & = & {\hat{T}}^{*}\int_{\Sigma }{\vec{u}}^{\prime \prime }\vec{n}dS={\hat{C}}^{\left(1\right)}{\hat{\epsilon }}^{*}\int_{\Sigma }{\vec{u}}^{\prime \prime }\vec{n}dS \end{array}$$

At the interface Σ we have ${\hat{T}}^{\prime }\vec{n}{{|}_{\Sigma^{-}}={\hat{T}}^{\prime }\vec{n}|}_{\Sigma^{+}}$ (sign + indicates the external side of Σ and sign − indicates its internal side). Recalling the definition of inclusion we simply obtain ${\hat{C}}^{\left(1\right)}\left({\hat{\epsilon }}^{\prime }-{\hat{\epsilon }}^{*}\right)\vec{n}{{|}_{\Sigma^{-}}={\hat{C}}^{\left(1\right)}{\hat{\epsilon }}^{\prime }\vec{n}|}_{\Sigma^{+}}$ and finally we get ${\hat{C}}^{\left(1\right)}{\hat{\epsilon }}^{\prime }\vec{n}{{|}_{\Sigma^{-}}-{\hat{C}}^{\left(1\right)}{\hat{\epsilon }}^{\prime }\vec{n}|}_{\Sigma^{+}}={\hat{C}}^{\left(1\right)}{\hat{\epsilon }}^{*}\vec{n}$. We use it in Equation (183), obtaining

(184) $$\begin{array}{c} V{\hat{T}}^{*}\hat{S}{\left[{\hat{C}}^{\left(1\right)}\right]}^{-1}{\hat{T}}^{**}=\int_{\Sigma }\left[{\hat{C}}^{\left(1\right)}{\hat{\epsilon }}^{\prime }\vec{n}{{|}_{\Sigma^{-}}-{\hat{C}}^{\left(1\right)}{\hat{\epsilon }}^{\prime }\vec{n}|}_{\Sigma^{+}}\right]{\vec{u}}^{\prime \prime }dS \end{array}$$

On $\Sigma^{-}$ we have ${\hat{T}}^{\prime }={\hat{C}}^{\left(1\right)}\left({\hat{\epsilon }}^{\prime }-{\hat{\epsilon }}^{*}\right)$ and on $\Sigma^{+}$ we have ${\hat{T}}^{\prime }={\hat{C}}^{\left(1\right)}{\hat{\epsilon }}^{\prime }$, therefore

(185) $$\begin{array}{ccc} & & V{\hat{T}}^{*}\hat{S}{\left[{\hat{C}}^{\left(1\right)}\right]}^{-1}{\hat{T}}^{**}\\ & = & \int_{\Sigma^{-}}\left({\hat{T}}^{\prime }+{\hat{T}}^{*}\right)\vec{n}{\vec{u}}^{\prime \prime }dS-\int_{\Sigma^{+}}{\hat{T}}^{\prime }\vec{n}{\vec{u}}^{\prime \prime }dS\\ & = & \int_{V}\frac{\partial }{\partial \vec{x}}\left[\left({\hat{T}}^{\prime }+{\hat{T}}^{*}\right){\vec{u}}^{\prime \prime }\right]dv+\int_{{ℜ}^{3}\backslash V}\frac{\partial }{\partial \vec{x}}\left[{\hat{T}}^{\prime }{\vec{u}}^{\prime \prime }\right]dv\\ & = & \int_{V}\left({\hat{T}}^{\prime }+{\hat{T}}^{*}\right){\hat{\epsilon }}^{\prime \prime }dv+\int_{{ℜ}^{3}\backslash V}{\hat{T}}^{\prime }{\hat{\epsilon }}^{\prime \prime }dv\\ & = & \int_{V}\left[{\hat{C}}^{\left(1\right)}\left({\hat{\epsilon }}^{\prime }-{\hat{\epsilon }}^{*}\right)+{\hat{T}}^{*}\right]{\hat{\epsilon }}^{\prime \prime }dv+\int_{{ℜ}^{3}\backslash V}{\hat{T}}^{\prime }{\hat{\epsilon }}^{\prime \prime }dv\\ & = & \int_{V}{\hat{\epsilon }}^{\prime }{\hat{C}}^{\left(1\right)}{\hat{\epsilon }}^{\prime \prime }dv+\int_{{ℜ}^{3}\backslash V}{\hat{\epsilon }}^{\prime }{\hat{C}}^{\left(1\right)}{\hat{\epsilon }}^{\prime \prime }dv\\ & = & \int_{{ℜ}^{3}}{\hat{\epsilon }}^{\prime }{\hat{C}}^{\left(1\right)}{\hat{\epsilon }}^{\prime \prime }dv \end{array}$$

We have now obtained a symmetric form (since ${\hat{C}}^{\left(1\right)}$ is symmetric). Therefore, the following dual relation is valid and it can be verified as above

(186) $$\begin{array}{c} V{\hat{T}}^{**}\hat{S}{\left[{\hat{C}}^{\left(1\right)}\right]}^{-1}{\hat{T}}^{*}=\int_{{ℜ}^{3}}{\hat{\epsilon }}^{\prime }{\hat{C}}^{\left(1\right)}{\hat{\epsilon }}^{\prime \prime }dv \end{array}$$

By comparison of Equations (185) and (186) we obtain

(187) $$\begin{array}{c} V{\hat{T}}^{*}\hat{S}{\left[{\hat{C}}^{\left(1\right)}\right]}^{-1}{\hat{T}}^{**}=V{\hat{T}}^{**}\hat{S}{\left[{\hat{C}}^{\left(1\right)}\right]}^{-1}{\hat{T}}^{*} \end{array}$$

which establishes the symmetry of $\hat{S}{\left[{\hat{C}}^{\left(1\right)}\right]}^{-1}$. The inverse tensor ${\left\{\hat{S}{\left[{\hat{C}}^{\left(1\right)}\right]}^{-1}\right\}}^{-1}={\hat{C}}^{\left(1\right)}{\hat{S}}^{-1}$ is again symmetric and, finally, the quantity ${\hat{C}}^{\left(1\right)}\left[{\hat{S}}^{-1}-\hat{I}\right]$ is symmetric since it is a sum of symmetric tensors.

### Lemma: The tensor ${\hat{C}}^{\left(1\right)}\left[{\hat{S}}^{-1}-\hat{I}\right]$ is positive definite

. We consider two similar situations as described in Figure 26. The first deals with an homogeneous medium with displacement prescribed on the boundary, while the second case considers the addition of an inhomogeneity without changing the fixed displacements on the external surface. No body forces are present in both schemes. We begin searching for the difference between the elastic energy stored in the two cases

(188) $$\begin{array}{c} \Delta E=\frac{1}{2}\int_{\Omega }\left({\hat{\epsilon }}_{b}{\hat{T}}_{b}-{\hat{\epsilon }}_{a}{\hat{T}}_{a}\right)dv \end{array}$$

We simply verify that

(189) $$\begin{array}{c} \int_{\Omega }{\hat{\epsilon }}_{a}{\hat{T}}_{a}dv=\int_{\Omega }{\hat{\epsilon }}_{b}{\hat{T}}_{a}dv \end{array}$$

(190) $$\begin{array}{c} \int_{\Omega }{\hat{\epsilon }}_{a}{\hat{T}}_{b}dv=\int_{\Omega }{\hat{\epsilon }}_{b}{\hat{T}}_{b}dv \end{array}$$

In order to verify Equation (189) we write the relation

(191) $$\begin{array}{c} \int_{\Omega }\left({\hat{\epsilon }}_{a}-{\hat{\epsilon }}_{b}\right){\hat{T}}_{a}dv=\int_{\Omega }\left(\frac{\partial {\vec{u}}_{a}}{\partial \vec{x}}{\hat{T}}_{a}-\frac{\partial {\vec{u}}_{b}}{\partial \vec{x}}{\hat{T}}_{a}\right)dv \end{array}$$

where $\frac{\partial {\vec{u}}_{a}}{\partial \vec{x}}{\hat{T}}_{a}=\frac{\partial {\vec{u}}_{a}{\hat{T}}_{a}}{\partial \vec{x}}$ since $\frac{\partial {\hat{T}}_{a}}{\partial \vec{x}}=0$ at equilibrium and similarly $\frac{\partial {\vec{u}}_{b}}{\partial \vec{x}}{\hat{T}}_{a}=\frac{\partial {\vec{u}}_{b}{\hat{T}}_{a}}{\partial \vec{x}}$. Therefore, we obtain

(192) $$\begin{array}{ccc} \int_{\Omega }\left({\hat{\epsilon }}_{a}-{\hat{\epsilon }}_{b}\right){\hat{T}}_{a}dv & = & \int_{\Omega }\left(\frac{\partial {\vec{u}}_{a}{\hat{T}}_{a}}{\partial \vec{x}}-\frac{\partial {\vec{u}}_{b}{\hat{T}}_{a}}{\partial \vec{x}}\right)dv=\int_{\Sigma }\left({\vec{u}}_{a}{\hat{T}}_{a}-{\vec{u}}_{b}{\hat{T}}_{a}\right)\vec{n}dS=0 \end{array}$$

since ${\vec{u}}_{a}={\vec{u}}_{b}$ on Σ. The dual relation given in Equation (190) can be verified with the same method. By inserting Equations (189) and (190) into Equation (188) we obtain

(193) $$\begin{array}{ccc} \Delta E & = & \frac{1}{2}\int_{\Omega }\left({\hat{\epsilon }}_{b}{\hat{T}}_{b}-{\hat{\epsilon }}_{a}{\hat{T}}_{a}\right)dv\\ & = & \frac{1}{2}\int_{\Omega }\left({\hat{\epsilon }}_{a}{\hat{T}}_{b}-{\hat{\epsilon }}_{b}{\hat{T}}_{a}\right)dv\\ & = & \frac{1}{2}\int_{\Omega \setminus V}\left({\hat{\epsilon }}_{a}{\hat{T}}_{b}-{\hat{\epsilon }}_{b}{\hat{T}}_{a}\right)dv+\frac{1}{2}\int_{V}\left({\hat{\epsilon }}_{a}{\hat{T}}_{b}-{\hat{\epsilon }}_{b}{\hat{T}}_{a}\right)dv\\ & = & \frac{1}{2}\int_{\Omega \setminus V}\left({\hat{\epsilon }}_{a}{\hat{C}}^{\left(1\right)}{\hat{\epsilon }}_{b}-{\hat{\epsilon }}_{b}{\hat{C}}^{\left(1\right)}{\hat{\epsilon }}_{a}\right)dv+\frac{1}{2}\int_{V}\left({\hat{\epsilon }}_{a}{\hat{T}}_{b}-{\hat{\epsilon }}_{b}{\hat{T}}_{a}\right)dv \end{array}$$

Since the stiffness tensor ${\hat{C}}^{\left(1\right)}$ is symmetric, we obtain the following general expression for the energy difference

(194) $$\begin{array}{c} \Delta E=\frac{1}{2}\int_{V}\left({\hat{\epsilon }}_{a}{\hat{T}}_{b}-{\hat{\epsilon }}_{b}{\hat{T}}_{a}\right)dv \end{array}$$

We now suppose that the prescribed displacement on Σ imposes a uniform strain in the first case of Figure 26; therefore, the second situation can be described by the Eshelby solution. With this additional hypothesis the energy difference can be rearranged as follows

(195) $$\begin{array}{ccc} \Delta E & = & -\frac{1}{2}\int_{V}\left({\hat{T}}_{a}{\hat{\epsilon }}_{b}-{\hat{\epsilon }}_{a}{\hat{T}}_{b}\right)dv\\ & = & -\frac{1}{2}\int_{V}\left({\hat{T}}_{a}{\hat{\epsilon }}_{b}-{\hat{T}}_{a}{\left({\hat{C}}^{\left(1\right)}\right)}^{-1}{\hat{C}}^{\left(2\right)}{\hat{\epsilon }}_{b}\right)dv\\ & = & -\frac{1}{2}\int_{V}{\hat{T}}_{a}\left(\hat{I}-{\left({\hat{C}}^{\left(1\right)}\right)}^{-1}{\hat{C}}^{\left(2\right)}\right){\hat{\epsilon }}_{b}dv\\ & = & -\frac{1}{2}\int_{V}{\hat{T}}_{a}{\hat{\epsilon }}^{*}dv \end{array}$$

having used Equation (180). Utilizing Equation (179) we obtain

(196) $$\begin{array}{c} \Delta E=-\frac{1}{2}\int_{V}{\hat{\epsilon }}_{a}{\hat{C}}^{\left(1\right)}{\left[{\left(\hat{I}-{\left({\hat{C}}^{\left(1\right)}\right)}^{-1}{\hat{C}}^{\left(2\right)}\right)}^{-1}-\hat{S}\right]}^{-1}{\hat{\epsilon }}_{a}dv \end{array}$$

From now on we suppose that the embedded inhomogeneity is a void (${\hat{C}}^{\left(2\right)}=0$) and, therefore, we obtain

(197) $$\begin{array}{c} \Delta E=E_{b}\left({\hat{\epsilon }}_{b}\right)-E_{a}\left({\hat{\epsilon }}_{a}\right)=-\frac{1}{2}\int_{V}{\hat{\epsilon }}_{a}{\hat{C}}^{\left(1\right)}{\left[\hat{I}-\hat{S}\right]}^{-1}{\hat{\epsilon }}_{a}dv \end{array}$$

We may now consider the variational formulation of the elasticity theory [85,86]. If we take into account a body without body forces and with prescribed displacements on the whole external surface, then the variational formulation leads to the minimum potential energy principle. We may apply this principle to the second case of Figure 26 (with a void). If the fields ${\vec{u}}_{b},{\hat{\epsilon }}_{b},{\hat{T}}_{b}$ correspond of the actual elastic fields in such a case, we have $E_{b}\left({\vec{u}}_{b},{\hat{\epsilon }}_{b},{\hat{T}}_{b}\right)⩽E_{b}\left(\vec{u},\hat{\epsilon },\hat{T}\right)$ where the fields $\vec{u},\hat{\epsilon },\hat{T}$ correspond to any displacement $\vec{u}$ matching the prescribed boundary. In particular we have $E_{b}\left({\hat{\epsilon }}_{b}\right)⩽E_{b}\left({\hat{\epsilon }}_{a}\right)$, where ${\hat{\epsilon }}_{a}$ is the strain in the first case of Figure 26. Moreover, we may write

(198) $$\begin{array}{ccc} E_{b}\left({\hat{\epsilon }}_{a}\right) & = & \frac{1}{2}\int_{\Omega \setminus V}{\hat{\epsilon }}_{a}{\hat{C}}^{\left(1\right)}{\hat{\epsilon }}_{a}dv+\frac{1}{2}\int_{V}{\hat{\epsilon }}_{a}{\hat{C}}^{\left(2\right)}{\hat{\epsilon }}_{a}dv\\ & = & \frac{1}{2}\int_{\Omega \setminus V}{\hat{\epsilon }}_{a}{\hat{C}}^{\left(1\right)}{\hat{\epsilon }}_{a}dv\\ & = & E_{a}\left({\hat{\epsilon }}_{a}\right)-\frac{1}{2}\int_{V}{\hat{\epsilon }}_{a}{\hat{C}}^{\left(1\right)}{\hat{\epsilon }}_{a}dv \end{array}$$

Summing up

(199) $$\begin{array}{ccc} E_{b}\left({\hat{\epsilon }}_{b}\right) & ⩽ & E_{b}\left({\hat{\epsilon }}_{a}\right)\\ E_{b}\left({\hat{\epsilon }}_{b}\right) & ⩽ & E_{a}\left({\hat{\epsilon }}_{a}\right)-\frac{1}{2}\int_{V}{\hat{\epsilon }}_{a}{\hat{C}}^{\left(1\right)}{\hat{\epsilon }}_{a}dv\\ E_{b}\left({\hat{\epsilon }}_{b}\right)-E_{a}\left({\hat{\epsilon }}_{a}\right) & ⩽ & -\frac{1}{2}\int_{V}{\hat{\epsilon }}_{a}{\hat{C}}^{\left(1\right)}{\hat{\epsilon }}_{a}dv \end{array}$$

Since ${\hat{\epsilon }}_{a}$ is uniform, combining Equations (197) and (199), we obtain

(200) $$\begin{array}{c} {\hat{\epsilon }}_{a}{\hat{C}}^{\left(1\right)}{\left[\hat{I}-\hat{S}\right]}^{-1}{\hat{\epsilon }}_{a}-{\hat{\epsilon }}_{a}{\hat{C}}^{\left(1\right)}{\hat{\epsilon }}_{a}⩾0 \end{array}$$

or

(201) $$\begin{array}{c} {\hat{T}}_{a}{\left[\hat{I}-\hat{S}\right]}^{-1}{\left[{\hat{C}}^{\left(1\right)}\right]}^{-1}{\hat{T}}_{a}-{\hat{T}}_{a}{\left[{\hat{C}}^{\left(1\right)}\right]}^{-1}{\hat{T}}_{a}⩾0 \end{array}$$

So, the tensor ${\left[\hat{I}-\hat{S}\right]}^{-1}{\left[{\hat{C}}^{\left(1\right)}\right]}^{-1}-{\left[{\hat{C}}^{\left(1\right)}\right]}^{-1}$ is positive definite.

For any tensor it is true that ${\left[I-A\right]}^{-1}=I+{\left[A^{-1}-I\right]}^{-1}$ and therefore we obtain

(202) $$\begin{array}{c} {\left[\hat{I}-\hat{S}\right]}^{-1}{\left[{\hat{C}}^{\left(1\right)}\right]}^{-1}-{\left[{\hat{C}}^{\left(1\right)}\right]}^{-1}={\left[{\hat{S}}^{-1}-\hat{I}\right]}^{-1}{\left[{\hat{C}}^{\left(1\right)}\right]}^{-1} \end{array}$$

Finally, the tensor ${\left[{\hat{S}}^{-1}-\hat{I}\right]}^{-1}{\left[{\hat{C}}^{\left(1\right)}\right]}^{-1}$ and its inverse ${\hat{C}}^{\left(1\right)}\left[{\hat{S}}^{-1}-\hat{I}\right]$ are symmetric and positive definite.

It is interesting to observe that all the results given in Appendix A can also be exactly applied to an anisotropic and homogeneous ellipsoidal inhomogeneity embedded in an anisotropic and homogeneous matrix. In this case, the Eshelby tensor $\hat{S}$ depends on the geometry and on ${\hat{C}}^{\left(1\right)}$ [84,98,99].

## Appendix B First Order Expansions for a Dispersion of Spheres

In this Appendix we present the first order expansions in the volume fraction of the effective nonlinear moduli $A_{eff}$, $B_{eff}$, $C_{eff}$, and $D_{eff}$ for a dispersion of spheres. In particular we consider four different cases where only one nonlinear modulus of the spheres (*A*, *B*, *C* or *D*) is different from zero. These solutions are coherent with the scheme represented in Figure 19. If C≠0 we obtain

(203) $$\left\{\begin{array}{c} C_{eff}^{C}=\frac{{\left(3K_{1}+4\mu_{1}\right)}^{3}}{{\left(4\mu_{1}+3K_{2}\right)}^{3}}Cc+O\left(c^{2}\right)\\ A_{eff}^{C}=B_{eff}^{C}=D_{eff}^{C}=0 \end{array}\right.$$

If B≠0 we have

(204) $$\left\{\begin{array}{ccc} B_{eff}^{B} & = & 25\frac{{\mu_{1}}^{2}}{{\left(6\mu_{2}K_{1}+12\mu_{2}\mu_{1}+9\mu_{1}K_{1}+8{\mu_{1}}^{2}\right)}^{2}}\\ & \times & \frac{{\left(3K_{1}+4\mu_{1}\right)}^{3}}{4\mu_{1}+3K_{2}}Bc+O\left(c^{2}\right)\\ C_{eff}^{B} & = & \frac{6\mu_{2}K_{1}+12\mu_{2}\mu_{1}+9\mu_{1}K_{1}+28{\mu_{1}}^{2}+15K_{2}\mu_{1}}{{\left(6\mu_{2}K_{1}+12\mu_{2}\mu_{1}+9\mu_{1}K_{1}+8{\mu_{1}}^{2}\right)}^{2}}\\ & \times & \frac{3\mu_{1}K_{1}-5K_{2}\mu_{1}+2\mu_{2}K_{1}+4\mu_{2}\mu_{1}-4{\mu_{1}}^{2}}{{\left(4\mu_{1}+3K_{2}\right)}^{3}}\\ & \times & {\left(3K_{1}+4\mu_{1}\right)}^{3}Bc+O\left(c^{2}\right)\\ A_{eff}^{B} & = & D_{eff}^{B}=0 \end{array}\right.$$

If D≠0 we obtain

(205) $$\left\{\begin{array}{ccc} C_{eff}^{D} & = & \frac{3\mu_{1}K_{1}-5K_{2}\mu_{1}+2\mu_{2}K_{1}+4\mu_{2}\mu_{1}-4{\mu_{1}}^{2}}{{\left(9\mu_{1}K_{1}+8{\mu_{1}}^{2}+6\mu_{2}K_{1}+12\mu_{2}\mu_{1}\right)}^{2}}\\ & \times & \frac{6\mu_{2}K_{1}+12\mu_{2}\mu_{1}+28{\mu_{1}}^{2}+9\mu_{1}K_{1}+15K_{2}\mu_{1}}{{\left(4\mu_{1}+3K_{2}\right)}^{3}}\\ & \times & {\left(3K_{1}+4\mu_{1}\right)}^{3}Dc+O\left(c^{2}\right)\\ D_{eff}^{D} & = & 25\frac{{\mu_{1}}^{2}}{{\left(9\mu_{1}K_{1}+8{\mu_{1}}^{2}+6\mu_{2}K_{1}+12\mu_{2}\mu_{1}\right)}^{2}}\\ & \times & \frac{{\left(3K_{1}+4\mu_{1}\right)}^{3}}{4\mu_{1}+3K_{2}}Dc+O\left(c^{2}\right)\\ A_{eff}^{D} & = & B_{eff}^{D}=0 \end{array}\right.$$

Finally, if A≠0 all the effective nonlinear moduli are different from zero and they can be eventually written as

(206) $$\left\{\begin{array}{ccc} A_{eff}^{A} & = & \frac{125{\left(3K_{1}+4\mu_{1}\right)}^{3}{\mu_{1}}^{3}Ac}{{\left(9K_{1}\mu_{1}+8{\mu_{1}}^{2}+6K_{1}\mu_{2}+12\mu_{2}\mu_{1}\right)}^{3}}+O\left(c^{2}\right)\\ B_{eff}^{A} & = & 25\frac{3K_{1}\mu_{1}-5K2\mu_{1}+2K1\mu_{2}+4\mu_{2}\mu_{1}-4{\mu_{1}}^{2}}{{\left(9K1\mu_{1}+8{\mu_{1}}^{2}+6K_{1}\mu_{2}+12\mu_{2}\mu_{1}\right)}^{3}}\\ & \times & \frac{{\left(3K_{1}+4\mu_{1}\right)}^{3}{\mu_{1}}^{2}Ac}{4\mu_{1}+3K_{2}}+O\left(c^{2}\right)\\ C_{eff}^{A} & = & 3\frac{{\left(3K_{1}\mu_{1}-5K_{2}\mu_{1}+2K_{1}\mu_{2}+4\mu_{2}\mu_{1}-4{\mu_{1}}^{2}\right)}^{2}}{{\left(9K_{1}\mu_{1}+8{\mu_{1}}^{2}+6K_{1}\mu_{2}+12\mu_{2}\mu_{1}\right)}^{3}}\\ & \times & \frac{10K_{2}\mu_{1}+2K_{1}\mu_{2}+3K_{1}\mu_{1}+4\mu_{2}\mu_{1}+16{\mu_{1}}^{2}}{{\left(4\mu_{1}+3K_{2}\right)}^{3}}\\ & \times & {\left(3K_{1}+4\mu_{1}\right)}^{3}Ac+O\left(c^{2}\right)\\ D_{eff}^{A} & = & 50\frac{{\mu_{1}}^{2}}{{\left(9K_{1}\mu_{1}+8{\mu_{1}}^{2}+6K_{1}\mu_{2}+12\mu_{2}\mu_{1}\right)}^{3}}\\ & \times & \frac{3K_{1}\mu_{1}-5K_{2}\mu_{1}+2K_{1}\mu_{2}+4\mu_{2}\mu_{1}-4{\mu_{1}}^{2}}{4\mu_{1}+3K_{2}}\\ & \times & {\left(3K_{1}+4\mu_{1}\right)}^{3}Ac+O\left(c^{2}\right) \end{array}\right.$$

It is interesting to remark that the more complicated cases, with all the nonlinear moduli of the spheres different form zero, can be simply handled by means of the superimposition of the four cases above considered.

## Appendix C First Order Expansions for a Dispersion of Cylinders

Here we present the first order expansions in the volume fraction of the effective nonlinear moduli $A_{eff}$, $B_{eff}$, $C_{eff}$, and $D_{eff}$ for a dispersion of cylinders. In particular we consider four different cases where only one nonlinear modulus of the cylinders (*A*, *B*, *C* or *D*) is different from zero. If C≠0 we have

(207) $$\left\{\begin{array}{ccc} C_{eff}^{C} & = & \frac{{\left(K_{1}+\mu_{1}\right)}^{3}}{{\left(K_{2}+\mu_{1}\right)}^{3}}Cc+O\left(c^{2}\right)\\ A_{eff}^{C} & = & B_{eff}^{C}=D_{eff}^{C}=0 \end{array}\right.$$

If B≠0 we obtain

(208) $$\left\{\begin{array}{ccc} B_{eff}^{B} & = & \frac{4{\left(K_{1}+\mu_{1}\right)}^{3}{\mu_{1}}^{2}B}{\left(K_{2}+\mu_{1}\right){\left(\mu_{1}K_{1}+\mu_{2}K_{1}+2\mu_{2}\mu_{1}\right)}^{2}}c+O\left(c^{2}\right)\\ C_{eff}^{B} & = & \frac{1}{2}\frac{2K_{2}\mu_{1}+\mu_{1}K_{1}+\mu_{2}K_{1}+2\mu_{2}\mu_{1}+2\mu_{1}^{2}}{{\left(\mu_{1}K_{1}+\mu_{2}K_{1}+2\mu_{2}\mu_{1}\right)}^{2}}\\ & \times & \frac{\mu_{1}K_{1}-2K_{2}\mu_{1}+\mu_{2}K_{1}+2\mu_{2}\mu_{1}-2\mu_{1}^{2}}{{\left(K_{2}+\mu_{1}\right)}^{3}}\\ & \times & {\left(K_{1}+\mu_{1}\right)}^{3}Bc+O\left(c^{2}\right)\\ A_{eff}^{B} & = & D_{eff}^{B}=0 \end{array}\right.$$

If D≠0 we have

(209) $$\left\{\begin{array}{ccc} C_{eff}^{D} & = & \frac{1}{2}\frac{K_{1}\mu_{1}-2K_{2}\mu_{1}+K_{1}\mu_{2}+2\mu_{2}\mu_{1}-2\mu_{1}^{2}}{{\left(K_{1}\mu_{1}+K_{1}\mu_{2}+2\mu_{2}\mu_{1}\right)}^{2}}\\ & \times & \frac{K_{1}\mu_{2}+K_{1}\mu_{1}+2\mu_{2}\mu_{1}+2K_{2}\mu_{1}+2\mu_{1}^{2}}{{\left(K_{2}+\mu_{1}\right)}^{3}}\\ & \times & {\left(K_{1}+\mu_{1}\right)}^{3}Dc+O\left(c^{2}\right)\\ D_{eff}^{D} & = & \frac{4{\left(K_{1}+\mu_{1}\right)}^{3}{\mu_{1}}^{2}Dc}{\left(K_{2}+\mu_{1}\right){\left(K_{1}\mu_{1}+K_{1}\mu_{2}+2\mu_{2}\mu_{1}\right)}^{2}}+O\left(c^{2}\right)\\ A_{eff}^{D} & = & B_{eff}^{D}=0 \end{array}\right.$$

Finally, if A≠0, as predicted by the scheme represented in Figure 19, all the effective nonlinear moduli are different from zero and the final expressions are given below

(210) $$\left\{\begin{array}{ccc} A_{eff}^{A} & = & 8\frac{{\left(K_{1}+\mu_{1}\right)}^{3}{\mu_{1}}^{3}}{{\left(K_{1}\mu_{1}+K_{1}\mu_{2}+2\mu_{2}\mu_{1}\right)}^{3}}Ac+O\left(c^{2}\right)\\ B_{eff}^{A} & = & 2\frac{{\mu_{1}}^{2}}{{\left(K_{1}\mu_{1}+K_{1}\mu_{2}+2\mu_{2}\mu_{1}\right)}^{3}}\\ & \times & \frac{K_{1}\mu_{1}-2K_{2}\mu_{1}+K_{1}\mu_{2}+2\mu_{2}\mu_{1}-2\mu_{1}^{2}}{K_{2}+\mu_{1}}\\ & \times & {\left(K_{1}+\mu_{1}\right)}^{3}Ac+O\left(c^{2}\right)\\ C_{eff}^{A} & = & \frac{1}{4}\frac{{\left(K_{1}\mu_{1}-2K_{2}\mu_{1}+K_{1}\mu_{2}+2\mu_{2}\mu_{1}-2\mu_{1}^{2}\right)}^{2}}{{\left(K_{1}\mu_{1}+K_{1}\mu_{2}+2\mu_{2}\mu_{1}\right)}^{3}}\\ & \times & \frac{K_{1}\mu_{2}+2\mu_{2}\mu_{1}+K_{1}\mu_{1}+4K_{2}\mu_{1}+4\mu_{1}^{2}}{{\left(K_{2}+\mu_{1}\right)}^{3}}\\ & \times & {\left(K_{1}+\mu_{1}\right)}^{3}Ac+O\left(c^{2}\right)\\ D_{eff}^{A} & = & 4\frac{{\mu_{1}}^{2}}{{\left(K_{1}\mu_{1}+K_{1}\mu_{2}+2\mu_{2}\mu_{1}\right)}^{3}}\\ & \times & \frac{K_{1}\mu_{1}-2K_{2}\mu_{1}+K_{1}\mu_{2}+2\mu_{2}\mu_{1}-2\mu_{1}^{2}}{\left(K_{2}+\mu_{1}\right)}\\ & \times & {\left(K_{1}+\mu_{1}\right)}^{3}Ac+O\left(c^{2}\right) \end{array}\right.$$
